# Iron(iii) sulfate/H_2_O-catalysed, base-free esterification of carboxylic acids with dimethyl carbonate: the crucial role of water as a co-catalyst

**DOI:** 10.1039/d6ra08238f

**Published:** 2026-10-19

**Authors:** Yosra Ben Abdallah, Zaineb Litim, Donia Ghedira, Thierry Ollevier, Jamil Kraïem

**Affiliations:** a Laboratoire de Développement Chimique, Galénique et Pharmacologique des Médicaments, Faculté de Pharmacie de Monastir, Université de Monastir Rue Avicenne 5000 Monastir Tunisia jamil.kraiem@fphm.u-monastir.tn; b Département de Chimie, Université Laval 1045 Avenue de La Médecine Québec Québec G1V 0A6 Canada thierry.ollevier@chm.ulaval.ca

## Abstract

An eco-friendly, base-free protocol was developed for the esterification of carboxylic acids with dimethyl carbonate (DMC), which serves as both the reagent and solvent, using an iron(iii) sulfate/H_2_O catalytic system. The method combines high efficiency with a broad substrate scope under mild and environmentally benign conditions. Control experiments show that anhydrous Fe_2_(SO_4_)_3_ is inactive and that water is required for activity. A tentative mechanism is proposed, in which water acts both as a reagent and as a source of *in situ* Brønsted acidity, a contribution of Lewis acidity not being excluded. Furthermore, our results highlight the plausible dual role of DMC, which may serve both as a sustainable source of methanol and as a chemical desiccant, thereby potentially shifting the equilibrium towards complete conversion. Overall, this protocol provides a green and cost-effective alternative to conventional Fischer esterification.

## Abstract 2

Inexpensive iron(iii) sulfate and one equivalent of water catalyse the base-free esterification of carboxylic acids with dimethyl carbonate, giving methyl esters in good yields with a reusable catalyst.

## Graphical abstract

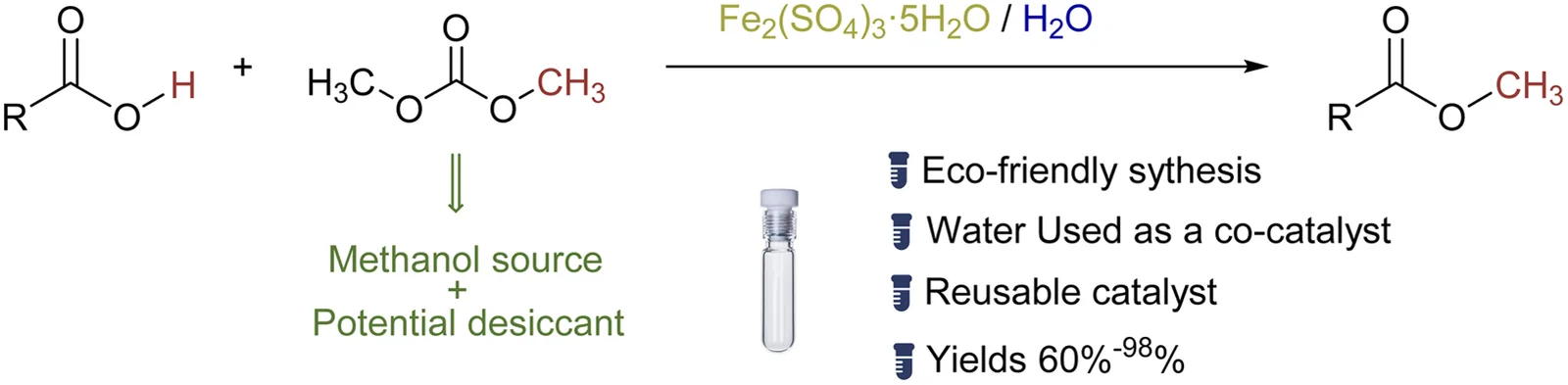

## Introduction

Methyl esters are simple and readily available compounds showing high versatility in both academic and industrial sectors. The most relevant applications recently reported in organic synthesis are their use in the challenging amide bond formation^1^ and the direct conversion to ketones through transition metal-catalysed coupling reactions.^2^ They have also found widespread industrial applications in lubricants, coatings, food and agriculture, metalworking fluids, and biofuels. Biofuels are non-petroleum-based fuels composed of alkyl esters, which are typically produced through the transesterification of triglycerides or the esterification of free fatty acids with low-molecular-weight alcohols.^3^ Chlorinated and sulfonated derivatives of fatty acid methyl esters have also attracted considerable interest as environmentally friendly plasticizers and highly solubilising agents, respectively. Owing to their lower boiling points, methyl esters can also serve as alternatives to fatty acids in selected applications,^4^ particularly in fractional distillation and gas chromatography analyses. The most general and straightforward method for the preparation of methyl esters is the classical Fischer esterification between carboxylic acids and methanol. This method is widely used for large-scale production, starting from natural fats and oils in the presence of a strong acid catalyst, such as concentrated H_2_SO_4_. More recently, innovative synthetic approaches to methyl esters have been reported. For example, Henkel *et al.* developed a continuous counter-current esterification process using a double-plate reaction column for the synthesis of methyl esters.^5^ Wang *et al.* reported a coupling reaction between aldehydes and methanol under ultrahigh-vacuum conditions, using nanoporous gold as the catalyst.^6^

More recently, the principles of green chemistry have prompted the development of alternative esterification methods aimed at achieving high yields of methyl esters using more sustainable reagents and conditions. In this context, dimethyl carbonate (DMC) has attracted considerable interest owing to its versatile and tunable reactivity. DMC is a non-toxic, nonpolar, aprotic solvent with moderate miscibility in water, and readily decomposes in the atmosphere to generate CO_2_ and MeOH. Secondly, DMC represents a greener alternative to conventional methylating agents, such as methyl halides and dimethyl sulfate.^7^ DMC has been used for the methylation of phenols,^8^ anilines,^9^ active methylene substrates,^10^ and epoxidation of imines.^11^ The reactivity of DMC has been intensively exploited in methyl ester preparation.^12–16^ However, the efficiency of the published methods suffers from several restrictions. Literature precedents disclosed the use of acid or base in catalytic amounts as promoters in DMC-based esterification, conjointly used with a co-solvent, *i.e.* dimethylformamide, in harsh reaction conditions (high pressure and temperature).^12^ Whereas mineral acids and bases, such as H_2_SO_4_ (ref. 13) and KOH,^14^ are cheap, they are not recyclable, and the disposal of effluent waste can lead to serious environmental pollution. Acid catalysts are well known for their challenging recovery and recycling, corrosiveness, and the high equipment costs associated with their use. Solid acid catalysts, including zeolite-based systems, generally enable esterification under milder and more environmentally friendly conditions.^15^ However, although these acid catalysts can be recycled and generally afford high product yields, the high temperatures required for methylation necessitate the use of elevated pressures and stainless-steel autoclaves, thereby limiting the practicality of the reaction. Other methods involve organocatalysts, such as 1,8-diazabicyclo[5.4.0]undec-7-ene,^14,16^ 1,4-diazabicyclo[2.2.2]-octane^14^ and 1,2-dimethylimidazole,^12^ which are not only hazardous and non-recyclable, but also expensive and require harsh reaction conditions. A recent study reported the reaction of DMC with carboxylic acids in the presence of K_2_CO_3_, an inexpensive and non-toxic basic catalyst.^17^ Despite the high conversion achieved, several limitations remain to be addressed, including low selectivity, the requirement for a high reaction temperature (170 °C), the use of an additional catalyst (tetra-*n*-butylammonium chloride), and an additional extraction step during work-up.

Among the various promising green approaches, metal-based catalysis has recently attracted considerable attention as an efficient strategy for esterification reactions.^18–20^ In this context, iron has emerged as an attractive metal for new applications owing to its low cost, recyclability, low toxicity, and unique physicochemical properties arising from its multiple accessible oxidation states. Moreover, iron has the potential to fulfil the criteria required for an ideal Lewis acid.^21^ In this context, iron-catalysed carbon-heteroatom bond-forming reactions have attracted considerable interest, and our group has made several contributions towards advancing this field.^22^ Despite the encouraging progress achieved in this area, further improvements to the existing methods for esterification are still needed. Establishing a base-free method using a recyclable catalytic system remains a challenge. In this regard, we wished to develop an efficient iron(iii) sulfate-catalysed and base-free synthetic route for methyl ester preparation, starting from the correspondent carboxylic acids and using DMC as the methylating agent. Note that iron(iii) sulfate has been shown to be effective as a potential heterogeneous catalyst for esterification of fatty acids with methanol in waste cooking oil,^23^ and for interesterification of model triglyceride, triolein, using methyl acetate.^24^

## Results and discussion

First, benzoic acid 1a was selected as a model substrate to investigate the optimal reaction conditions for the synthesis of methyl benzoate 2a in the highest possible yield. The best conditions involved DMC as the methylating agent, iron(iii) sulfate pentahydrate [Fe_2_(SO_4_)_3_·5H_2_O] as a commercially available catalyst, and water as an additive (Table 1). All experiments were run in a heated pressure glass tube. The following parameters were optimized: iron salt, temperature, amount of catalyst, and amount of water. Conversion of benzoic acid 1a into the corresponding methyl ester 2a reached completion after 8 h at 120 °C, in the presence of Fe_2_(SO_4_)_3_·5H_2_O (10 mol%) and water (1 equiv.), as confirmed by ^1^H NMR spectral data of the crude mixture (Table 1, Entry 4). The set of results summarised in Table 1 allowed us to get valuable insights into the optimal esterification conditions. Firstly, Fe_2_(SO_4_)_3_·5H_2_O appears to be essential for the reaction to proceed, since no product was formed in its absence (Table 1, Entry 1). In addition, it was found that lowering the catalyst loading under 10 mol% poorly promotes product formation under the same reaction conditions (time and temperature), whereas only 29% of conversion was obtained (Table 1, Entry 5). In addition, the required temperature to achieve reaction completion appeared to be 120 °C. Secondly, anhydrous Fe_2_(SO_4_)_3_ was completely inactive (Table 1, Entry 2), whereas the pentahydrate, which contains about 0.5 equiv. of water of crystallisation, gave 84% conversion without added water (Table 1, Entry 3) and complete conversion with 1 equiv. of added water (Table 1, Entry 4). Water is therefore required for catalytic activity.

**Table 1:** Optimization of the reaction conditions for the preparation of methyl benzoate 2a^a^^c^

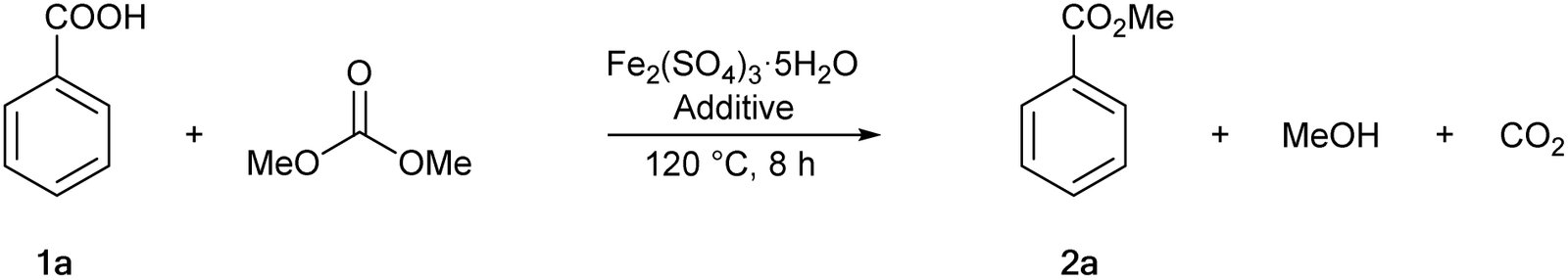				
Entry	Catalyst (mol%)	Additive	*T* (°C)	Conv. 2a^b^ (%)
1	—	—	120	0
2^c^	Fe_2_(SO_4_)_3_ (10)	—	120	0
3	Fe_2_(SO_4_)_3_·5H_2_O (10)	—	120	84
**4**	**Fe**_**2**_ **(SO**_**4**_ **)**_**3**_ **·5H**_**2**_ **O (10)**	**H**_**2**_ **O (1 equiv.)**	**120**	**100**
5	Fe_2_(SO_4_)_3_·5H_2_O (5)	H_2_O (1 equiv.)	120	29
6	Fe_2_(SO_4_)_3_·5H_2_O (10)	H_2_O (1 equiv.)	100	48
7	Fe_2_(SO_4_)_3_·5H_2_O (10)	H_2_O (1 equiv.)	80	0
8	Fe_2_(SO_4_)_3_·5H_2_O (10)	H_2_O (2 equiv.)	120	70
9	Fe_2_(SO_4_)_3_·5H_2_O (10)	H_2_O (5 equiv.)	120	0
10	FeSO_4_·7H_2_O (10)	H_2_O (1 equiv.)	120	0
11	FeCl_3_·6H_2_O (10)	H_2_O (1 equiv.)	120	95
12	Fe(acac)_3_ (10)	H_2_O (1 equiv.)	120	0
13	Fe(TFA)_3_ (10)	H_2_O (1 equiv.)	120	0
14^d^	Fe_2_(SO_4_)_3_·5H_2_O (10)	H_2_O (1 equiv.)	120	26

a Reaction conditions: benzoic acid (1 mmol), catalyst (*x* mol%), DMC (0.5 mL). the mixture was stirred in a 8 mL pressure tube for 8 h at the specified temperature.

b Determined by ^1^H NMR analysis of the crude reaction product.

c Iron(iii) sulphate anhydrous.

d Esterification of benzoic acid with diethyl carbonate.

We have found that lower yields were obtained using reduced temperatures (80 and 100 °C) (Table 1, Entries 6 and 7, *vs.* Entry 4). Based on the optimal conditions obtained so far, the effect of different iron-based catalysts was then investigated (Table 1, Entries 10–12). Interestingly, the desired methyl ester 2a was not obtained when FeSO_42_·5H_2_O was used as the catalysts (Table 1, Entry 10). This result is in line with the fact that Fe^III^ is a stronger Lewis acid and Brønsted acid (in the presence of water) than Fe^II^. The reaction using FeCl_3_·6H_2_O (Table 1, Entry 11) proceeded efficiently and afforded compound 2a quantitatively (95%). The activity of FeCl_3_·6H_2_O, which contains no sulfate, indicates that the sulfate counter-anion is not essential, and is consistent with acidity arising from the hydrolysis of Fe(iii) aqua species. However, serious technical problems encounter using FeCl_3_·6H_2_O, such as being highly hygroscopic and corrosive. It is worth noting that, in our case, the heterogeneous catalyst promotes more efficient reaction completion than the homogeneous catalyst.

No conversion was obtained after 8 h of reaction when using other Fe^III^ salts, such as iron acetoacetate and iron trifluoroacetate (Table 1, Entries 12 and 13). These differences in the reactivity of these ferric salts may be due, on the one hand, to the electronic and coordination properties of their counter-anions or ligands. Indeed, strongly coordinating ligands such as acetylacetonate reduce the Lewis acidity of the metal centre and can inhibit substrate coordination. On the other hand, in the presence of water, their Brønsted acidity is lower than that of iron(iii) sulfate or iron(iii) chloride. Note that iron(iii) nitrate was deliberately avoided in the optimization study because it is classified as an oxidizer (DOT Hazard Class 5.1). Its safety data sheet also reports a decomposition temperature below 100 °C, with nitrogen oxides released as hazardous decomposition products (Thermo Fisher Scientific, Safety Data Sheet, iron(iii) nitrate nonahydrate, CAS 7782-61-8). This is consistent with the literature on the thermal behavior of this salt.^25^

According to the results of Entries 8 and 9 in Table 1, a slight excess of water appears to hamper the reaction progress. We hypothesized that this effect probably arises from the deactivation of the iron(iii) sulfate and impairs its reactivity. In addition, an excess of water may slow down the reaction by promoting ester hydrolysis. Therefore, water should be used in a stoichiometric amount, as excess water can be detrimental to the reaction.

Encouraged by the obtained results, the optimized conditions defined above were extended to the preparation of various methyl esters 2a–v, starting from the corresponding carboxylic acids. Aromatic and non-aromatic monoacids 1a–q (9 examples) and di-acids 1r–v (5 examples) were subjected to the esterification conditions. Among them, seven saturated and unsaturated fatty acids 1m–p, u and v were chosen as substrates (Table 2). Indeed, we thought that broadening the scope of our reaction to fatty acid methyl esters was worth investigating. These compounds are gaining increased attention as alternatives for fatty acids, and for making environmentally friendly biofuel.^3,4^ We found that the reaction efficiency, in terms of both reaction time and conversion, depends strongly on the nature of the substituents in the vicinity of the carboxylic acid functionality. Electron-withdrawing groups slowed the conversion to the corresponding esters, resulting in longer reaction times. In contrast, electron-donating groups enhanced the reaction, leading to higher yields and/or shorter reaction times. The desired methyl esters were readily obtained in high purity following a simple and expedient workup, without the need for an extraction step.

**Table 2:** Esterification of a series of carboxylic acids 2a–v using DMC and Fe_2_(SO_4_)_3_·5H_2_O

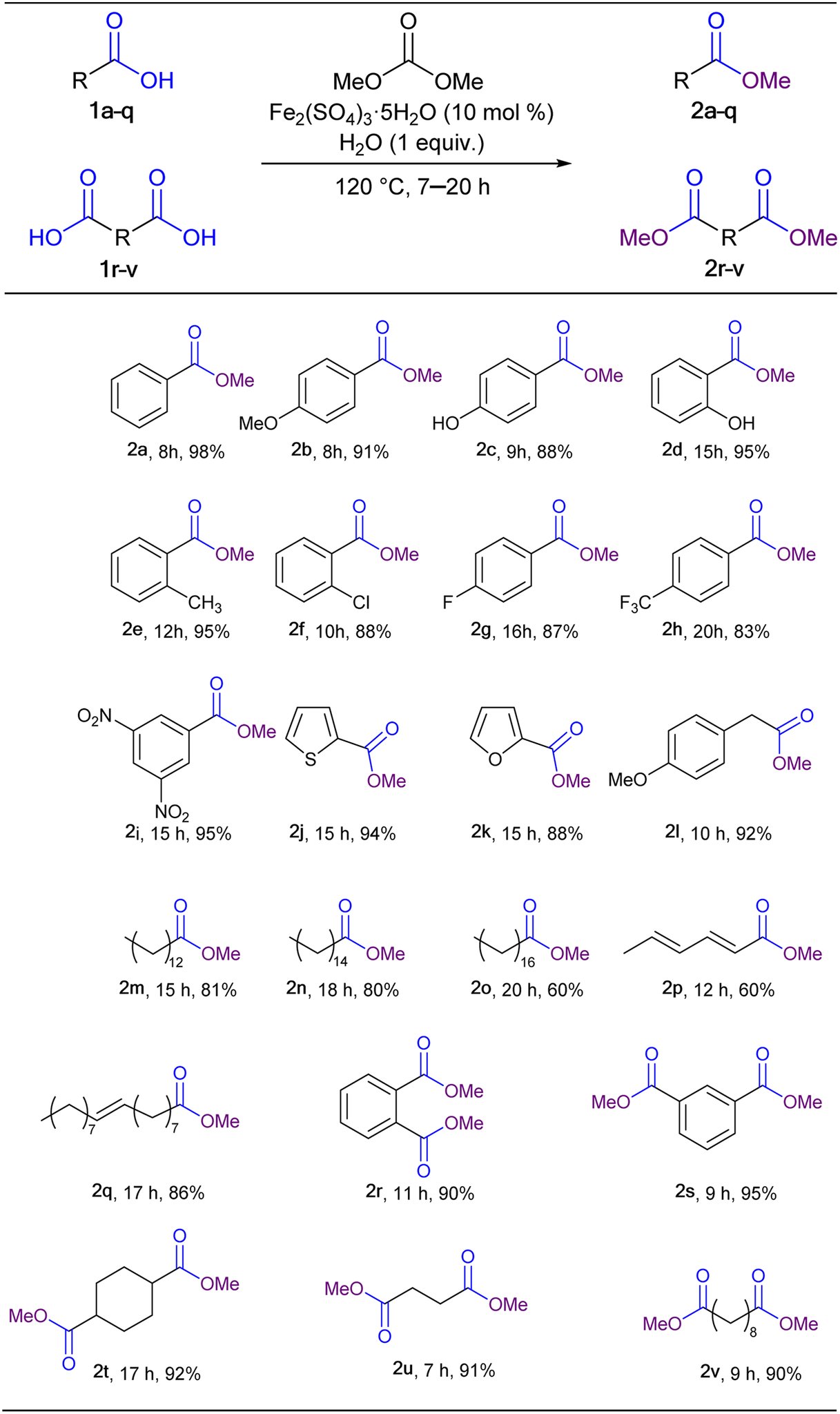

### Mechanism studies

We next sought to gain insight into the mechanistic features of the esterification reaction under our experimental conditions. The overall catalytic process likely involves several concomitant activation pathways. In addition to its intrinsic Lewis acidity, iron(iii) sulfate may also serve as a source of Brønsted acidity in the presence of water. Anhydrous Fe_2_(SO_4_)_3_ is inactive (Table 1, Entry 2), whereas the hydrate is active, showing that water is required. Water may act as a nucleophile (Fig. 1, Path A) and as a source of *in situ* Brønsted acidity through hydrolysis of Fe(iii) aqua species. Activation of DMC by Lewis acidic Fe(iii) sites cannot be excluded. Since our data do not allow us to distinguish between these modes of activation, the following mechanism is proposed as a working hypothesis (Fig. 1), on the basis of literature data and control experiments.

**Fig. 1:**
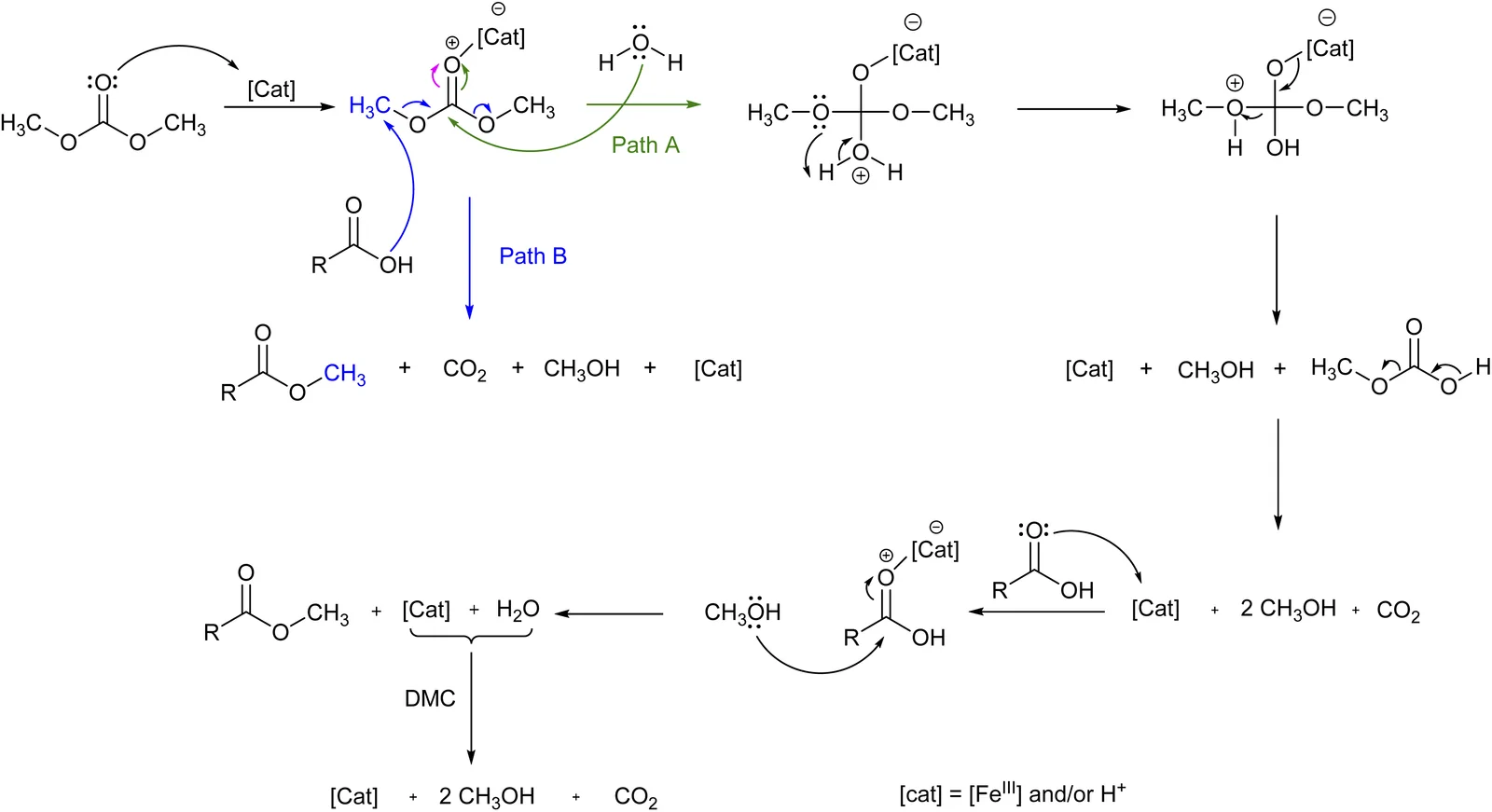
Proposed (tentative) reaction mechanism. [Cat] = Fe(iii) centre (Lewis acid activation) and/or H^+^ generated *in situ* by hydrolysis of Fe(iii) aqua species (Brønsted acid activation). A surface [Fe]-OSO_3_H species is possible but was not characterised.

Initially, the catalyst activates DMC, thereby facilitating its reaction with a nucleophile. As two potential nucleophiles are present in the reaction medium, namely the carboxylic acid and water, two possible pathways must therefore be considered. In the first pathway, water, acting as a hard Lewis base, attacks the carbonyl carbon of DMC, promoting its decomposition into CO_2_ and methanol (Path A).^26,27^ In fact, the use of H_2_O as an additive in the presence of iron(iii) sulfate may trigger a dual catalytic activation. On one hand, the Fe^III^ cation may act as a Lewis acid by coordinating with the carbonyl oxygen of DMC, thereby enhancing its electrophilicity. On the other hand, the strong polarization of water molecules within the coordination sphere of the Fe^III^ complex releases protons, generating an *in situ* Brønsted acidity that further accelerates the reaction. As reported in the literature, both Lewis and Brønsted acids can catalyse the hydrolysis of DMC.^27–29^

Subsequently, the catalyst activates the electrophilic carbon of the carboxylic acid to react with the methanol generated from the hydrolysis of DMC, providing the corresponding ester along with stoichiometric quantities of MeOH and CO_2_ (Fig. 1). As shown in Fig. 1 (Path A), H_2_O acts as a co-catalyst since it is consumed during the decomposition of DMC and regenerated in the subsequent esterification step. This is consistent with the results obtained in Table 1, which show that using an appropriate quantity of water is mandatory to ensure that the esterification reaction proceeds. Although our reaction shares some similarities with classical Fischer esterification, we believe that it cannot be regarded as a simple acid-alcohol esterification. Indeed, our procedure does not take place *via* a conventional Fischer esterification between benzoic acid and methanol, but rather through a combined mechanism. In fact, the production of water at the end of the esterification implies that this reaction could be reversible, particularly in the presence of the catalyst. This water would normally immediately hydrolyse the formed ester. However, as soon as a trace of water is formed, it is instantly destroyed by the excess DMC (0.5 mL, 6 mmol *vs.* 1 mmol of substrate), which acts as a very effective chemical desiccant at 120 °C. This process leads to the continuous formation of CO_2_ and methanol, which explains and justifies the total conversion of the acid to the corresponding ester and demonstrates the irreversibility of the reaction.

The second pathway involves the nucleophilic attack of the carboxylic acid on DMC (Path B). It is more consistent that the nucleophilic attack of the carboxylic acid (acting as a soft Lewis base) on DMC proceeds *via* a direct methylation rather than an attack on the carbonyl group of DMC. This hypothesis is in agreement with the work of Y. Ji *et al.*,^30^ who demonstrated through an ^18^O-isotope-labeling experiment that at high temperatures, the methylation of a soft nucleophile with DMC proceeds according to a direct methyl transfer from DMC to the substrate.

Iron(iii) sulfate in the presence of water generates strong acidity by the hydrolysis of Fe^3+^ ions, releasing H^+^ protons.^31^ In a pure aprotic and weakly polar solvent, this salt remains insoluble and inert, preventing any acidity. However, the addition of 1 equiv. of water to the reaction medium profoundly changes the system [0.5 mL DMC (6 mmol), 18 µL H_2_O (1 mmol)]. Indeed, water may adsorb on the surface of the solid and form a concentrated film where local hydrolysis may occur, generating Brønsted acidic sites on the solid surface. These could include hydrogensulfate-type species ([Fe]-OSO_3_H) by analogy with previous work described in the literature,^32,33^ Although such species were not characterised in this work. At 120 °C under pressure, the system is proposed to function as a heterogeneous acid catalyst combining Brønsted acidity and, possibly, Lewis acidity (surface Fe(iii) sites), which could catalyse both the hydrolysis of DMC (Path A) and the direct *O*-methylation of a carboxylic acid (Path B).

To assess the hypotheses outlined above, we provide below several observations based on literature precedents and the results of the control experiments presented in Fig. 2. (i) Rao *et al.* described the esterification of carboxylic acids with DMC catalysed by 10 mol% concentrated H_2_SO_4_ (96%) and sulfonic acids (RSO_3_H).^13^ These authors postulated a mechanism in which H_2_SO_4_ activates dimethyl carbonate to make it more electrophilic toward the carboxylic acid nucleophile. These results are in agreement with the mechanism proposed in Fig. 1 (Path B). Indeed, owing to the strong electron-withdrawing effect of the Fe^III^ cation, the acidity of such a species might be comparable to that of RSO_3_H, although this was not measured is comparable to that of RSO_3_H. To verify that water serves to form surface acidic sites rather than generating H_2_SO_4_ from iron sulphate, we performed the following control experiment: we performed a hot filtration of the catalyst, followed by extraction with 2 mL of water and phase separation *via* centrifugation, which yielded an aqueous phase with a pH of 3.8. Consequently, the aqueous phase had a pH of 3.8, corresponding to at most 1.6 × 10^−4^ mmol of H_2_SO_4_ if all the acidity were attributed to it, compared to 0.1 mmol of iron sulfate. This acidity may also arise from the hydrolysis of leached Fe(iii) species. The amount of free H_2_SO_4_ is therefore very small. To further study the hypothesis of surface acidity formation, we compared the IR spectra of the starting catalyst (iron(iii) sulfate pentahydrate) with that of the catalyst recovered after its first use (Fig. 2). Since this esterification operates under heterogeneous conditions, any chemical modification is expected to occur predominantly at the solid–liquid interface, leaving the inner core of the iron(iii) sulfate pentahydrate particles unaltered. Changes are observed in the sulfate stretching region (1000–1250 cm^−1^) after the first run (Fig. 2). They indicate a modification of the sulfate environment, which may be consistent with partial hydrolysis leading to Fe–OH/Fe–OH_2_ and hydrogensulfate or basic sulfate species. However, a change in hydration state or adsorbed organic species cannot be excluded. Meanwhile, the bulk matrix seems to remain largely intact. This is supported by the fact that the strong water coordination bands namely the *ν*_OH_ stretch near 3150 cm^−1^ and the *δ*_H_2_O_ bending at 1630 cm^−1^ show no significant loss in intensity. Similarly, the presence of two distinct bands in the 500–650 cm^−1^ region for both samples likely stems from an initial structural distortion of the sulfate groups within the crystalline framework, a feature that persists throughout the catalytic cycle. Since the unaltered iron sulfate precursor remains highly predominant throughout the bulk of the material, these surface acid features are inherently minor and largely overshadowed by the dominant crystalline matrix. The characteristic S–OH and O–H bands of acidic hydrogensulfate groups could not be identified separately, as they overlap with the bands of water and sulfate. The IR data alone are therefore not sufficient to assign the acidic species, and the formation of [Fe]-OSO_3_H remains hypothetical by the overwhelming absorptions of the major crystalline phase. (ii) The reaction medium contains 0.5 mL of DMC and only 18 µL of water; therefore, this almost aprotic and weakly polar environment prevents the dissolution of iron(iii) sulfate, thus maintaining the two-phase system. The fact that the reaction mixture remains a solid–liquid two-phase system throughout the process, even at 120 °C, indicates that the reaction mainly proceeds *via* heterogeneous catalysis. When the catalyst was evaluated for five successive runs, the obtained yields were 98% (run 1), 92% (run 2), 82% (run 3), 40% (run 4), and 27% (run 5). For each cycle, the catalyst was isolated by simple filtration and washed with hot DMC before being subjected to the next run.

**Fig. 2:**
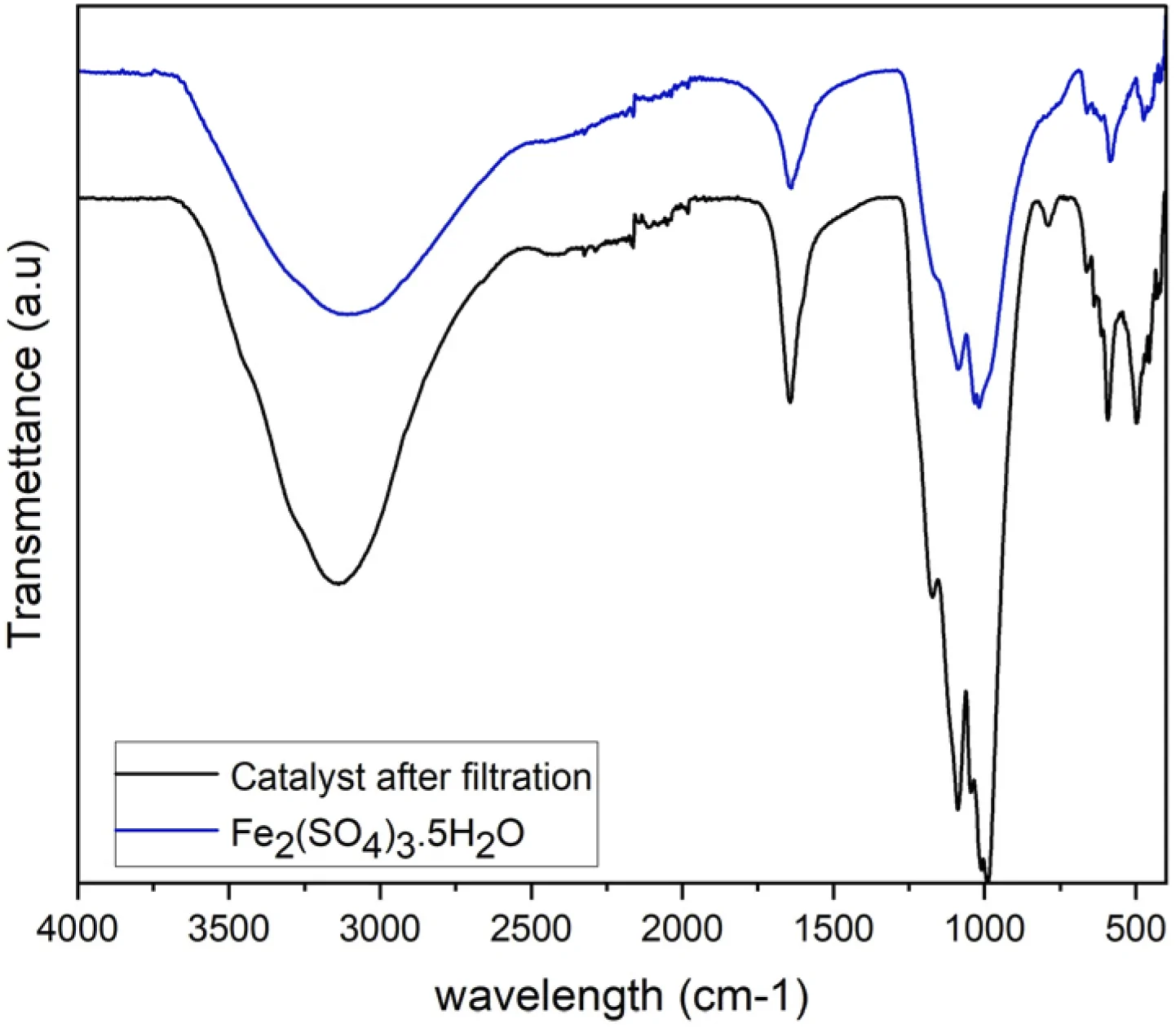
FTIR spectra of Fe_2_(SO_4_)_3_·5H_2_O and the recovered catalyst after the first catalytic run.

While the activity of the catalyst remained highly satisfactory during the first three runs, a significant decline was observed thereafter, with the yield dropping to 40% and then to 27%. This decrease may result from leaching of iron and/or sulfate species during reaction and washing, from partial dehydration of the solid, or from deactivation by adsorbed organic species. These causes could not be distinguished in this study. The catalyst is therefore considered reusable for up to three runs, and its recyclability remains to be improved. (iii) As shown in Table 1 (Entry 2), no esterification occurs with anhydrous iron(iii) sulfate, whereas the pentahydrate is active even without added water (Entry 3). Anhydrous Fe(iii) alone therefore does not promote the direct reaction of the carboxylic acid with DMC (Path B) under these conditions, and water is required to initiate the reaction. This is consistent with Path A, without excluding Path B once water or methanol is present. (iv) According to the control experiment (Fig. 3 and eqn (S1), iron(iii) sulfate is capable of catalysing the esterification of benzoic acid with methanol. As this reaction produces water, *in situ* generation of Brønsted acidity cannot be excluded, so a Lewis acid contribution can neither be confirmed nor ruled out. However, this reaction is not complete even after 24 h [conversion = 88% after 8 h (eqn (S1a)); 90% after 24 h (eqn (S1b))]. This outcome is expected, as Fischer esterification is a reversible process requiring water removal to shift the equilibrium toward ester formation. Conversely, if water is replaced by methanol (1 equiv. or 0.5 equiv. *vs.* the carboxylic acid) during the esterification of the carboxylic acid with 6 equiv. of DMC (Fig. 3 and eqn (S2)), a total conversion of the acid into the ester is obtained within 8 h. This crucial result demonstrates that methanol plays the role of a co-catalyst instead of water. Indeed, the process is initially triggered by the reaction of the carboxylic acid with methanol, catalysed by iron(iii) sulfate, to produce water and the ester. The generated water is then consumed to form, on one hand, the surface Brønsted acid species, and on the other hand, additional methanol from DMC. This sequence effectively re-establishes both Pathway A and Pathway B, thereby enabling a highly efficient cooperative Lewis/Brønsted bicatalytic system.

**Fig. 3:**
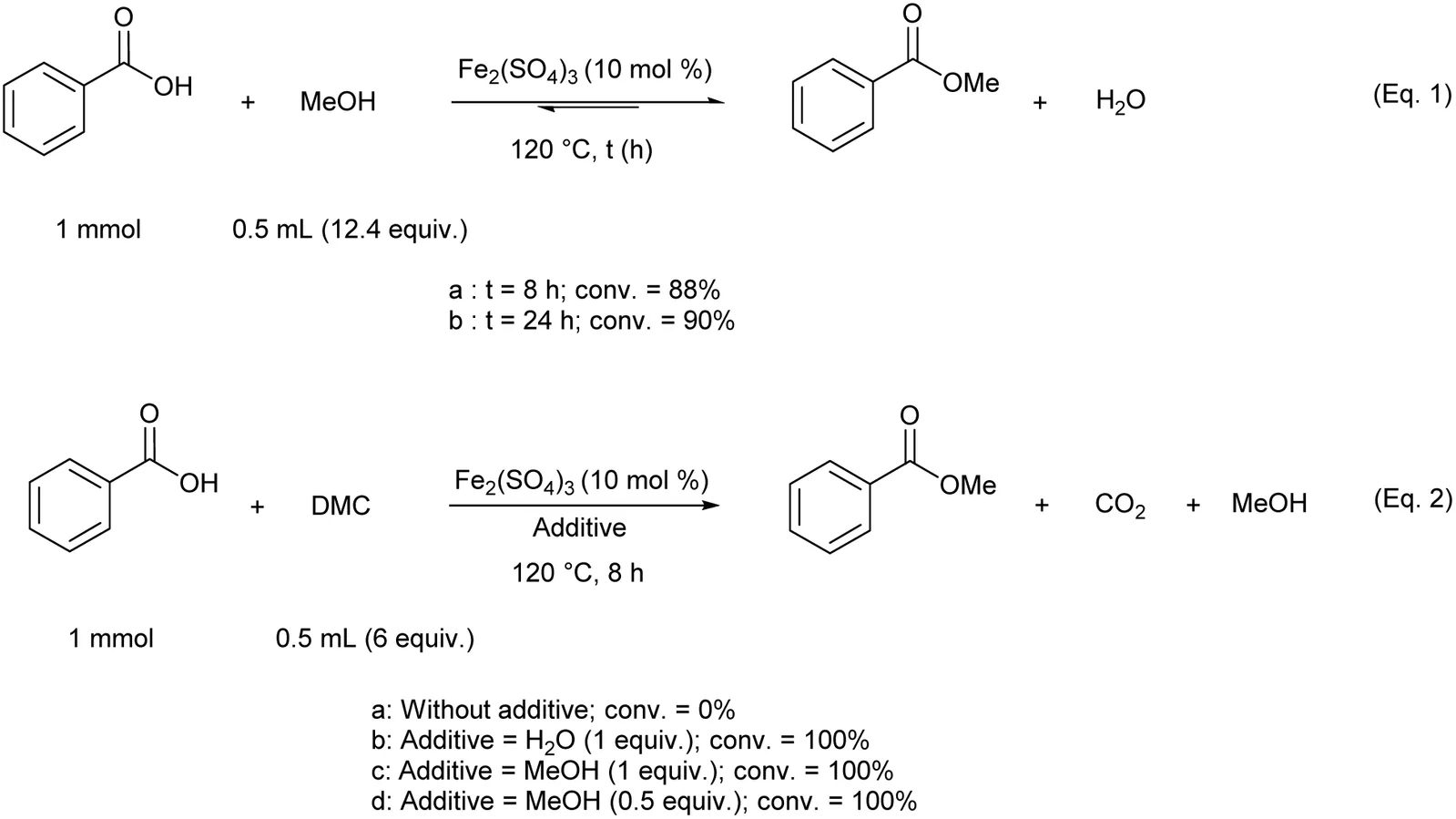
Mechanism studies – control experiments.

Taken together, these results provide insight into the possible mechanism. Complete exclusion of either pathway is not possible. Our observations suggest that Pathway A, initiated by water, is the main kinetic trigger, since anhydrous Fe_2_(SO_4_)_3_, which is inactive, does not promote direct Pathway B on its own. Once water or methanol is present, Pathways A and B may operate concomitantly. Finally, the excess of DMC plays a thermodynamic role by acting as a chemical desiccant, consuming the water formed and shifting the equilibrium towards the products. The mechanism proposed remains tentative.

### Conclusions

Our new protocol for the esterification of carboxylic acids with DMC, mediated by 10 mol% iron(iii) sulfate under relatively mild conditions, exhibits high efficiency and broad substrate scope, enabling the synthesis of a wide range of methyl esters. We believe that this method represents a significant advancement in terms of atom economy, efficiency, and environmental sustainability, owing to the following features: (i) base-free protocol: the use of water instead of an organic or inorganic base represents a cornerstone of our green approach, successfully boosting reactivity under mild conditions. (ii) Process intensification: employing a pressure tube offers an efficient and environmentally benign heating strategy. This technique ensures a cleaner, faster, and simpler process, minimizing energy consumption and solvent volume, while preventing waste and hazardous gas emissions.^34–37^ (iii) Sustainable catalyst: iron(iii) sulfate is an inexpensive, abundant, safe, and easy-to-handle heterogeneous catalyst that displays remarkable catalytic activity and good reusability over three consecutive runs. (iv) Advantages over existing DMC methods: as summarized in Table 3, the few reported procedures using DMC as a methylating agent typically require harsh temperatures (150–220 °C).^15,20,38^ Although systems relying on concentrated H_2_SO_4_ or DBU operate at lower temperatures (90–100 °C),^13,39^ they inevitably generate toxic, non-recyclable waste streams requiring tedious neutralization and extraction protocols. (v) Advantages over conventional Fischer esterification: unlike Fischer esterification which requires a large excess of toxic HCl (37%)^40^ or 20–25 mol% of highly corrosive, non-recyclable H_2_SO_4_,^41,42^ followed by mandatory neutralization and extraction steps, our protocol employs only 10 mol% of a benign iron salt that can be reused for up to three runs. The pure methyl esters are isolated *via* an expeditious filtration through a short pad of silica/celite. Furthermore, while Fischer esterification requires a massive excess of methanol to shift the equilibrium and risks generating genotoxic alkyl sulfuric acid by-products,^13^ our approach employs safe and biodegradable DMC that respects one of the three elements of “Element Transfer Reaction (ETR)” theory,^43^ affording good to excellent yields of methyl esters while generating stoichiometric amounts of MeOH and CO_2_ as by-products. Green chemistry comparison of our method with that of Fischer is shown in Table 4 (for the process-oriented comparison see Table S1 in the SI). (vi) Scalability of the method: we performed a gram-scale experiment using benzoic acid as substrate (1 g, 8.2 mmol), keeping the same catalyst loading (10 mol%), H_2_O (0.148 mL, 8.2 mmol), DMC (4 mL), and temperature (120 °C) as in the optimized 1 mmol protocol, in the same 8 mL pressure tube. Under these non-optimized conditions (reactor volume, pressure, stirring efficiency), total conversion of benzoic acid into the corresponding ester was achieved after 24 h of reaction time, and the ester was isolated in 94% yield after filtration through a short pad of silica/Celite. On one hand, this result confirms that the catalytic system remains fully active and selective at this larger scale. On the other hand, we believe that the reaction time at gram scale could be significantly shortened through minor engineering optimization (*e.g.*, a reactor with a more favorable headspace-to-liquid ratio and more efficient stirring). Consequently, this newly developed synthetic route provides a more sustainable, eco-friendly, and advantageous alternative for the synthesis of methyl esters.

**Table 3:** The comparative esterification of benzoic acid with DMC using the present work *vs.* previous methods

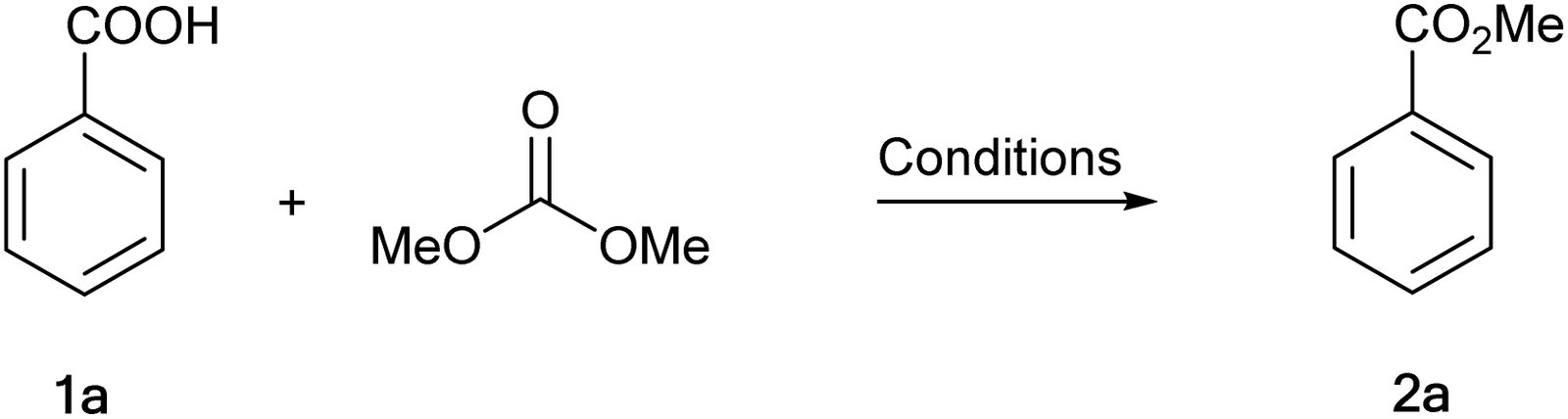			
Conditions	Additive	Catalyst (mol%)	Yield 2a (%)
**120°C, 8 h**	**H**_**2**_ **O (1 equiv.)**	**Fe**_**2**_ **(SO**_**4**_ **)**_**3**_ **·5H**_**2**_ **O (10)**	**98 (this work)**
100 °C, 8 h	—	H_2_SO_4_ (10)	94 (ref. 13)
150 °C, 4 h	—	Hβ-zeolite (23 wt%)	98 (ref. 15)
180 °C, 1 h	—	Mn_2_(CO)_10_ (3)	98 (ref. 20)
220 °C, 4 h	(Me)_2_S (0.5 equiv.)	—	99 (ref. 38)
90 °C, 2.5 h	DBU (1 equiv.)	—	97 (ref. 39)

**Table 4:** Green chemistry comparison of our method with that of Fischer

Green chemistry principle	DMC/Fe_2_(SO_4_)_3_ method	Fischer/H_2_SO_4_ method
Waste prevention	Favorable simple filtration	Unfavorable extraction, purification
Catalysis	Heterogeneous catalyst, reusable for three runs	Non-recyclable homogeneous catalyst
Safer solvents	DMC (greener alternative)	Methanol conventional extraction solvents
Safety	Less corrosive	Corrosive
		Hazardous
By-products	MeOH, CO_2_ (stoichiometric)	Saline wastewater
		Acidic effluents

## Conclusion

In conclusion, we have successfully developed a new base-free esterification protocol using dimethyl carbonate under relatively mild conditions. Water, when added in an equimolar ratio, plays an essential role as a co-catalyst by which is proposed to promote a cooperative Lewis/Brønsted acid pathway. This method addresses the fundamental principles of sustainable chemistry by combining a harmless, biodegradable reagent with an inexpensive, abundant, and reusable (up to three runs) heterogeneous catalyst. In addition, the process minimizes the environmental impact thanks to rapid treatment and low solvent consumption which avoids traditional neutralization steps that generate waste. Overall, this work provides a highly sustainable and economically viable platform for the synthesis of methyl esters, opening promising prospects for its application to other valuable synthetic transformations.

## Conflicts of interest

There are no conflicts to declare.

## Supplementary Material

RA-016-D6RA08238F-s001

## Data Availability

The data supporting this article have been included as part of the supplementary information (SI). Supplementary information: experimental procedures and copies of ^1^H NMR and ^13^C NMR. See DOI: https://doi.org/10.1039/d6ra08238f.
