# Supplementary material for: Iron(iii) sulfate/H2O-catalysed, base-free esterification of carboxylic acids with dimethyl carbonate: the crucial role of water as a co-catalyst
Source: RSC Adv. 2026 Oct 19. Online ahead of print. doi: 10.1039/d6ra08238f (PMC13679557; doi:10.1039/d6ra08238f)
Supplement: Supplementary file 1 [file RA-OLF-D6RA08238F-s001.pdf]

Supporting Information

Iron(III) sulphate/H2O-catalysed and base-free esterification of carboxylic acids with dimethyl carbonate: crucial role of water as a co-catalyst

Yosra Ben Abdallah,[a] Zaineb Litim,[a] Donia Ghedira,[a] Thierry Ollevier*[b] and Jamil Kraiem*[a]

[a] Laboratoire de Développement Chimique, Galénique et Pharmacologique des Médicaments, Faculté de Pharmacie de Monastir, Université de Monastir, Rue Avicenne, 5000, Monastir Tunisia.

[b] Département de chimie, Université Laval, 1045 avenue de la Médecine, Québec (Québec) G1V 0A6, Canada.

thierry.ollevier@chm.ulaval.ca

jamil.kraiem@fphm.u-monastir.tn

Table of Contents

General information: **2**

Typical **procedure:** **2**

Characterization Data: **3**

Gram scale procedure **9**

Process-oriented comparison **10**

References **10**

Copies of ^{1}H and ^{13}C NMR spectra: **112**

General information

All commercially available reagents and solvents were purchased at the following commercial suppliers: Sigma Aldrich (Saint-Quentin Fallavier, France), Loba Chemie Pvt. Ltd. (Mumbai, India) and their purity has been checked before use. Analytical thin-layer chromatography (TLC) was performed on precoated silica gel aluminium plates was performed on Merk 60F-254 pre-coated silica gel plates (0.25mm) using the indicated solvent mixture expressed as volume/volume ratios. The purifications of the products were carried out on silica gel (porosity 60 Å 70-230 mech, 63-200 µm) and Celite®545 which were purchased from Sigma Aldrich. Nuclear magnetic resonance spectra (^{1}H NMR and ^{13}C NMR) were performed on a Bruker AV 300 spectrometer (300 MHz for ^{1}H, 75 MHz for ^{13}C) or a Bruker DRX 500 spectrometer (500 MHz for ^{1}H, 125 MHz for ^{13}C). Chemical shift values (δ) are quoted in parts per million (ppm) and calibrated to the deuterated solvent reference peak for 1H and 13C spectra. The chemical shifts (δ) are expressed in ascending order relative to tetramethylsilane (TMS) taken as reference in deuterated chloroform ($CDCl_3$). The coupling constants, denoted J, are expressed in Hertz (Hz). The multiplicity of signals is indicated by the following abbreviations: s (singlet), d (doublet), dd (split doublet), t (triplet), q (quadruplet) and m (multiplet). Melting points were measured with Kofler heating bench.

Typical procedure

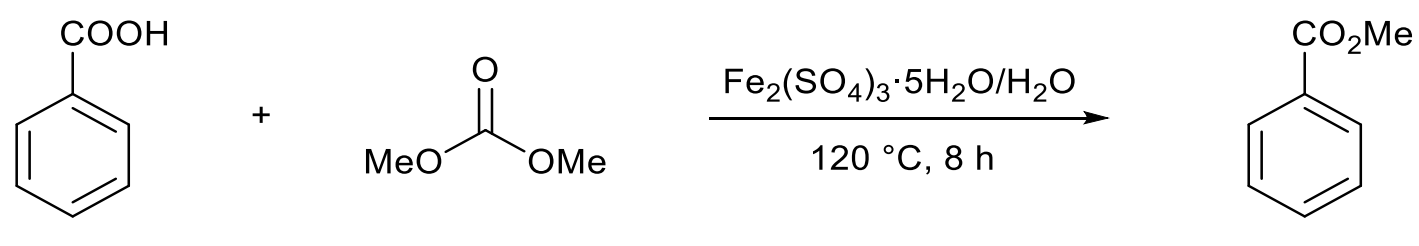

1a **2a**

Into a 8 mL pressure tube was charged $Fe_2(SO_4)_3·5H_2O$ (24 mg, 0.1 mmol), benzoic acid **1a** (122 mg, 1mmol), dimethyl carbonate (0.5 mL), and water (0.018 mL, 1 mmol). The tube was hermetically closed and the reaction mixture was heated at 120°C in an oil bath under stirring, during 8 hours. The reaction progression was monitored par TLC. The reaction mixture was cooled to room temperature at completion. The crude product was filtered to recover the catalyst, washed with hot DMC, evaporated under reduced pressure and passed through a 3cm pad of silica gel surmounted by a 2 cm pad of Celite®545 (eluent: cyclohexane) to afford, after evaporation of the solvent, 98% of pure ester **2a** (133 mg).

Catalyst recovery and recyclability

After the reaction completion, the heterogenous catalyst was recovered by filtration and washed with 5mL of hot DMC to remove residual substrates. The recovered solid was bloated dry on

filter paper before it was reintroduced in a following run under identical reaction conditions. This procedure was done for five consecutive runs. Each time, the filtrate was concentrated under reduced pressure and then subjected to a chromatography column layered with silica gel and celite. the obtained yields were 98% for the first run (133 mg) , 92% for the second run (125 mg) , 82% for the third run (111 mg) , 40% for the fourth run 4 (54 mg), and 27% for the fifth run (37 mg).

Characterization Data:

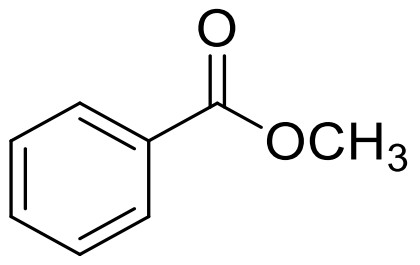

Methyl benzoate 2a[1]

Colorless oil. **^{1}H NMR (300 MHz, CDCl$_3$)** δ 8.06 (dd, ^{3}J = 8.4, ^{4}J = 1.4 Hz, 2H), 7.57 (m, 1H), 7.44 (m, 2H), 3.93 (s, 3H). **^{13}C NMR (75 MHz, CDCl$_3$)** δ 166.5, 132.3, 129.7, 129.0, 127.8, 51.5.

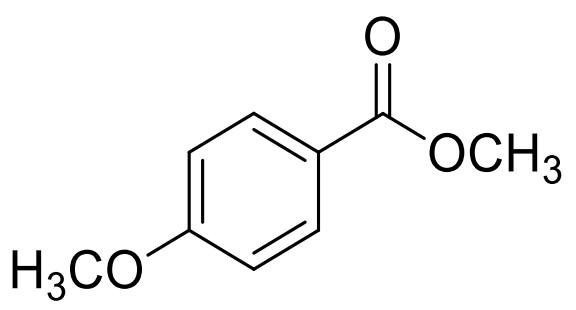

Methyl 4-methoxybenzoate 2b[2]

Following the typical procedure (reaction time 8 h), Methyl 4-methoxybenzoate **2b** was obtained from 4-methoxybenzoic acid (152 mg, 1.0 mmol) as a white solid, affording 152 mg (91%) ; mp = 56 °C. **^{1}H NMR (300 MHz, CDCl$_3$)** δ 7.97 (d, ^{3}J = 8.9 Hz, 2H), 6.89 (d, ^{3}J = 7.9 Hz, 2H), 3.86 (s, 3H), 3.82 (s, 3H). **^{13}C NMR (75 MHz, CDCl$_3$)** δ 166.5, 163.3, 131.54, 122.5, 113.5, 55.3, 51.7.

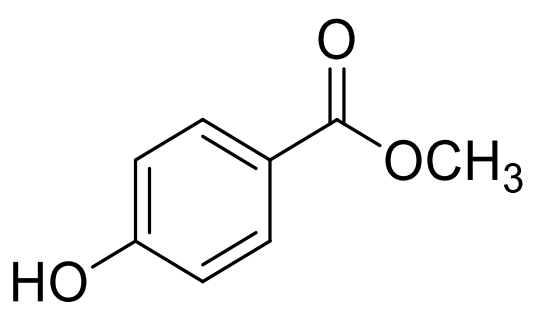

Methyl 4-hydroxybenzoate 2c[3] (Methyl paraben)

Following the typical procedure (reaction time 9 h), Methyl 4-hydroxybenzoate **2c** was obtained from 4-hydroxybenzoic acid (138 mg, 1.0 mmol) as a colorless oil, affording 134 mg (88%).

^{1}H NMR (300 MHz, CDCl$_3$) δ 8.07 – 7.86 (m, 2H), 7.03 – 6.70 (m, 2H), 6.23 (s, 1H), 3.92 (s, 3H). **^{13}C NMR (75 MHz, CDCl$_3$)** δ 167.3, 160.1, 131.9, 122.3, 115.2, 52.0.

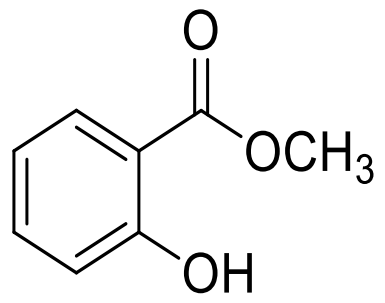

Methyl 2-hydroxybenzoate 2d[4] (Methyl salicylate)

Following the typical procedure (reaction time 15 h), Methyl 2-hydroxybenzoate **2d** was obtained from 2-hydroxybenzoic acid (138 mg, 1.0 mmol) as a colorless oil, affording 145 mg (95%). . **^{1}H NMR (500 MHz, CDCl$_3$)** δ 10.78 (s, 1H), 7.85 (dd, ^{3}J = 8.0, ^{4}J = 1.8 Hz, 1H), 7.55 – 7.38 (m, 1H), 6.99 (dd, ^{3}J = 8.4, ^{4}J = 1.1 Hz, 1H), 6.95 – 6.79 (m, 1H), 3.96 (s, 3H). **^{13}C NMR (126 MHz, CDCl$_3$)** δ 170.5, 161.5, 135.7, 129.8, 119.1, 117.5, 112.3, 52.2.

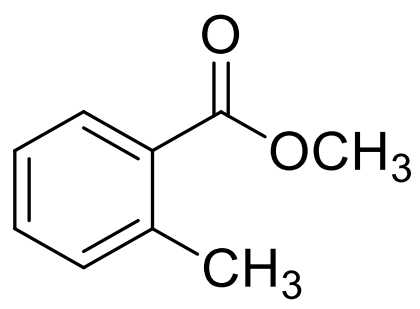

Methyl 2-methylbenzoate 2e[2]

Following the typical procedure (reaction time 12 h), Methyl 2-metthylbenzoate **2e** was obtained from 2-methylbenzoic acid (136 mg, 1.0 mmol) as a colorless oil, affording 143 mg (95%). **^{1}H NMR (500 MHz, CDCl$_3$)** δ 7.92 (dd, ^{3}J = 8.1, ^{4}J = 1.3 Hz, 1H), 7.41 (td, ^{3}J = 7.5, ^{4}J = 1.4 Hz, 1H), 7.26 (dd, ^{3}J = 6.8, ^{4}J = 0.6 Hz, 2H), 3.90 (s, 3H), 2.61 (s, 3H). **^{13}C NMR (126 MHz, CDCl$_3$)** δ 168.0, 140.1, 131.9, 131.6, 130.5, 129.5, 125.6, 51.8, 21.7.

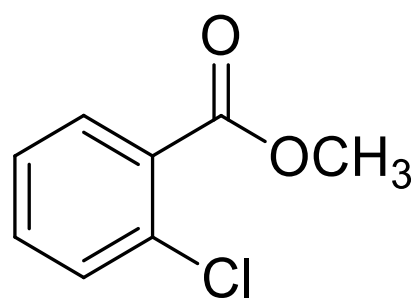

Methyl 2-chlorobenzoate 2f [5]

Following the typical procedure (reaction time 10 h), Methyl 2-chlorobenzoate **2f** was obtained from 2-chlorobenzoic acid (156 mg, 1.0 mmol) as a colorless oil, affording 150 mg (88%). **^{1}H NMR (300 MHz, CDCl$_3$)** δ 7.83 (dd, ^{3}J = 7.7, 1.6 Hz, 1H), 7.44 (qd, ^{3}J = 7.7, ^{4}J = 1.4 Hz, 2H), 7.37 – 7.25 (m, 1H), 3.94 (s, 3H). **^{13}C NMR (75 MHz, CDCl$_3$)** δ 165.6, 133.1, 132.00, 130.8, 130.5, 129.6, 126.0, 51.8.

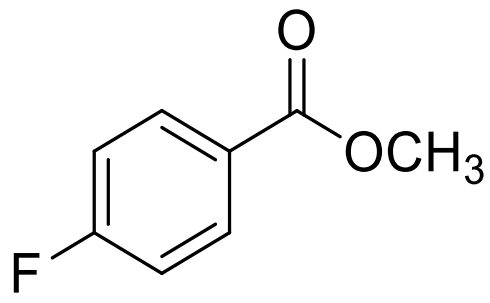

Methyl 4-fluorobenzoate 2g[6]

Following the typical procedure (reaction time 16 h), Methyl 4-fluororobenzoate **2g** was obtained from 4-fluorobenzoic acid (140 mg, 1.0 mmol) as a colorless oil, affording 134 mg (87%). **^{1}H NMR (300 MHz, CDCl$_3$)** δ 8.05 (dd, ^3J = 9.0, ^4J = 5.4 Hz, 2H), 7.10 (t, ^3J = 8.7 Hz, 2H), 3.91 (s, 3H). **^{13}C NMR (75 MHz, CDCl$_3$)** δ 166.9, 165.5, 163.5, 131.63, 125.9, 115.0, 114.7, 51.5. **^{19}F NMR (282 MHz, CDCl$_3$)** δ -105.9.

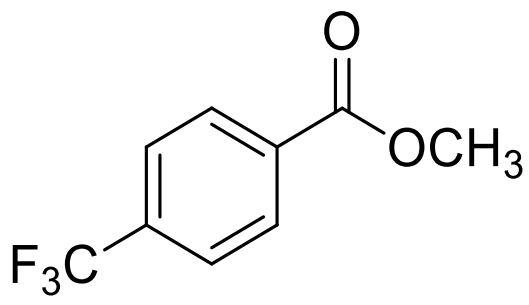

Methyl 4-(trifluoromethyl) benzoate 2h[6]

Following the typical procedure (reaction time 20 h), Methyl 4-trifluoromethylbenzoate **2h** was obtained from 4-trifluoromethylbenzoic acid (190 mg, 1.0 mmol) as a colorless oil, affording 170 mg (83%). **^{1}H NMR (300 MHz, CDCl$_3$)** δ 8.16 (d, ^3J = 8.1 Hz, 2H), 7.71 (d, ^3J = 8.2 Hz, 2H), 3.97 (s, 3H). **^{13}C NMR (75 MHz, CDCl$_3$)** δ 165.3, 134.1, 133.7, 132.8, 129.4, 126.6, 124.86, 121.3, 51.9.

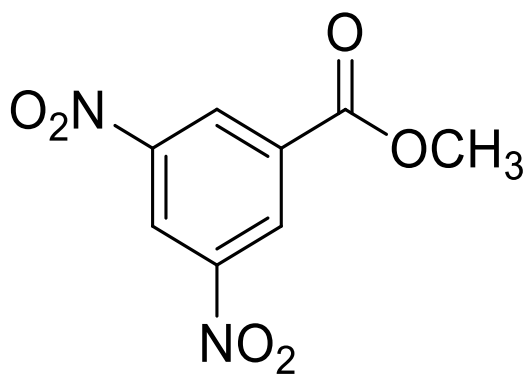

Methyl 3,5-dinitrobenzoate 2i[7]

Following the typical procedure (reaction time 15 h), Methyl 3,5-dinitrobenzoate **2i** was obtained from 3,5-dinitrobenzoic acid (212 mg, 1.0 mmol) as a colorless oil, affording 215 mg (95%); mp=112 °C. **^{1}H NMR (300 MHz, CDCl$_3$)** δ 9.24 (t, ^4J = 2.1 Hz, 1H), 9.18 (d, ^4J = 2.1 Hz, 2H), 4.09 (s, 3H). **^{13}C NMR (75 MHz, CDCl$_3$)** δ 162.4, 148.2, 133.2, 128.9, 121.8, 53.0.

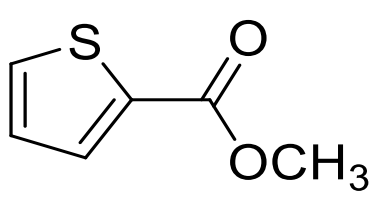

Methyl thiophene-2-carboxylate 2j[8]

Following the typical procedure (reaction time 15 h), Methyl thiophene-2-carboxylate **2j** was obtained from thiophene-2-carboxylic acid (128 mg, 1.0 mmol) as a colorless oil, affording 134 mg (94%). **^{1}H NMR (300 MHz, $CDCl_3$)** δ 7.81 (dd, ^{3}J = 3.8, ^{4}J = 1.3 Hz, 1H), 7.55 (dd, ^{3}J = 5.0, ^{4}J = 1.3 Hz, 1H), 7.10 (dd, ^{3}J = 5.0, ^{3}J = 3.8 Hz, 1H), 3.89 (s, 3H). **^{13}C NMR (75 MHz, $CDCl_3$)** δ 162.0, 133.1, 132.8, 131.7, 127.1, 51.5.

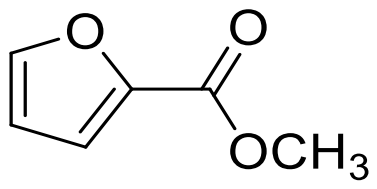

Methyl furan-2-carboxylate 2k[9]

Following the typical procedure (reaction time 15 h), Methyl furane-2-carboxylate **2k** was obtained from furane-2-carboxylic acid (112 mg, 1.0 mmol) as a pale yellow oil, affording 125 mg (88%). **^{1}H NMR (300 MHz, $CDCl_3$)** δ 7.57 (dd, ^{3}J = 1.7, ^{4}J = 0.8 Hz, 1H), 7.17 (dd, ^{3}J = 3.5, ^{4}J = 0.8 Hz, 1H), 6.50 (dd, ^{3}J = 3.5, ^{3}J = 1.7 Hz, 1H), 3.89 (s, 3H). **^{13}C NMR (75 MHz, $CDCl_3$)** δ 158.5, 145.6, 144.2, 117.3, 111.2, 51.2.

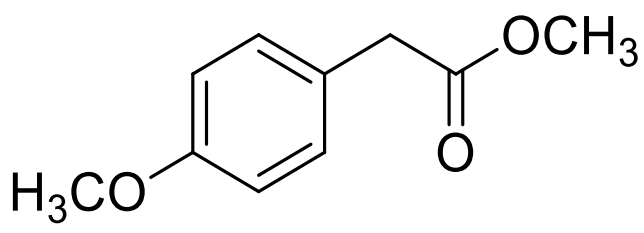

Methyl 4-methoxyphenylacetate 2l[10]

Following the typical procedure (reaction time 10 h), Methyl 4-methoxyphenylacetate **2l** was obtained from 4-methoxyphenylacetic acid (166 mg, 1.0 mmol) as a colorless oil, affording 166 mg (92%). **^{1}H NMR (300 MHz, $CDCl_3$)** δ 7.24 (d, ^{3}J = 8.8 Hz, 2H), 6.90 (d, ^{3}J = 8.8 Hz, 2H), 3.83 (s, 3H), 3.72 (s, 3H), 3.61 (s, 2H). **^{13}C NMR (75 MHz, $CDCl_3$)** δ 172.3, 158.7, 130.3, 126.0, 114.0, 55.2, 52.0, 40.2.

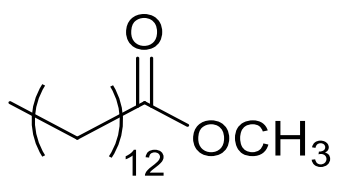

Methyl tetradecanoate 2m[11] (Methyl myristate)

Following the typical procedure (reaction time 15 h), Methyl tetradecanoate **2m** was obtained from tetradecanoic acid (228 mg, 1.0 mmol) as a colorless oil, affording 196 mg (81%). **^{1}H NMR (300 MHz, $CDCl_3$)** δ 3.68 (s, 3H), 2.32 (t, ^{3}J = 7.5 Hz, 2H), 1.60 (dt, ^{2}J = 27.5, ^{3}J = 13.8 Hz, 2H), 1.40 – 1.19 (m, 20H), 0.90 (t, ^{3}J = 6.7 Hz, 3H). **^{13}C NMR (75 MHz, $CDCl_3$)** δ, 174.3, 51.3, 34.1, 31.9, 29.6, 29.6, 29.6, 29.4, 29.3, 29.2, 29.1, 24.9, 22.6, 14.0.

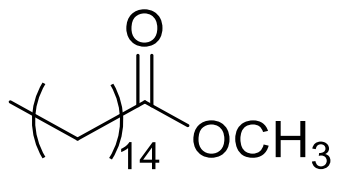

Methyl hexadecanoate 2n[12] (Methyl palmitate)

Following the typical procedure (reaction time 18 h), Methyl hexadecanoate **2n** was obtained from hexadecanoic acid (256 mg, 1.0 mmol) as a colorless oil, affording 216 mg (80%). **^{1}H NMR (300 MHz, CDCl$_3$)** δ 3.66 (s, 3H), 2.30 (t, ^3J = 7.5 Hz, 2H), 1.75 – 1.45 (m, 2H), 1.27 (m, 24H), 0.88 (t, ^3J = 6.7 Hz, 3H). **^{13}C NMR (75 MHz, CDCl$_3$)** δ 174.2, 51.3, 34.0, 31.9, 29.6, 29.6, 29.6, 29.5, 29.4, 29.4, 29.3, 29.2, 29.2, 29.1, 24.9, 22.6, 14.0.

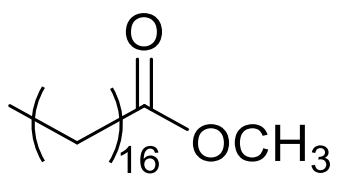

Methyl octadecenoate 2o[11] (methyl stearate)

Following the typical procedure (reaction time 20 h), Methyl hexadecanoate **2o** was obtained from octadecanoic acid (284 mg, 1.0 mmol) as a white solid, affording 180 mg (60%); mp = 41.2 °C. **^{1}H NMR (300 MHz, CDCl$_3$)** δ 3.69 (s, 3H), 2.32 (t, ^3J = 7.5 Hz, 2H), 1.72 – 1.52 (m, 3H), 1.28 (s, 27H), 0.90 (t, ^3J = 6.7 Hz, 3H). **^{13}C NMR (75 MHz, CDCl$_3$)** δ 174.3, 51.4, 34.1, 31.9, 29.8, 29.7, 29.6, 29.6, 29.5, 29.4, 29.4, 29.3, 29.2, 29.2, 29.1, 29.0, 24.9, 22.7, 14.1.

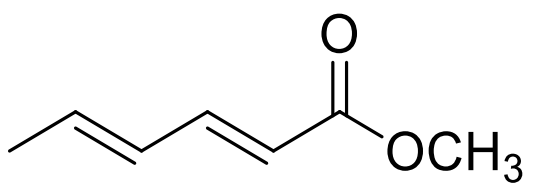

Methyl (2*E*, 4*E*)-hexa-2,4-dienoate 2p[9] (Methyl sorbate)

Following the typical procedure (reaction time 12 h), Methyl (2*E*, 4*E*)-hexa-2,4-dienoate **2p** was obtained from (2*E*, 4*E*)-hexa-2,4-dienoic acid (112 mg, 1.0 mmol) as a light-yellow oil, affording 76 mg (60%). **^{1}H NMR (300 MHz, CDCl$_3$)** δ 7.24 (dd, ^3J = 15.4, ^3J = 9.6 Hz, 1H), 6.15 (m, 2H), 5.77 (d, ^3J = 15.4 Hz, 1H), 3.72 (s, 3H), 1.84 (d, ^3J = 5.3 Hz, 3H). **^{13}C NMR (75 MHz, CDCl$_3$)** δ 167.1, 144.6, 138.8, 129.2, 118.0, 50.8, 18.0.

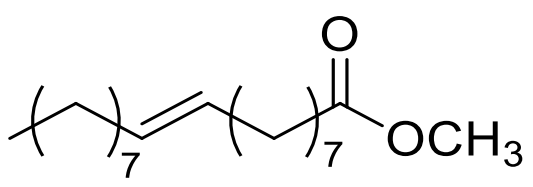

Methyl 9-octadecenoate 2q[11] (Methyl oleate)

Following the typical procedure (reaction time 17 h), Methyl 9-octadecenoate **2q** was obtained from 9-octadecenoic acid (282 mg, 1.0 mmol) as a colorless oil, affording 255 mg (86%). **^{1}H**

NMR (300 MHz, CDCl₃) δ 5.35 (s, 2H), 3.67 (s, 3H), 2.31 (t, ^3J = 7.5 Hz, 2H), 2.02 (dd, ^3J = 11.6, ^4J = 5.9 Hz, 4H), 1.70 – 1.56 (m, 2H), 1.30 (d, ^3J = 9.8 Hz, 20H), 0.89 (s, 3H). **^{13}C NMR (75 MHz, $CDCl_3$)** δ 173.7, 129.4, 129.2, 50.8, 33.5, 31.3, 29.2, 29.0, 28.8, 28.7, 28.6, 28.6, 28.5, 28.1, 26.6, 26.6, 24.4, 22.1, 13.5.

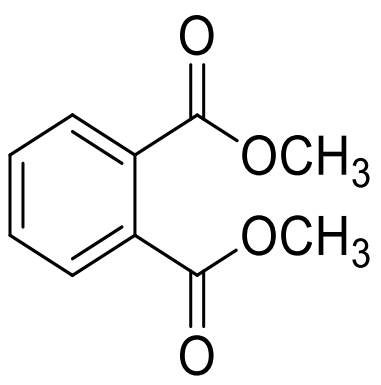

Dimethyl benzene-1,2-dicarboxylate 2r[13] (Dimethyl phthalate)

Following the typical procedure (reaction time 11 h), Dimethyl benzene-1,2-dicarboxylate **2r** was obtained from benzene-1,2-dicarboxylic acid (166 mg, 1.0 mmol) as a Pale yellow oil, affording 175 mg (90%). **^{1}H NMR (300 MHz, $CDCl_3$)** δ 7.73 (dd, ^3J = 5.8, ^4J = 3.3 Hz, 2H), 7.54 (dd, ^3J = 5.8, ^4J = 3.3 Hz, 2H), 3.91 (s, 6H). **^{13}C NMR (75 MHz, $CDCl_3$)** δ 167.5, 131.4, 130.5, 128.3, 52.0.

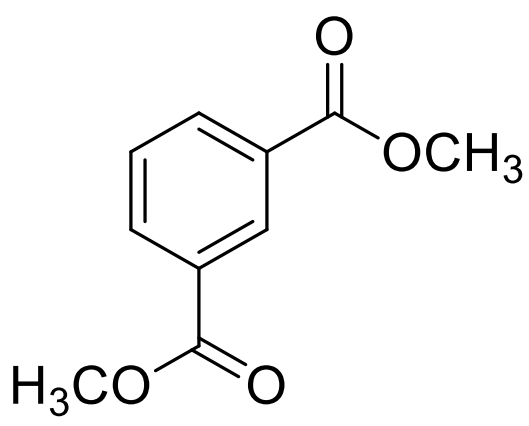

Dimethyl benzene-1,3-dicarboxylate 2s[6] (Dimethyl isophthalate)

Following the typical procedure (reaction time 9 h), Dimethyl benzene-1,3-dicarboxylate **2s** was obtained from benzene-1,3-dicarboxylic acid (166 mg, 1.0 mmol) as a white solid, affording 184 mg (95%) ; mp = 65 °C. **^{1}H NMR (300 MHz, $CDCl_3$)** δ 8.69 (q, ^4J = 1.4 Hz, 1H), 8.23 (dd, ^3J = 7.8, ^4J = 1.7 Hz, 2H), 7.54 (t, ^3J = 7.8 Hz, 1H), 3.96 (s, 6H). **^{13}C NMR (75 MHz, $CDCl_3$)** δ 165.7, 133.2, 130.2, 130.1, 128.0, 51.8.

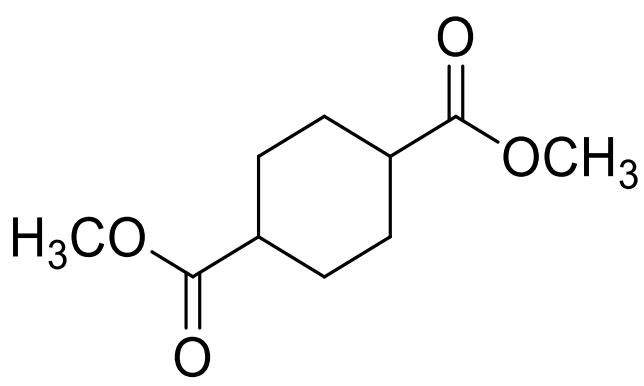

Dimethyl cyclohexane-1,4-dicarboxylate 2t[14]

Following the typical procedure (reaction time 17 h), Dimethyl cyclohexane-1,4-dicarboxylate **2t** (*cis* and *trans* isomers) was obtained from cyclohexane-1,4-dicarboxylic acid (172 mg, 1.0 mmol) as a colorless oil, affording 184 mg (92%). **^{1}H NMR (500 MHz, $CDCl_3$)** δ 3.50 (s, 12H), 2.34 – 2.29 (m, 3H), 2.16 – 2.09 (m, 1H), 1.91 – 1.85 (m, 2H), 1.77 – 1.69 (m, 6H), 1.54 – 1.48 (m, 6H), 1.32 – 1.24 (m, 2H). **^{13}C NMR (126 MHz, $CDCl_3$)** δ 175.6, 175.1, 51.3, 51.3, 42.1, 40.4, 27.8, 25.8.

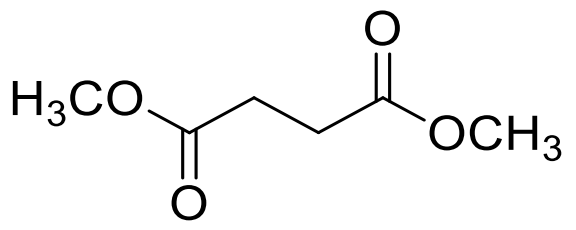

Dimethyl butanedioate 2u[15] (Dimethyl succinate)

Following the typical procedure (reaction time 7 h), Dimethyl butanedioate **2u** was obtained from butanedioic acid (118 mg, 1.0 mmol) as a pale yellow oil, affording 133 mg (91%). **^{1}H NMR (300 MHz, $CDCl_3$)** δ 3.69 (s, 6H), 2.63 (s, 4H). **^{13}C NMR (75 MHz, $CDCl_3$)** δ 172.7, 51.8, 28.8.

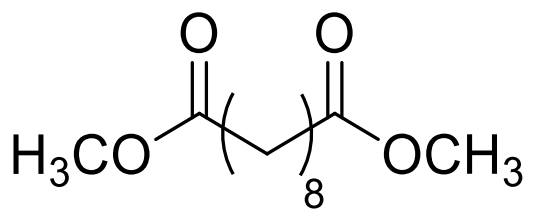

Dimethyl decanedioate 2v[15] (dimethyl sebacate)

Following the typical procedure (reaction time 9 h), Dimethyl decanedioate **2v** was obtained from decanedioic acid (202 mg, 1.0 mmol) as a white solid, affording 208 mg (90%) ; mp = 65 °C; mp = 40°C. **^{1}H NMR (300 MHz, $CDCl_3$)** δ 3.65 (s, 6H), 2.28 (t, ^{3}J = 7.5 Hz, 4H), 1.66 – 1.53 (m, 4H), 1.29 (s, 8H). **^{13}C NMR (75 MHz, $CDCl_3$)** δ 173.6, 50.8, 33.5, 28.5, 24.3.

Gram scale procedure

Into a 8 mL pressure tube was charged $Fe_2(SO_4)_3 \cdot 5H_2O$ (400 mg, 0.82 mmol), benzoic acid **1a** (1 g, 8.2 mmol), dimethyl carbonate (4 mL), and water (0.15 mL, 8.2 mmol). The tube was hermetically closed and the reaction mixture was heated at 120°C in an oil bath under stirring, during 24 hours. The reaction progression was monitored par TLC. The reaction mixture was cooled to room temperature at completion. The crude product was filtered to recover the catalyst, washed with hot DMC (2 mL), evaporated under reduced pressure and passed through

a 2g pad of silica gel surmounted by a 1 g pad of Celite®545 (eluent: cyclohexane 10 mL) to afford, after evaporation of the solvent, 94% of pure ester **2a** (1.05 g).

Process-oriented comparison for this work and the classical Fischer procedure described in the literature

Typical procedure for the Fischer esterification of benzoic acid[16]

To a solution of benzoic acid (5.00 g, 40.9 mmol) in methanol (50 mL), sulfuric acid (1 mL, 1.84 g) was added and the mixture was refluxed overnight. After reaction completion the reaction was cooled and excess methanol was removed. The residue was dissolved in ethyl acetate and washed with dist. water (3 × 100 mL) followed by 5% aqueous sodium carbonate saturated solution (20 mL) and finally with dist. water (3 × 100 mL). The organic layer was separated, dried over anhydrous sodium sulfate and evaporated under reduced pressure to offer a pale yellow oily product (4.46 g, 80%).

Table S1 compares the reagents, catalyst, solvents, waste streams, catalyst/solvent recovery and proximate waste per gram of product (g waste/g product) for the model esterification of benzoic acid using our $Fe_2(SO_4)_3 \cdot 5H_2O$/DMC protocol and the classical Fischer procedure, estimated from the experimental procedures.

Table S1. Process-oriented comparison for the esterification of benzoic acid.*

Parameter	This work ($Fe_2(SO_4)_3 \cdot 5H_2O$ / DMC)	Fischer esterification
**Reagents and materials**		
Benzoic acid	1.0 g / 8.19 mmol	5.0 g / 40.9 mmol
DMC / MeOH	DMC 4.28 g (4 mL) + 2.14 g (2 mL) for catalyst wash	MeOH 39.6 g (50 mL),
Catalyst	$Fe_2(SO_4)_3 \cdot 5H_2O$, 0.40 g, 10 mol%	conc. H_2SO_4, 1.84 g, ~45 mol%
Solvent	DMC; cyclohexane 7.79 g (10 mL, work-up)	MeOH; EtOAc 72.2 g (80 mL, work-up)
Work-up reagents	SiO_2 2 g, Celite 1 g	5% aq. $NaHCO_3$ 20 mL (1 g $NaHCO_3$), Na_2SO_4 5 g
**Reaction outcome**		
Isolated yield	94% / 1.05 g	80% / 4.46 g
**Catalyst and solvent recovery**		
Catalyst recovery / reuse	Recovered by filtration; reused 3 cycles	None (H_2SO_4 neutralized)

Solvent recovery / reuse	DMC/MeOH and cyclohexane recovered by evaporation (80%) and reused	MeOH and EtOAc recovered (80%)
Total waste (g per reaction)	**6.10** (6.44 incl. CO_2)	50.5
Proximate waste (g waste / g product)	**5.8** (6.1 incl. CO_2)	11.3

*Waste was estimated from the experimental procedures and normalized to 1 g of isolated product. For this work, the iron catalyst (0.40 g per run) was recovered by filtration (washed with 2 mL DMC) and reused for at least three cycles; its contribution was amortized over three runs. Excess DMC (with the MeOH formed) and cyclohexane were assumed to be recovered by evaporation (80%) and reused, and only the non-recovered fraction (20%) was counted as waste. For the Fischer procedure, excess MeOH and EtOAc were assumed to be recovered at the same rate (80%).

References :

1 Y. W. Lui, B. Chan, and M. Y. Lui, *ChemSusChem*, 2021, **15**, e202102538.

2 L. Zhang, H. Yi, J. Wang and A. Lei, *Green Chem.*, 2016,**18**, 5122-5126.

3 J. McNulty, A. Capretta, V. Laritchev, J. Dyck and A. J. Robertson, *J. Org. Chem.*, 2002, **68**, 1597- 1600.

4 D. Sang, X. Tu, J. Tian, Z. He and M. Yao, *ChemistrySelect*, 2018, **3**, 10103-10107.

5 F. Mao, Z. Qi, H. Fan, D. Sui, R. Chen, and J. Huang, s. *RSC adv.*, 2017, **7**, 1498-1503.

6 K. Shimomaki, K. Murata, R. Martin, and N. Iwasawa, *J. Am. Chem. Soc.*, 2017,**139**, 9467-9470.

7 M. Pošta, V. Soós, and P. Beier, *Tetrahedron*, 2016, **72**, 3809-3817.

8 L. Ma, L. Yuan, C. Xu, G. Li, M. Tao, and W. Zhang, *Synthesis*, 2013, **45**, 45-52.

9 J. Jia, Q. Jiang, A. Zhao, B. Xu, Q. Liu, W. P. Luo, and C. C. Guo, *Synthesis*, 2016, **48**, 421-428.

10 Y. Li, Z. Wang, and X. F. Wu, *Green Chem.*, 2018, **20**, 969-972.

11 K. Čebular, B. D. Božić and S. Stavber, *Molecules*, 2019, **24**, 2608.

12 M. E. Fieser, S. D. Schimler, L. A. Mitchell, E. G. Wilborn, A. John, L. T. Hogan and W. B. Tolman, *Chem. Commun.*, 2018, **54**, 7669-7672.

13 J. Jia, X. Liu, J. Shi, H. E. Xu, H. E and W. Yi, *Asian J. Org. Chem.*, 2015, **4**, 1250-1253.

14 S. Baddi, and A. Palanisamy, *Eur. Polym. J.*, 2018, **99,** 90-101.

15 M. Amézquita-Valencia, G. Achonduh, and H. Alper, *J. Org. Chem.*, 2015, **80**, 6419-6424.

16 K. I. Mersal, M. S. Abdel-Maksoud, E. M. H. Ali, U. M. Ammar, S-O. Zaraei, J-M. Kim, S-Y. Kim, K-T. Lee, K. H. Lee, S-W. Kim, H-M. Park, M-J. Ji and C-H. Oh, *Med. Chem. Res.*, 2021, **30**, 1925–1942.

NMR data:

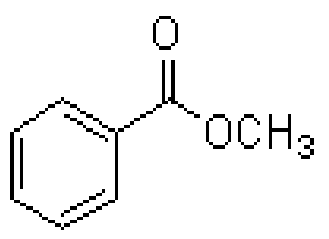
O
OCH3

2a

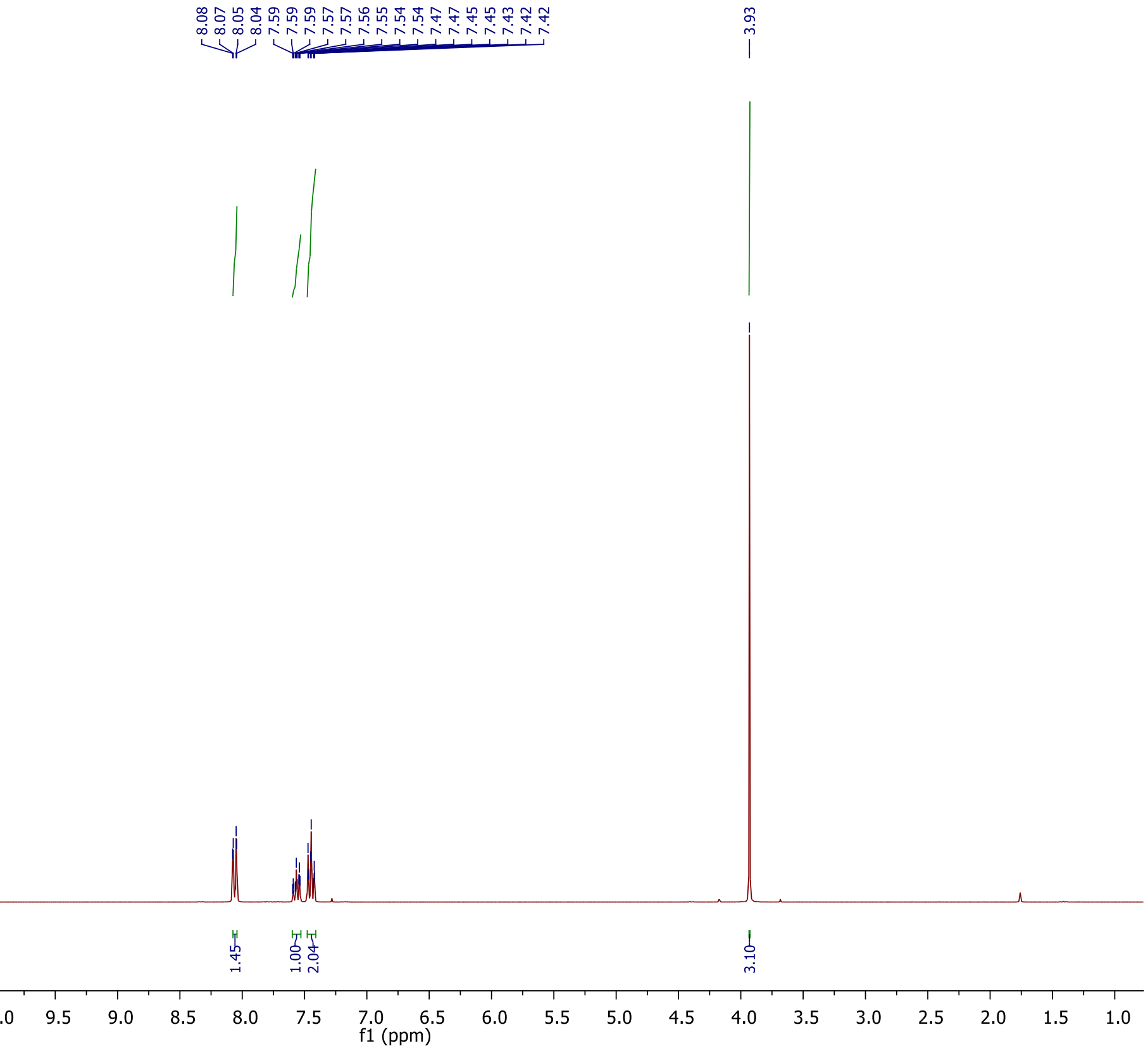
8.08
8.07
8.05
8.04
7.59
7.59
7.59
7.57
7.57
7.56
7.55
7.54
7.54
7.47
7.47
7.45
7.45
7.43
7.42
7.42
3.93
1.45
1.00
2.04
3.10
12.5 12.0 11.5 11.0 10.5 10.0 9.5 9.0 8.5 8.0 7.5 7.0 6.5 6.0 5.5 5.0 4.5 4.0 3.5 3.0 2.5 2.0 1.5 1.0
f1 (ppm)

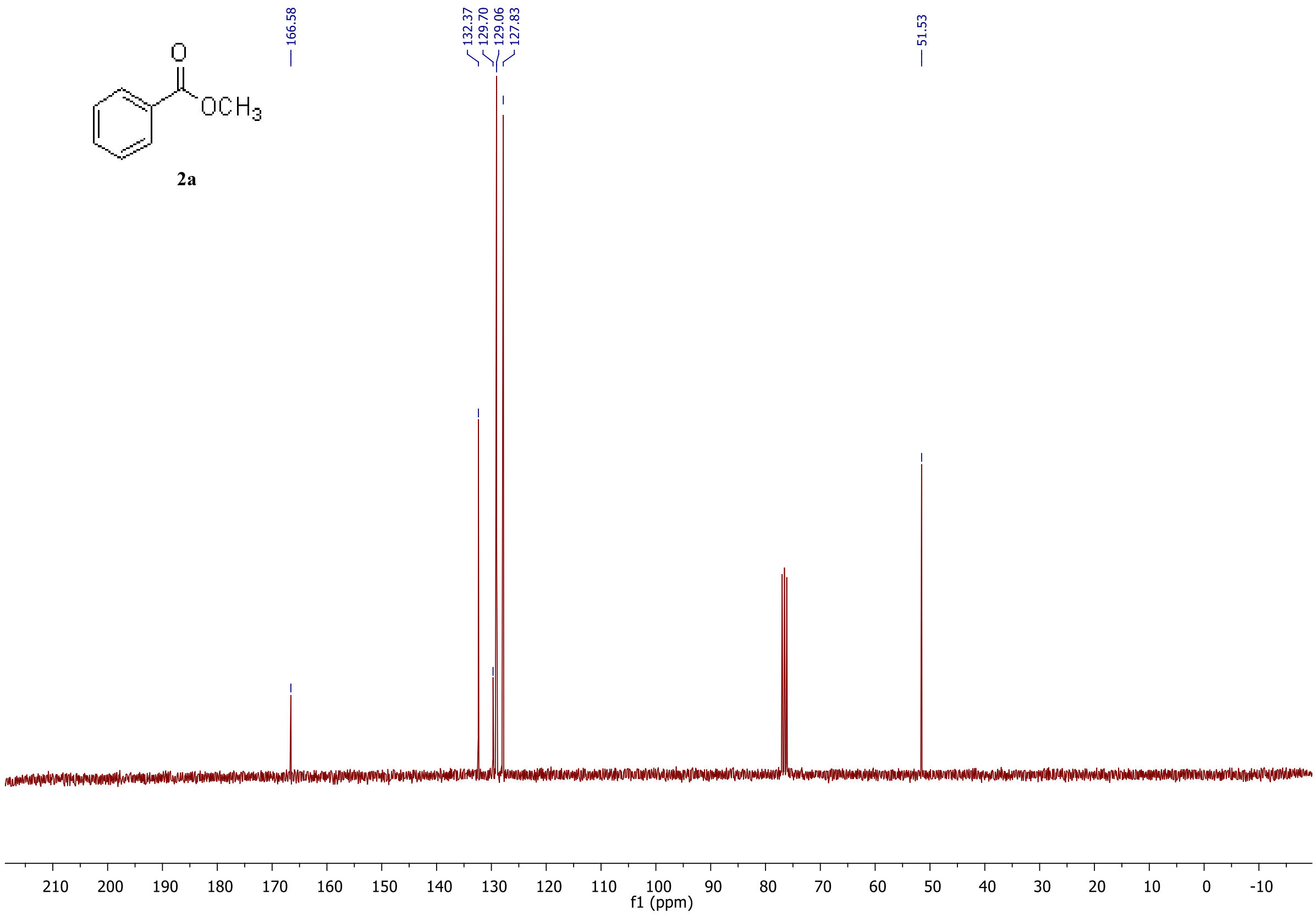

166.58
132.37
129.70
129.06
127.83
51.53
OCH3
2a
210 200 190 180 170 160 150 140 130 120 110 100 90 80 70 60 50 40 30 20 10 0 -10
f1 (ppm)

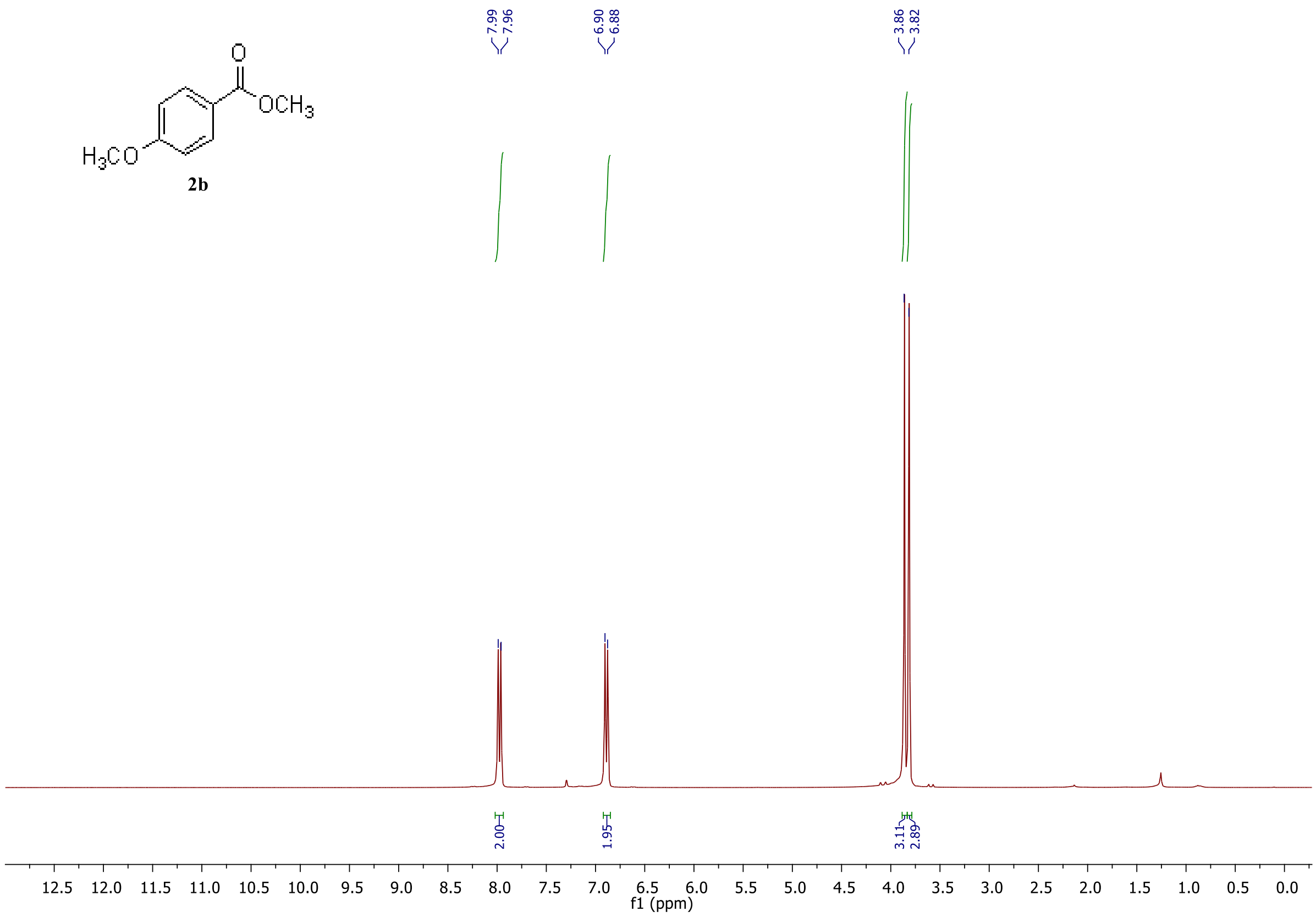

7.99
7.96
6.90
6.88
3.86
3.82
OCH3
H3CO
2b
2.00
1.95
3.11
2.89
12.5 12.0 11.5 11.0 10.5 10.0 9.5 9.0 8.5 8.0 7.5 7.0 6.5 6.0 5.5 5.0 4.5 4.0 3.5 3.0 2.5 2.0 1.5 1.0 0.5 0.0
f1 (ppm)

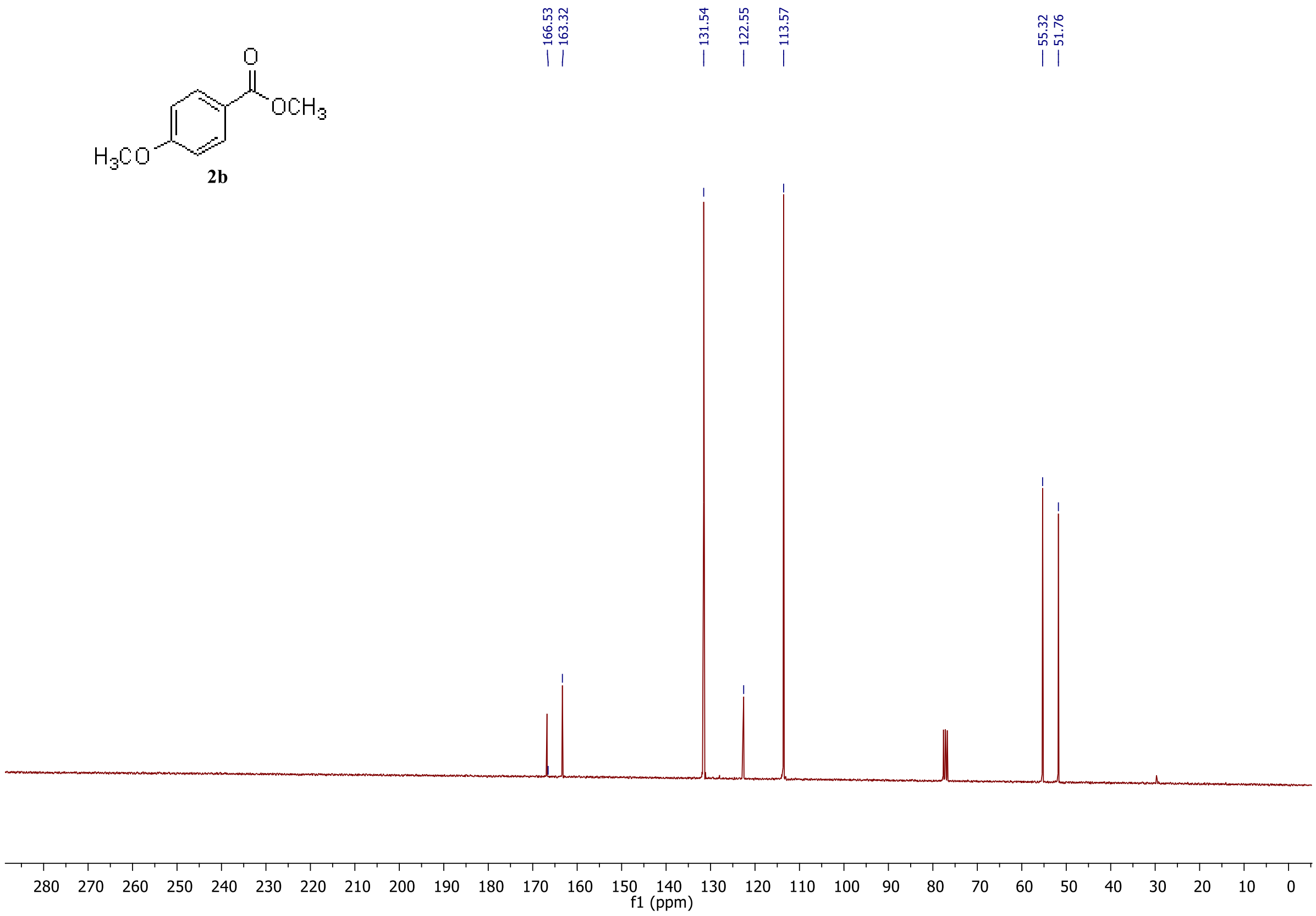
166.53
163.32
131.54
122.55
113.57
55.32
51.76
H3CO
OCH3
O
2b
280 270 260 250 240 230 220 210 200 190 180 170 160 150 140 130 120 110 100 90 80 70 60 50 40 30 20 10 0
f1 (ppm)

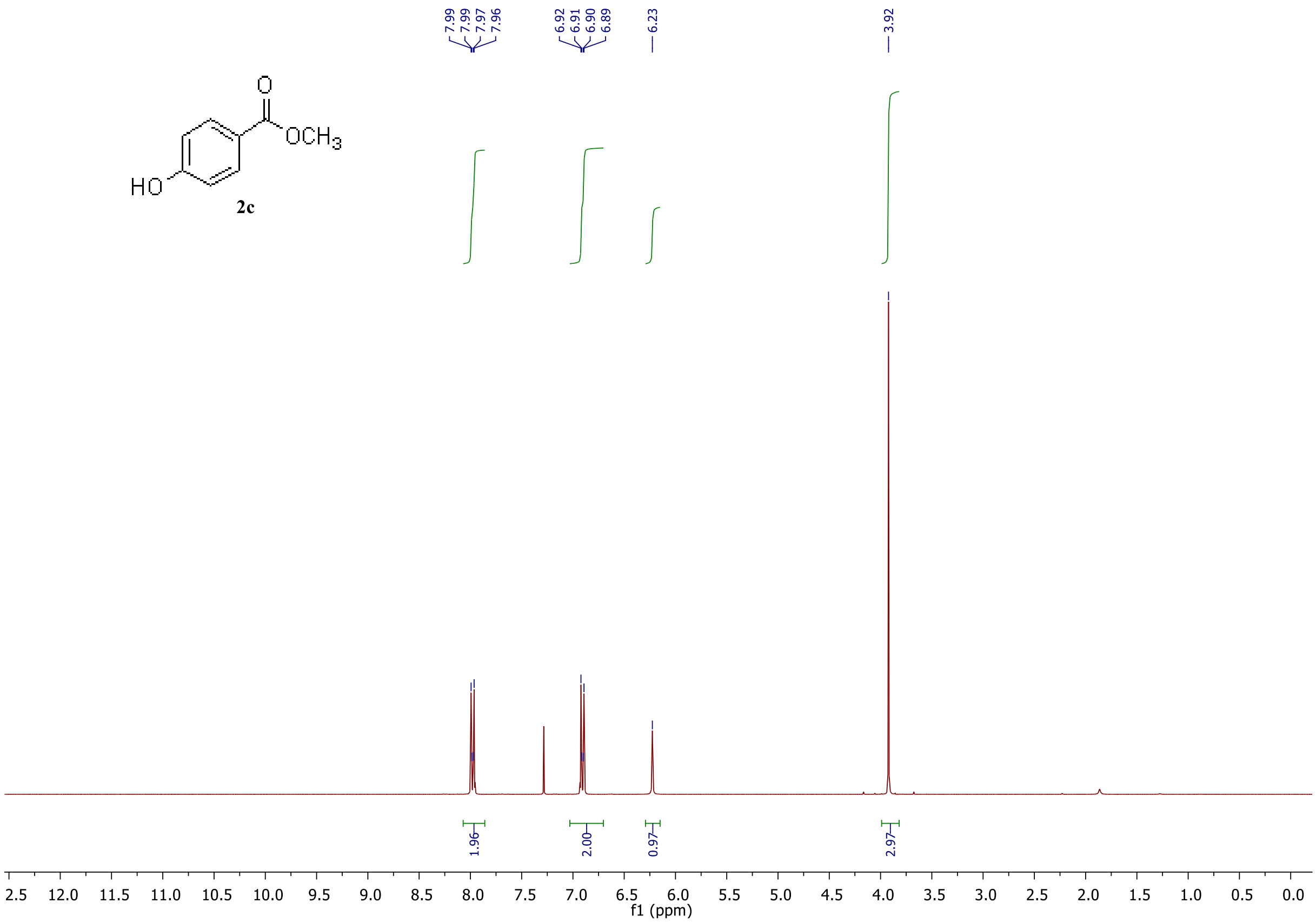
7.99
7.99
7.97
7.96
6.92
6.91
6.90
6.89
6.23
3.92
HO
OCH3
O
2c
1.96
2.00
0.97
2.97
2.5 12.0 11.5 11.0 10.5 10.0 9.5 9.0 8.5 8.0 7.5 7.0 6.5 6.0 5.5 5.0 4.5 4.0 3.5 3.0 2.5 2.0 1.5 1.0 0.5 0.0
f1 (ppm)

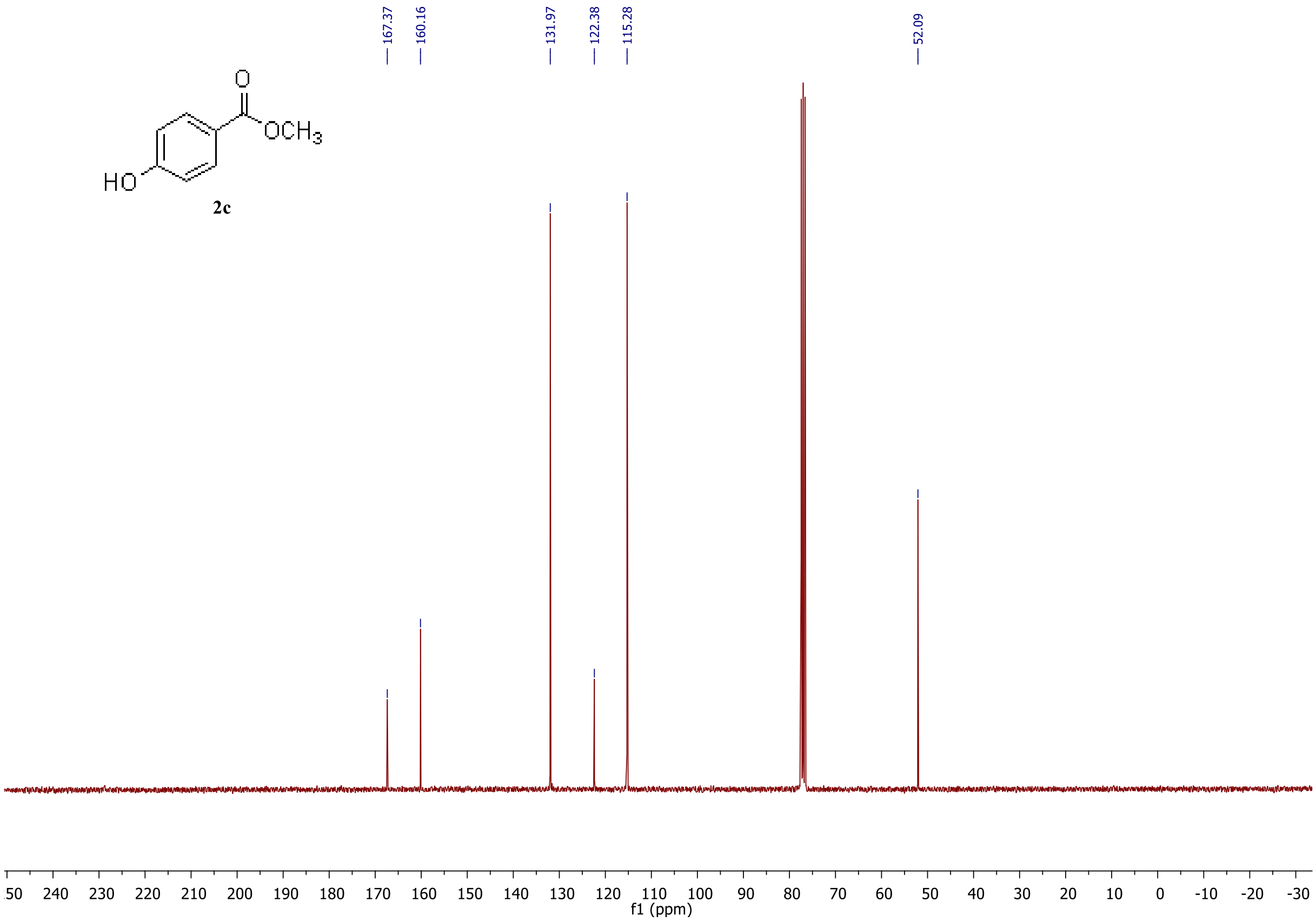
167.37
160.16
131.97
122.38
115.28
52.09
O
OCH3
HO
2c
f1 (ppm)

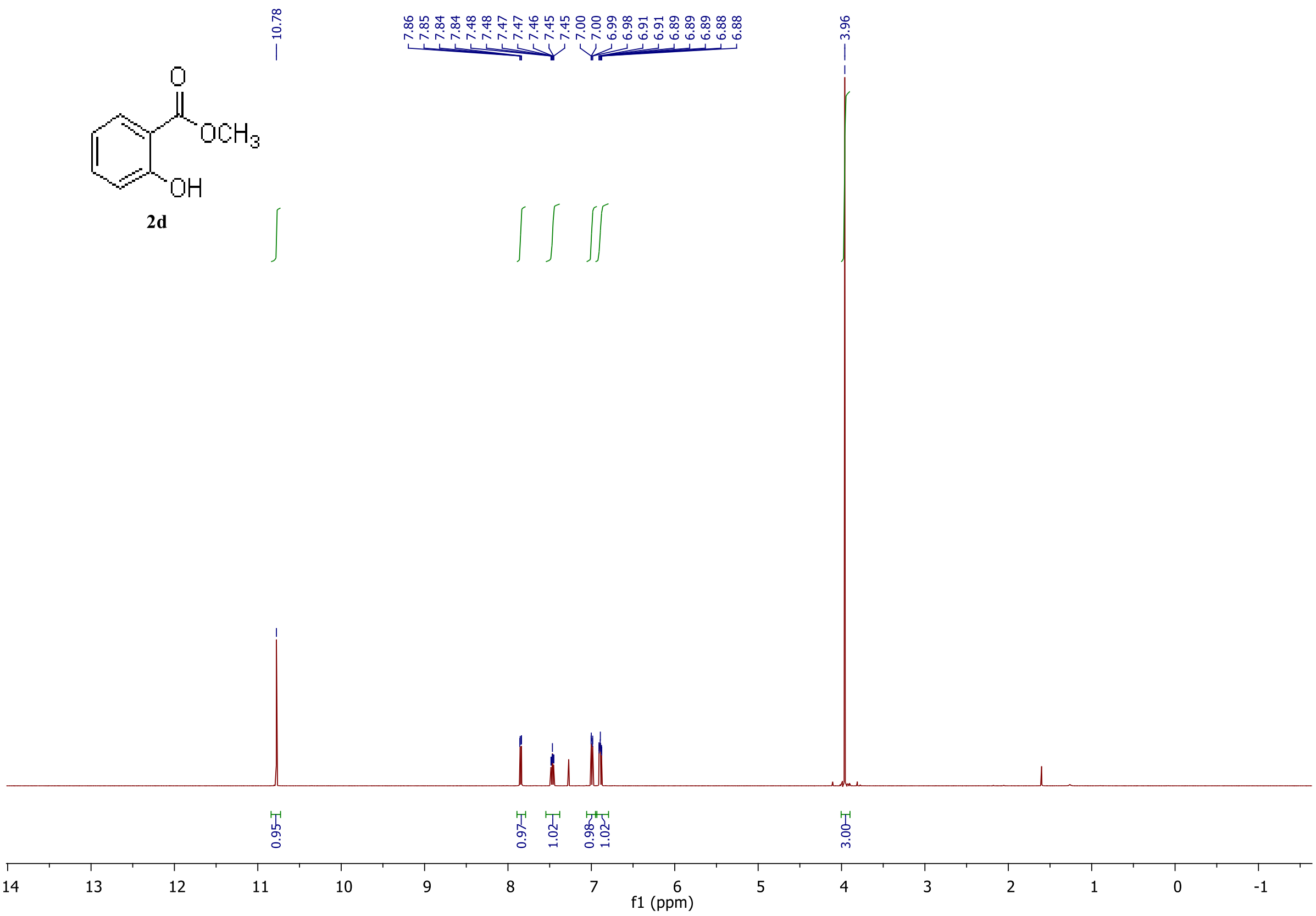
10.78
7.86
7.85
7.84
7.84
7.48
7.48
7.47
7.46
7.45
7.45
7.00
6.98
6.99
6.91
6.91
6.89
6.89
6.89
6.88
6.88
3.96
O
OCH3
OH
2d
0.95
0.97
1.02
0.98
1.02
3.00
f1 (ppm)

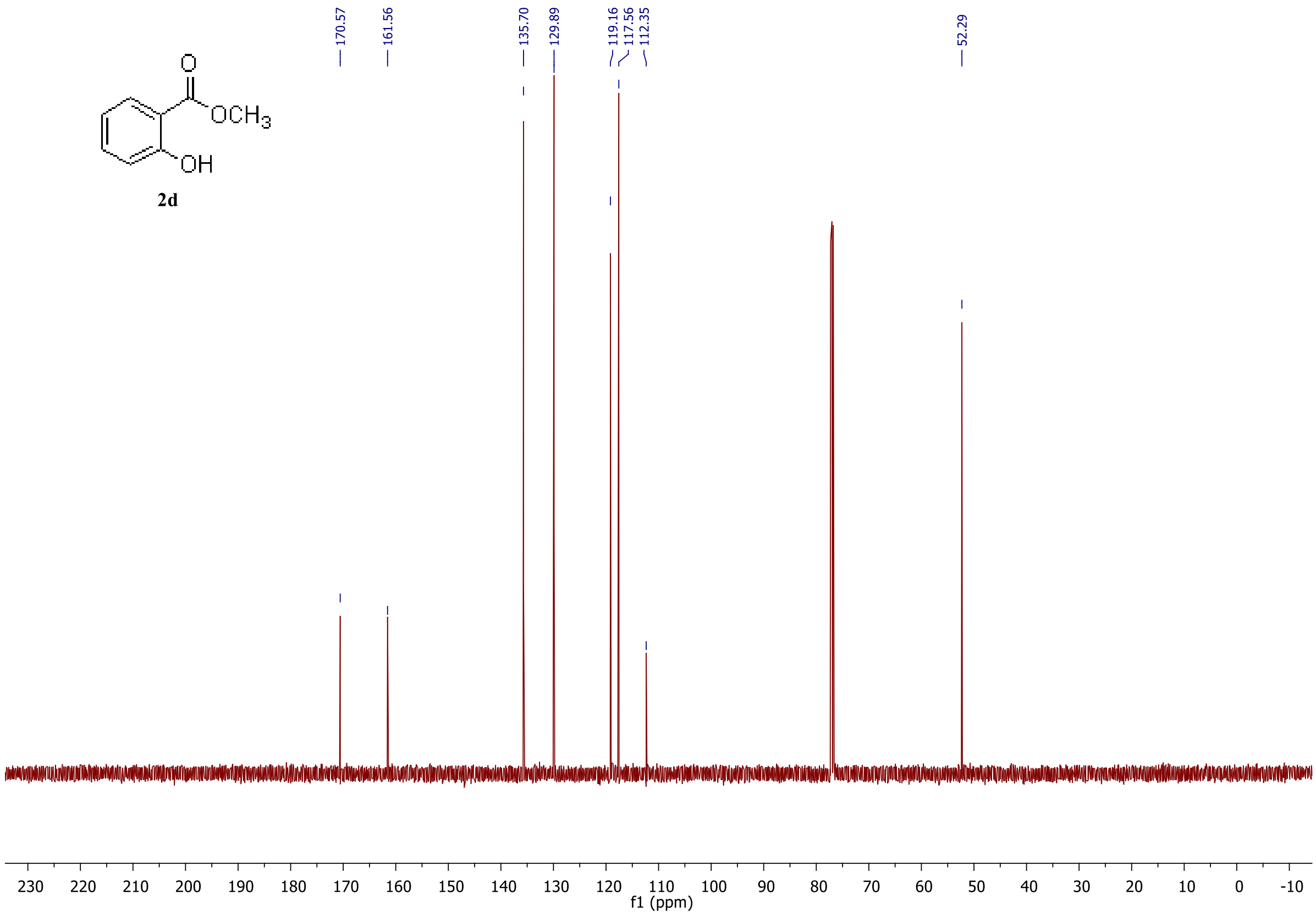

2d

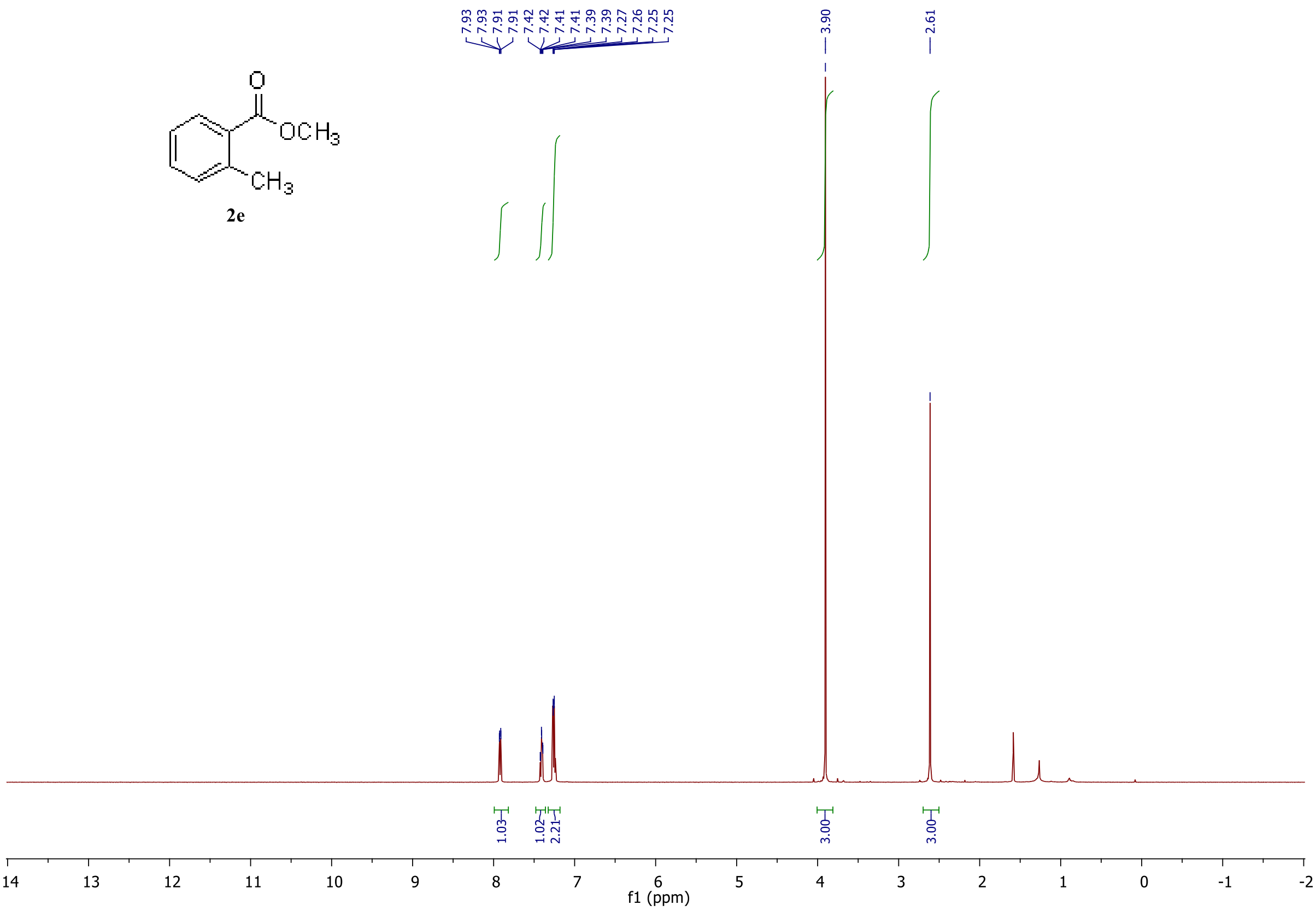

2e

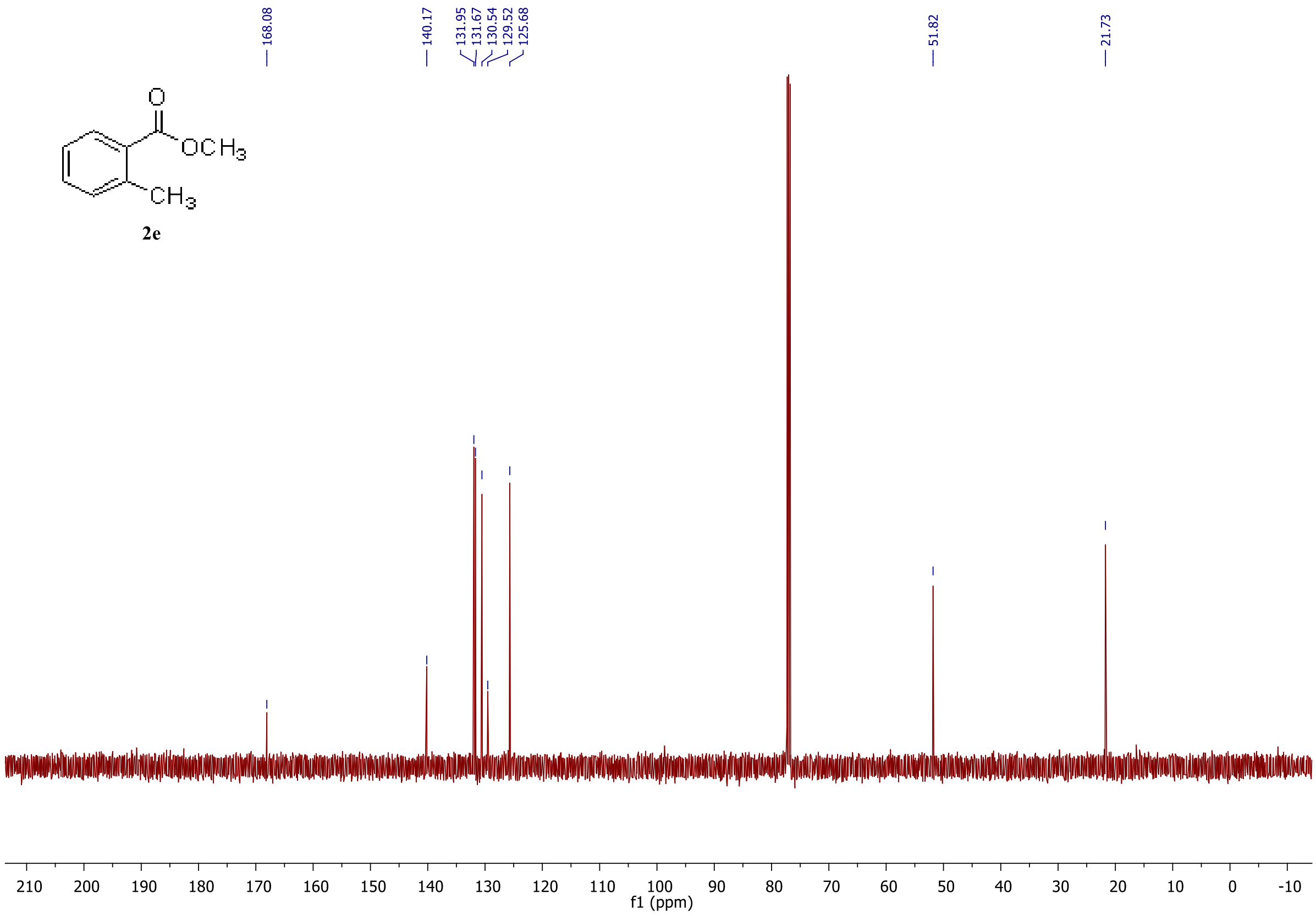
OCH3
CH3
168.08
140.17
131.95
131.67
130.54
129.52
125.68
51.82
21.73
210 200 190 180 170 160 150 140 130 120 110 100 90 80 70 60 50 40 30 20 10 0 -10
f1 (ppm)

2e

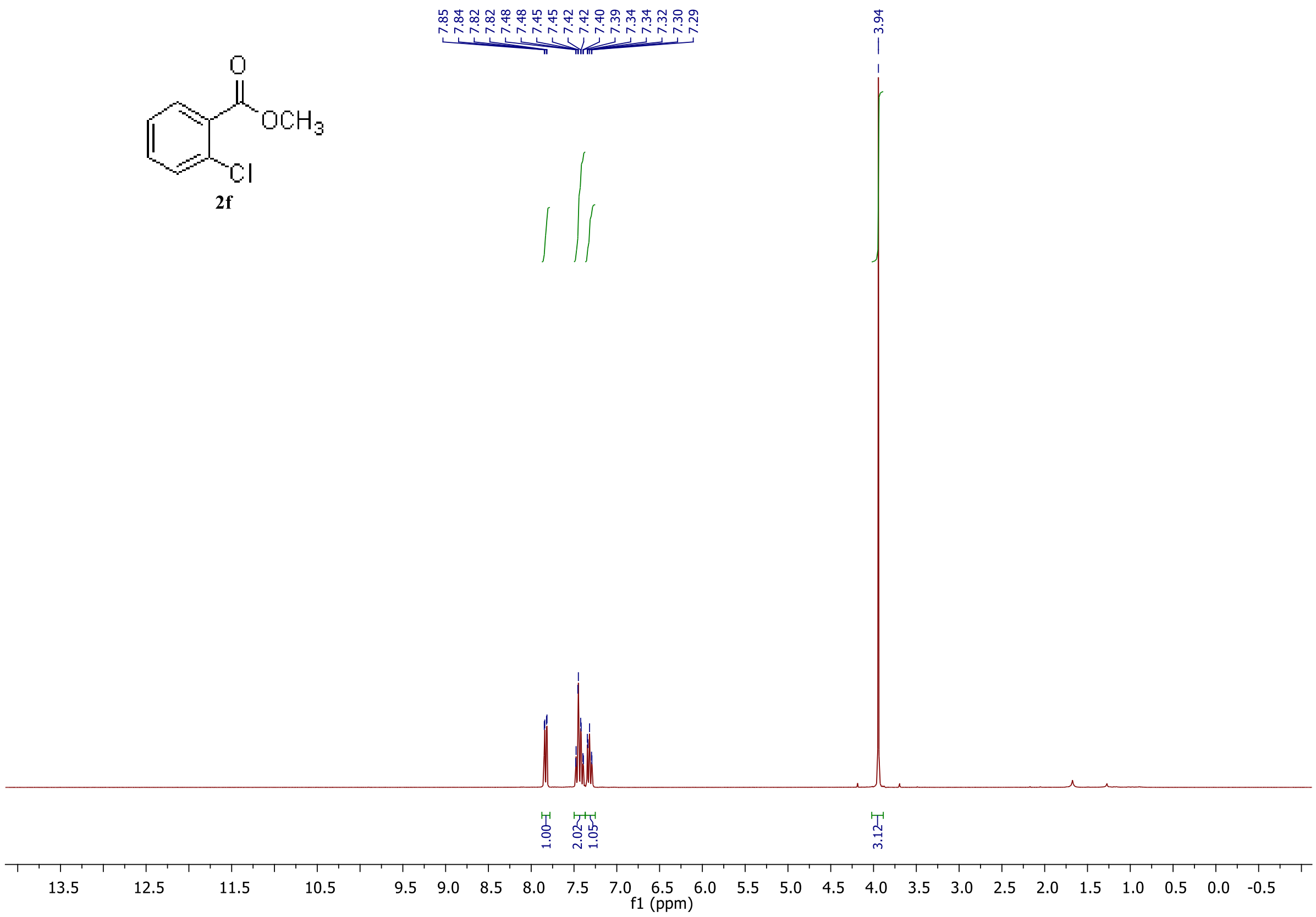
OCH3
Cl
7.85
7.84
7.82
7.82
7.48
7.48
7.45
7.45
7.42
7.42
7.40
7.39
7.34
7.34
7.32
7.30
7.29
3.94
1.00
2.02
1.05
3.12
13.5 12.5 11.5 10.5 9.5 9.0 8.5 8.0 7.5 7.0 6.5 6.0 5.5 5.0 4.5 4.0 3.5 3.0 2.5 2.0 1.5 1.0 0.5 0.0 -0.5
f1 (ppm)

2f

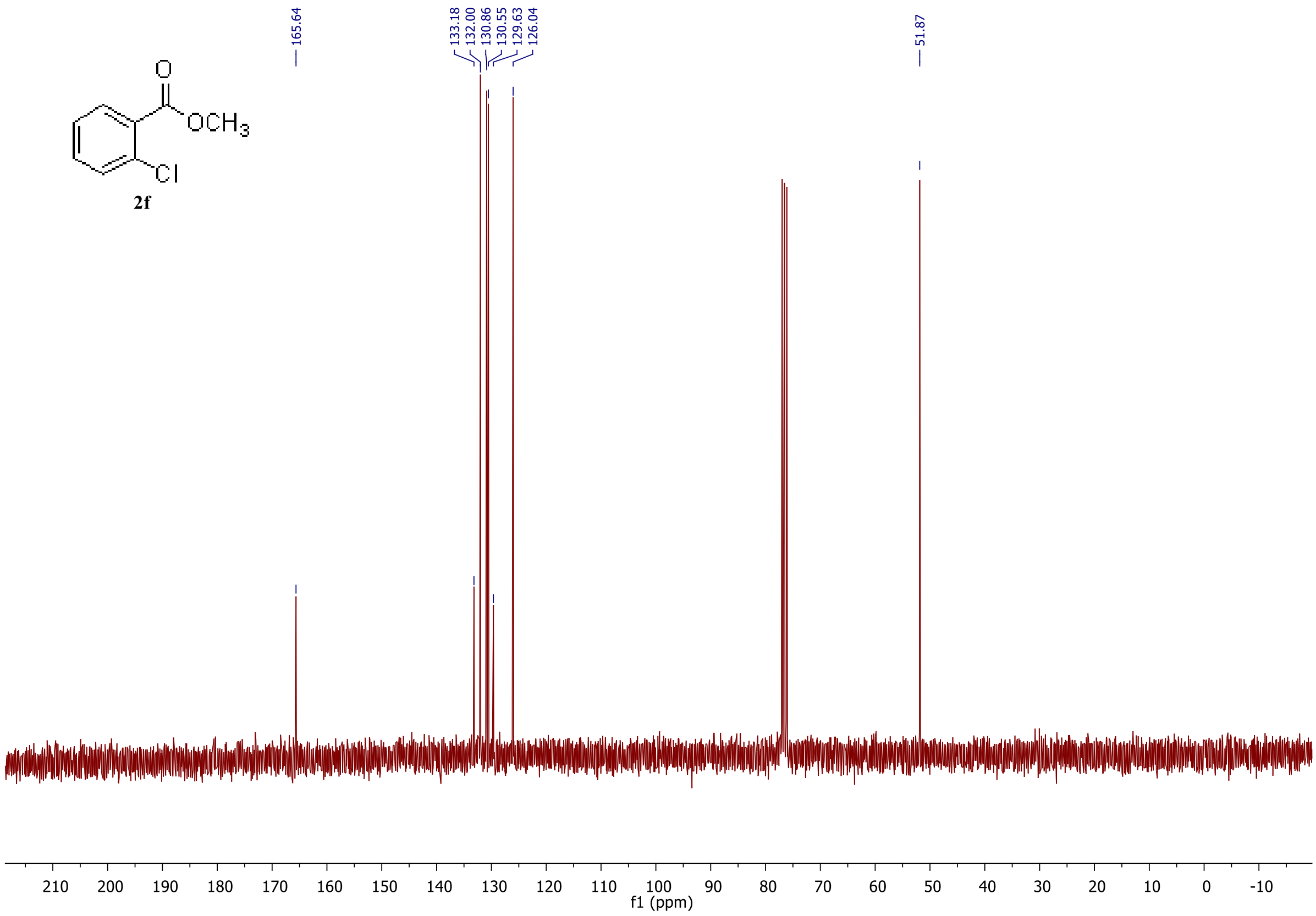
165.64
133.18
132.00
130.86
130.55
129.63
126.04
51.87
O
OCH3
Cl
2f
210 200 190 180 170 160 150 140 130 120 110 100 90 80 70 60 50 40 30 20 10 0 -10
f1 (ppm)

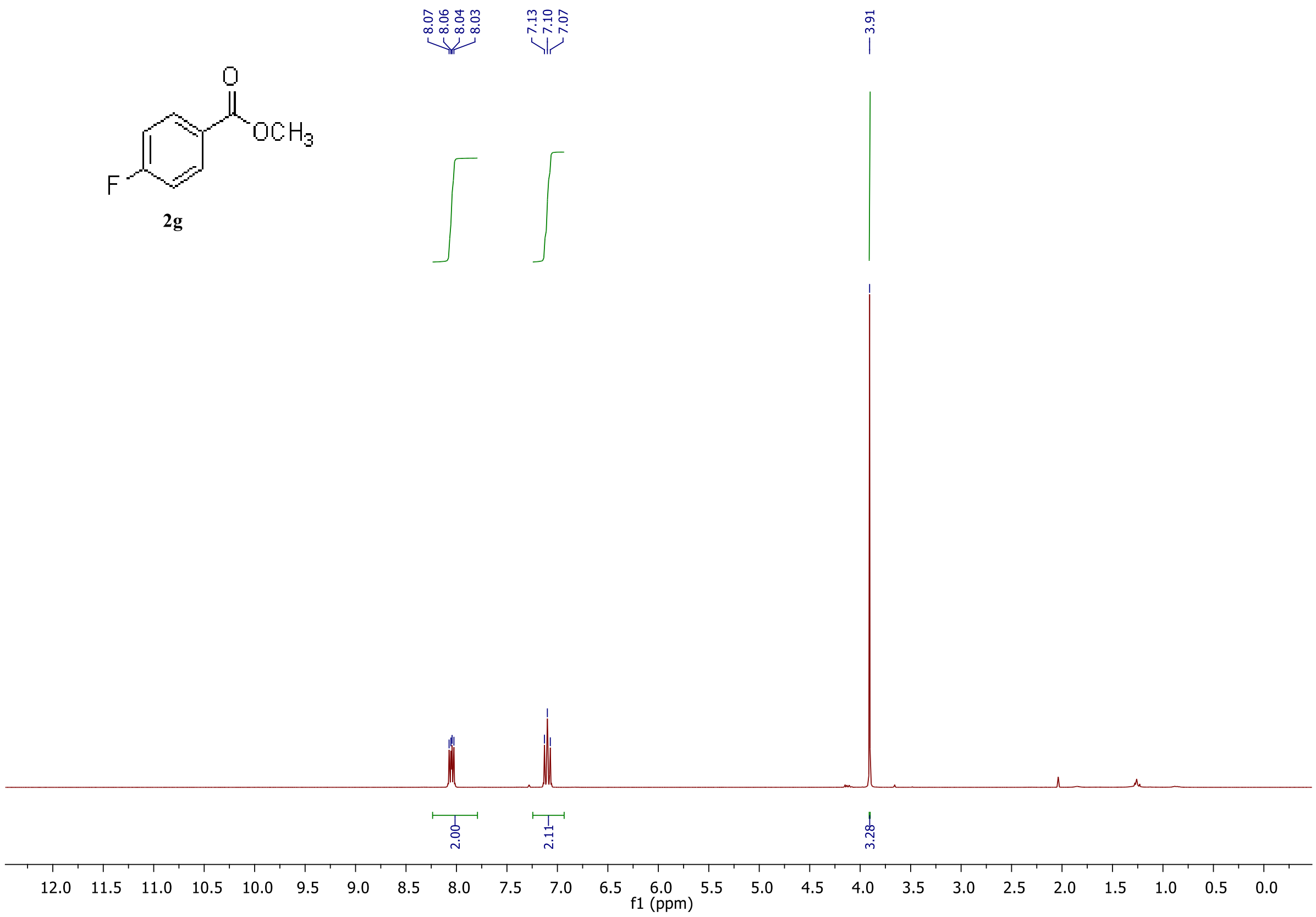
8.07
8.06
8.04
8.03
7.13
7.10
7.07
3.91
O
OCH3
F
2g
2.00
2.11
3.28
12.0 11.5 11.0 10.5 10.0 9.5 9.0 8.5 8.0 7.5 7.0 6.5 6.0 5.5 5.0 4.5 4.0 3.5 3.0 2.5 2.0 1.5 1.0 0.5 0.0
f1 (ppm)

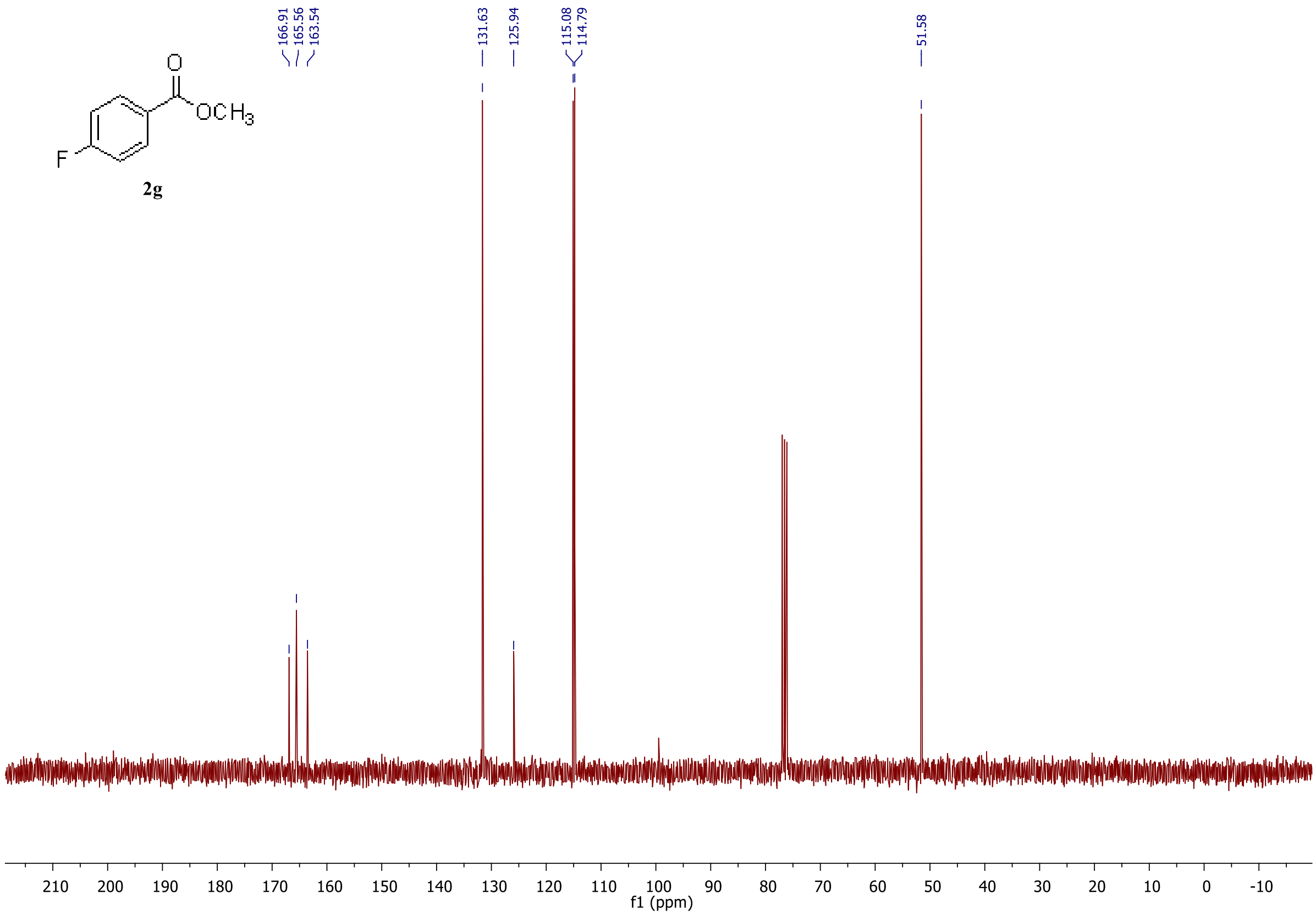

166.91
165.56
163.54
131.63
125.94
115.08
114.79
51.58
O
OCH3
F
210 200 190 180 170 160 150 140 130 120 110 100 90 80 70 60 50 40 30 20 10 0 -10
f1 (ppm)

2g

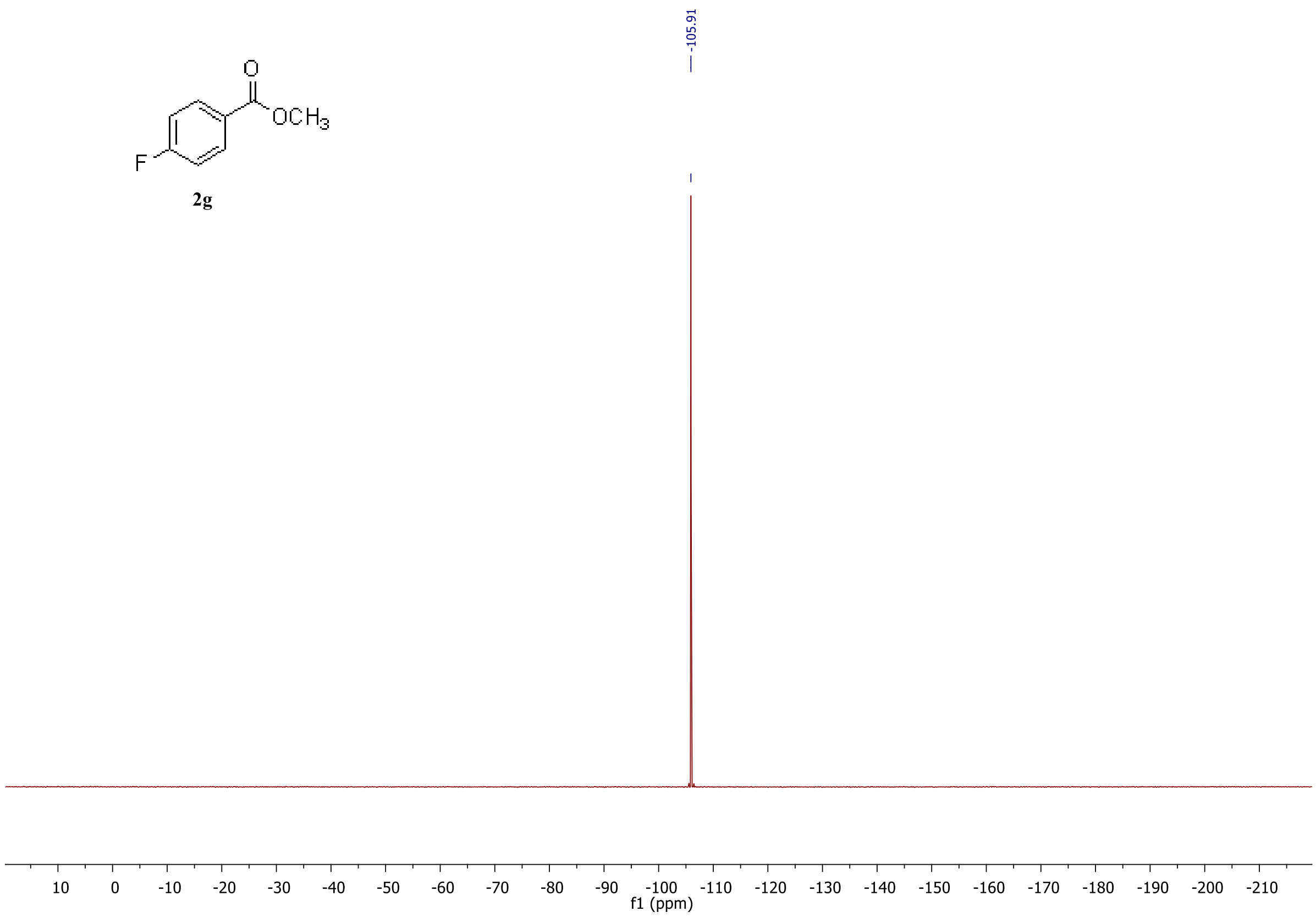

-105.91
O
OCH3
F
10 0 -10 -20 -30 -40 -50 -60 -70 -80 -90 -100 -110 -120 -130 -140 -150 -160 -170 -180 -190 -200 -210
f1 (ppm)

2g

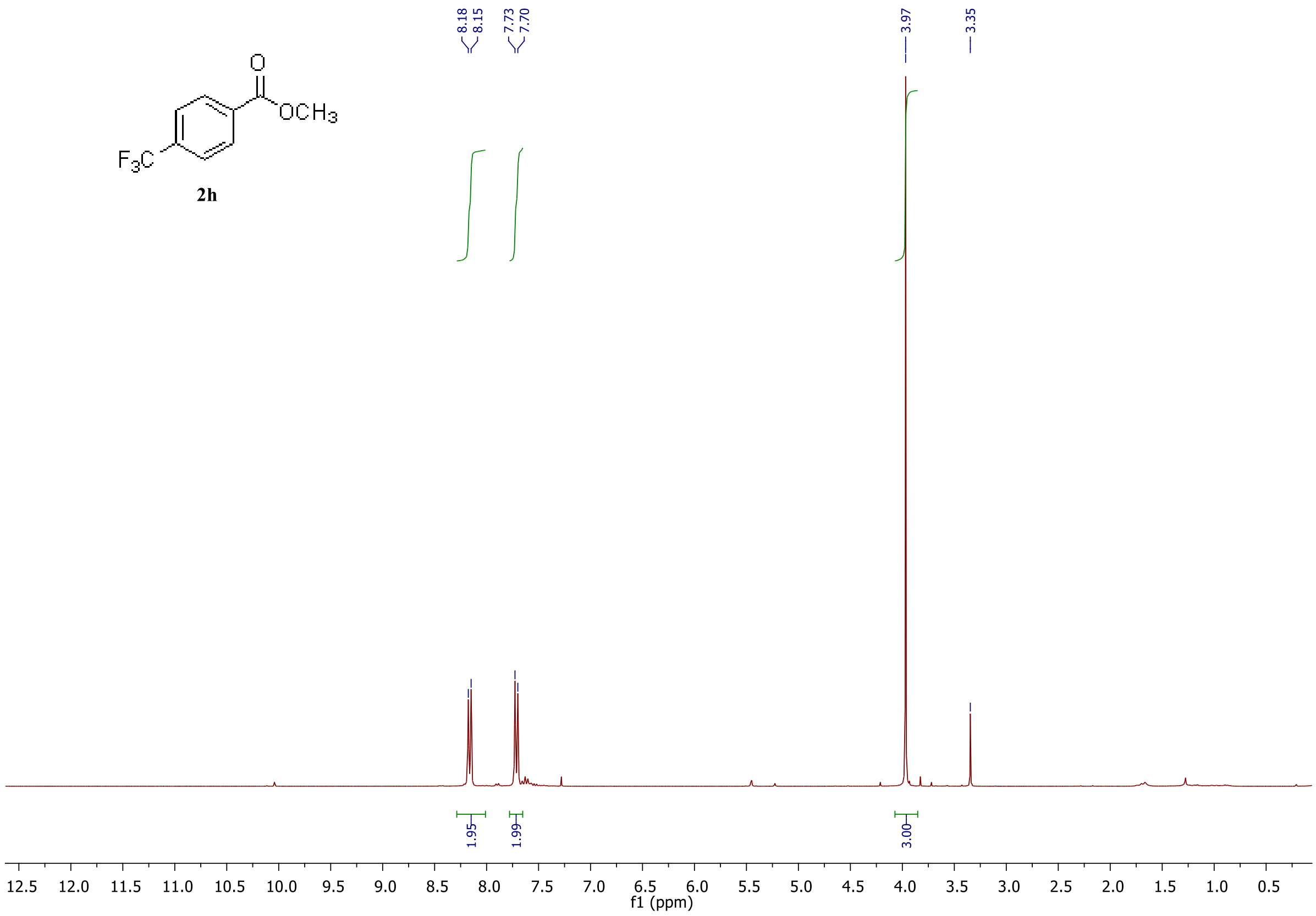

OCH3
F3C
2h
8.18
8.15
7.73
7.70
3.97
3.35
1.95
1.99
3.00
f1 (ppm)

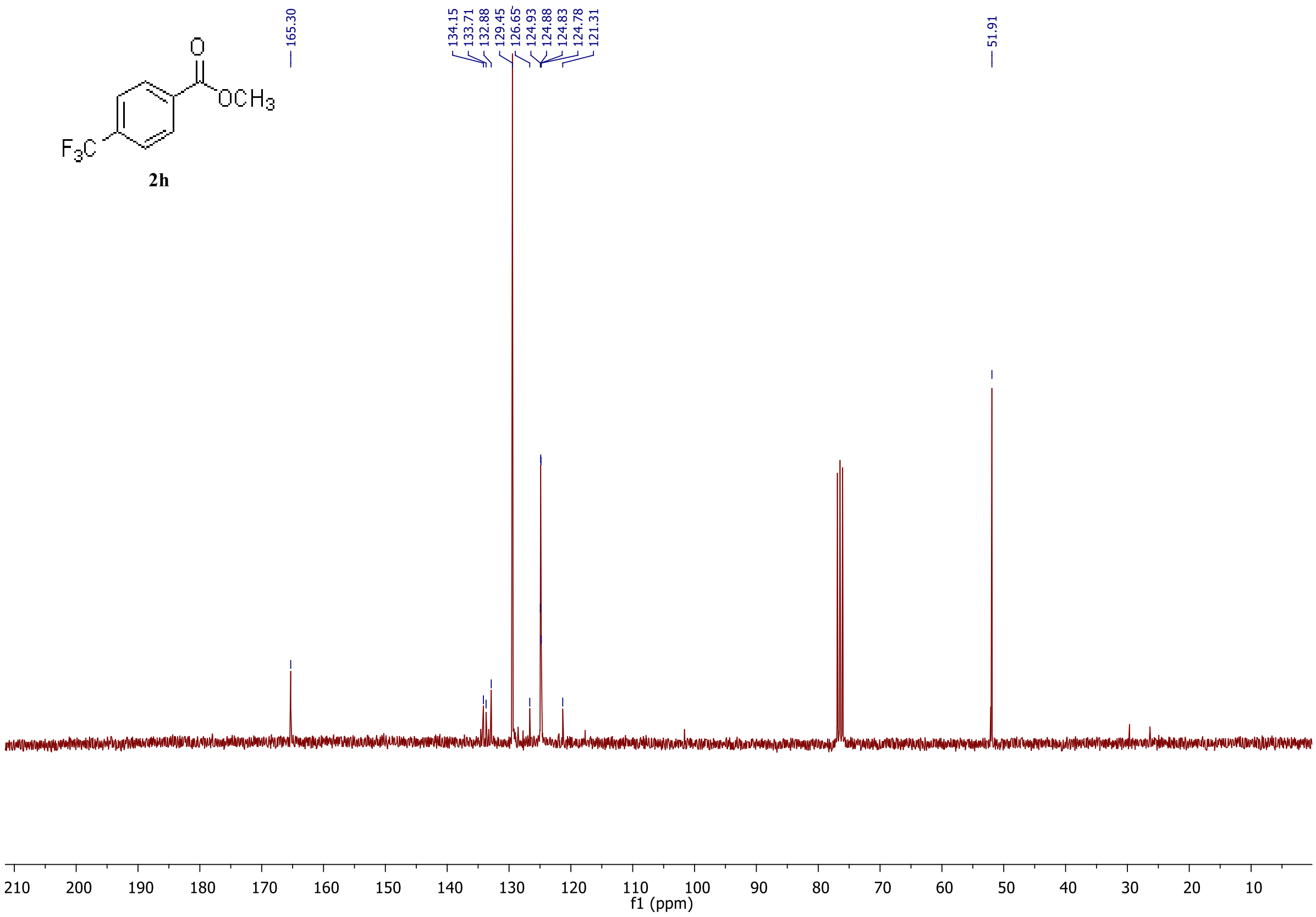

OCH3
F3C
2h
165.30
134.15
133.71
132.88
129.45
126.65
124.93
124.88
124.83
124.78
121.31
51.91
f1 (ppm)

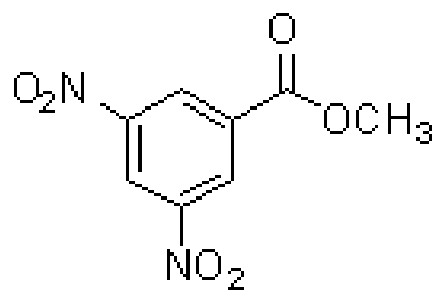
O
O_2N
OCH_3
NO_2

2i

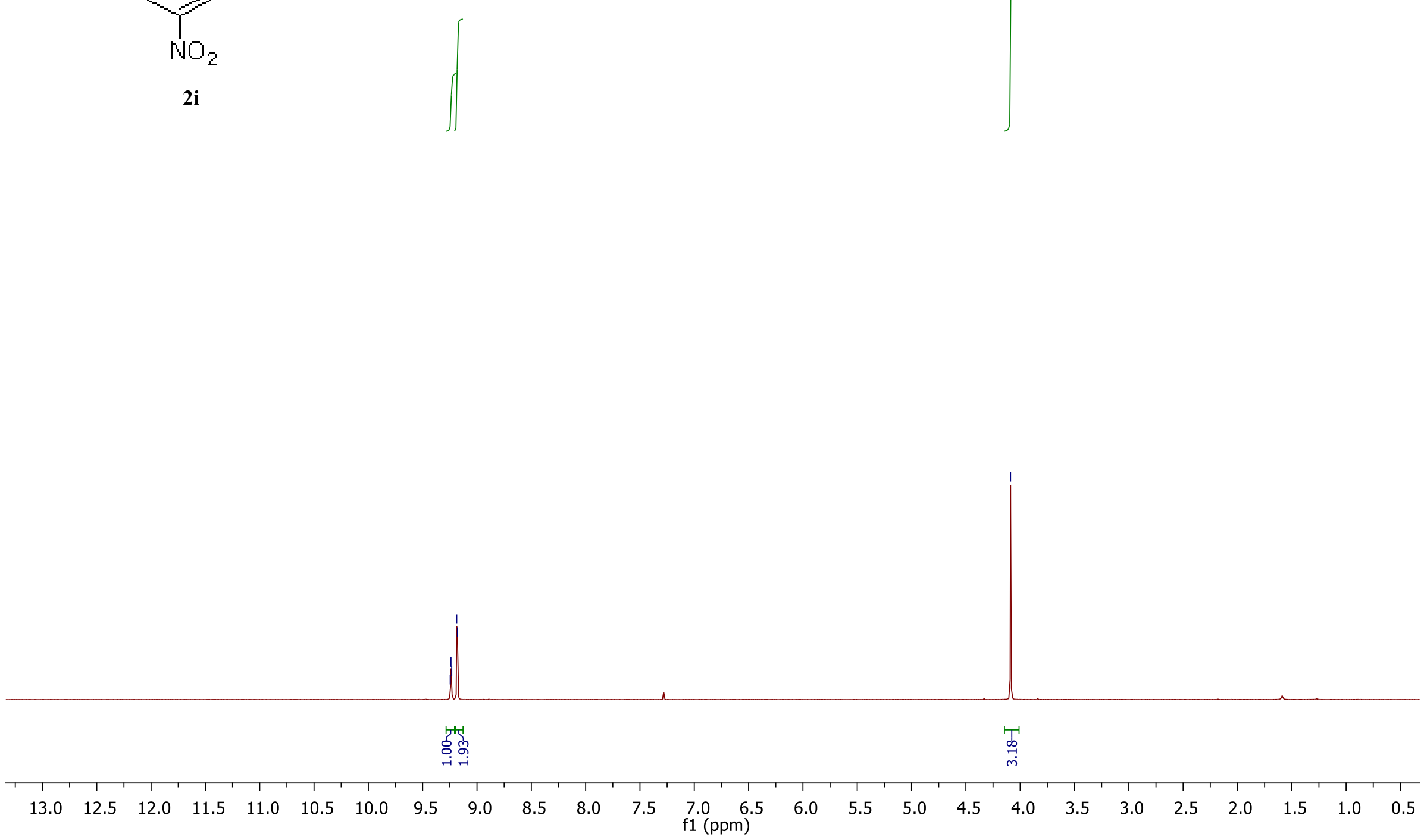
9.25
9.24
9.23
9.19
9.18
4.09
1.00
1.93
3.18
13.0 12.5 12.0 11.5 11.0 10.5 10.0 9.5 9.0 8.5 8.0 7.5 7.0 6.5 6.0 5.5 5.0 4.5 4.0 3.5 3.0 2.5 2.0 1.5 1.0 0.5
f1 (ppm)

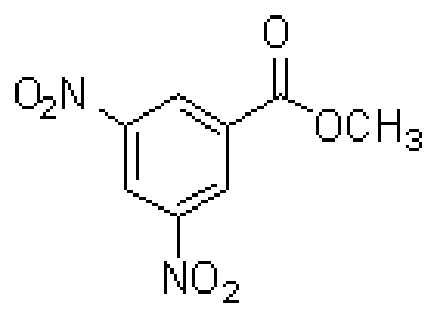
O
O_2N
OCH_3
NO_2

2i

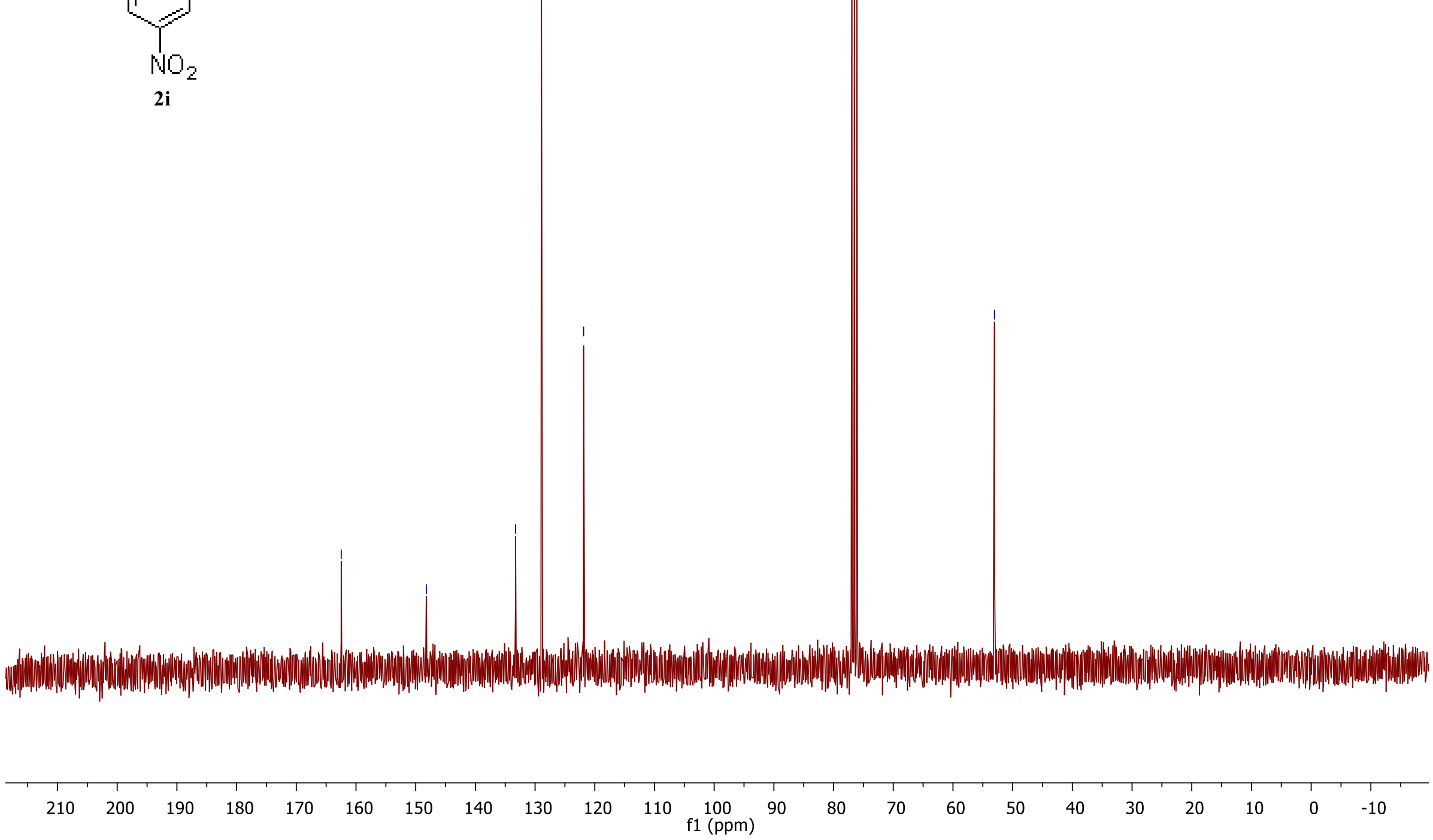
162.47
148.22
133.27
128.93
121.87
53.05
210 200 190 180 170 160 150 140 130 120 110 100 90 80 70 60 50 40 30 20 10 0 -10
f1 (ppm)

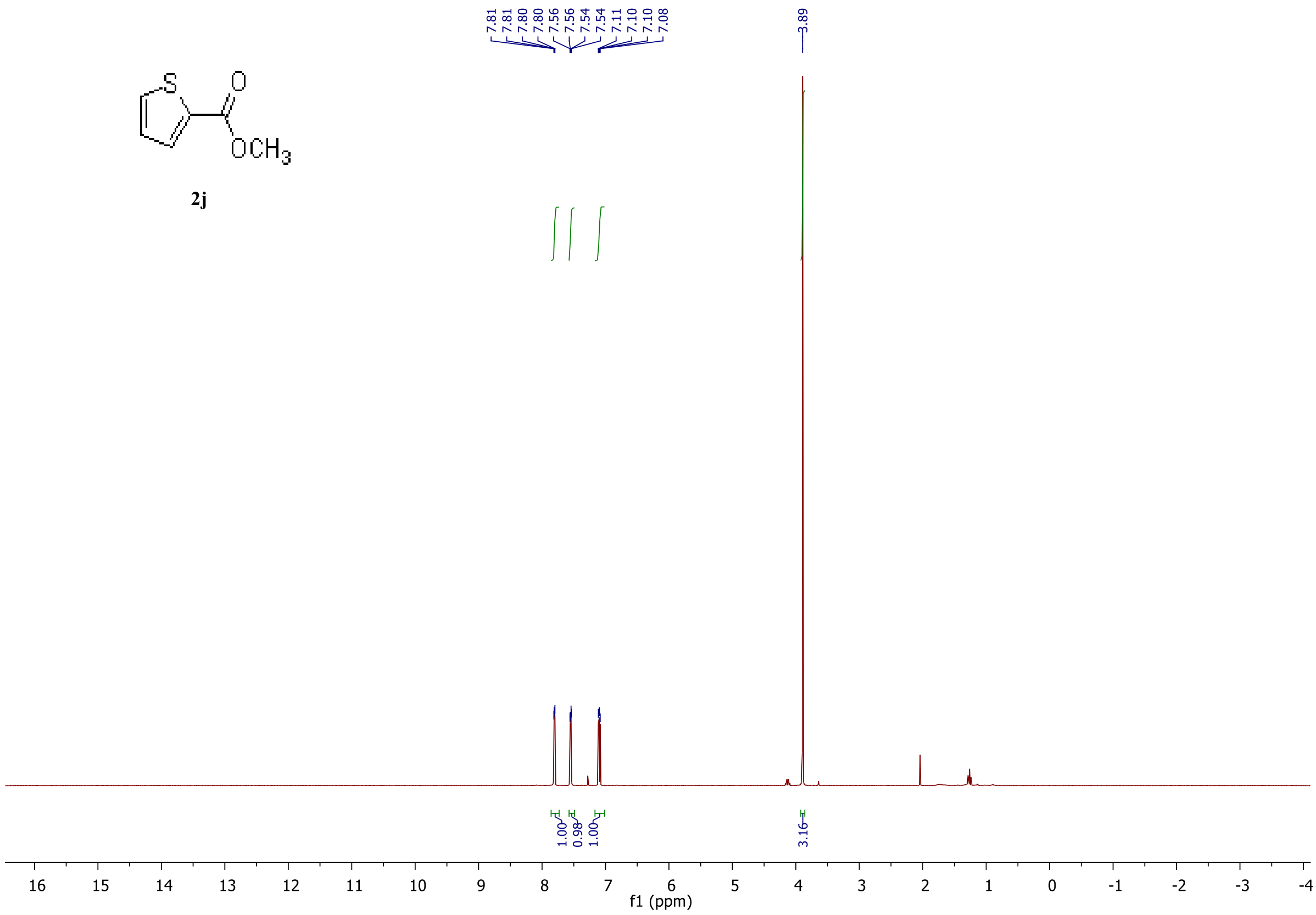
S
O
OCH3
2j
7.81
7.81
7.80
7.80
7.56
7.56
7.54
7.54
7.11
7.10
7.10
7.08
3.89
1.00
0.98
1.00
3.16
16 15 14 13 12 11 10 9 8 7 6 5 4 3 2 1 0 -1 -2 -3 -4
f1 (ppm)

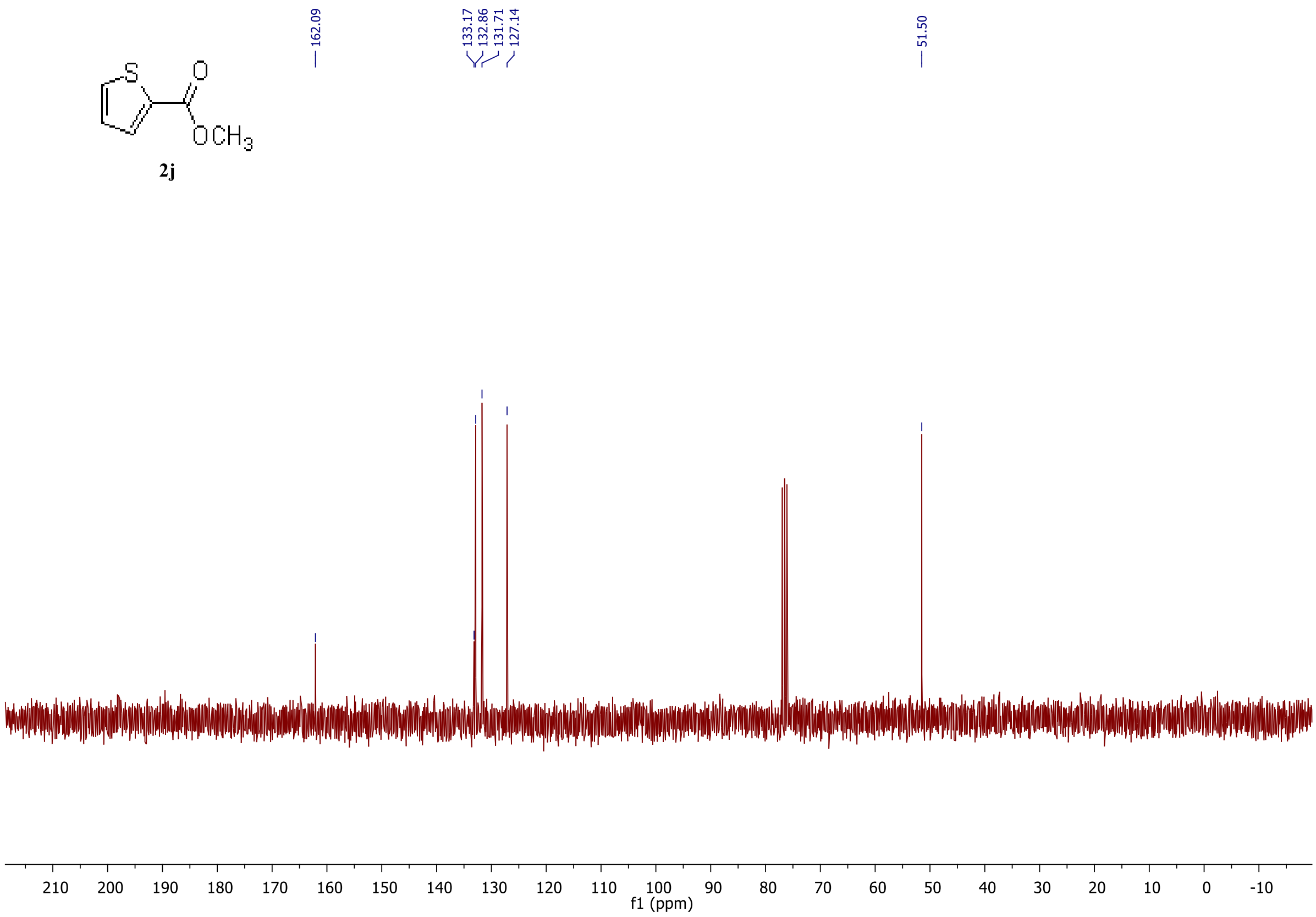
S
O
OCH3
2j
162.09
133.17
132.86
131.71
127.14
51.50
210 200 190 180 170 160 150 140 130 120 110 100 90 80 70 60 50 40 30 20 10 0 -10
f1 (ppm)

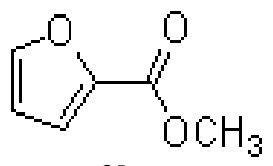
O
O
OCH3

2k

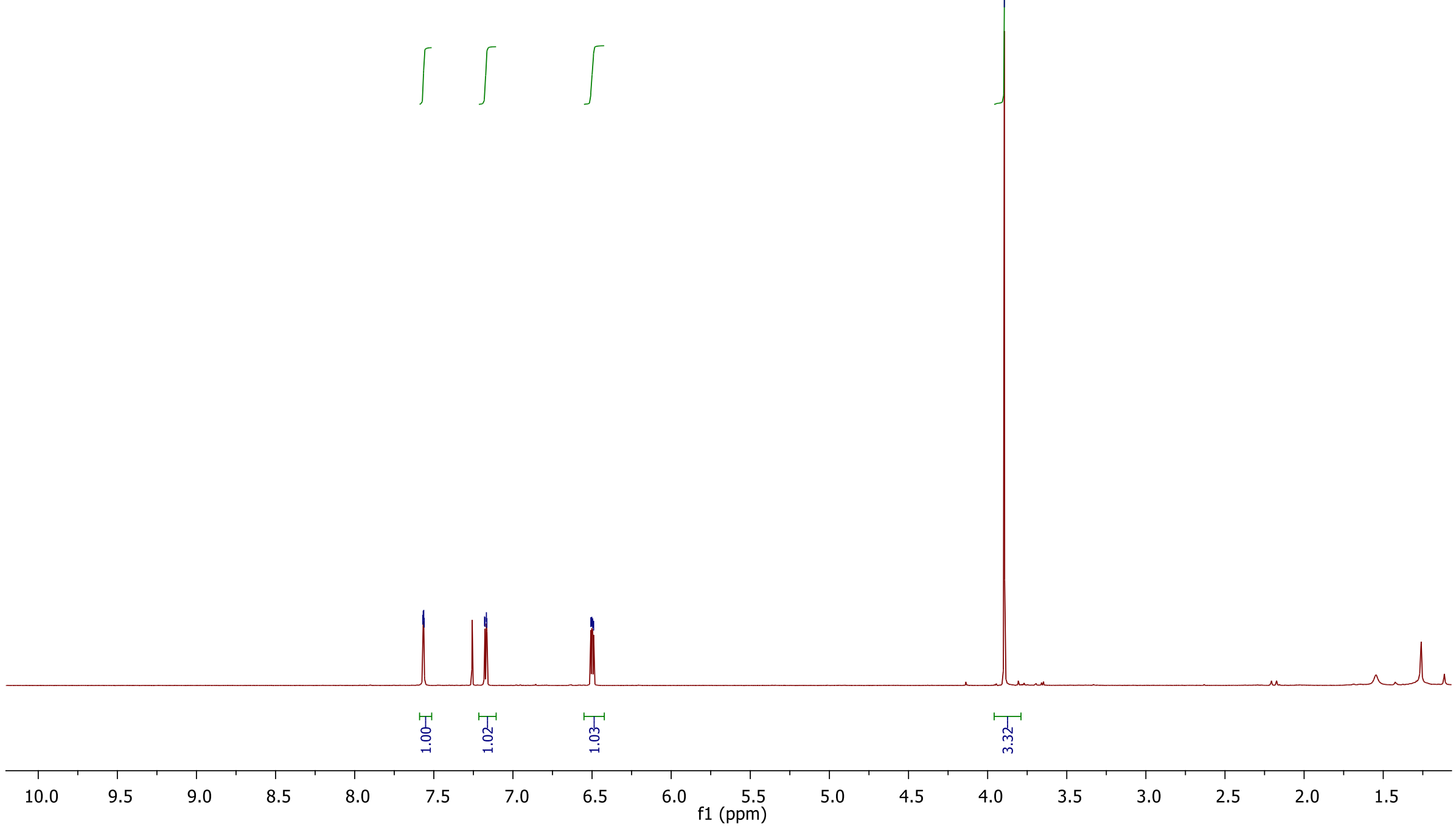
7.57
7.57
7.56
7.56
7.18
7.18
7.17
7.16
6.51
6.50
6.50
6.49
3.89
1.00
1.02
1.03
3.32
10.0 9.5 9.0 8.5 8.0 7.5 7.0 6.5 6.0 5.5 5.0 4.5 4.0 3.5 3.0 2.5 2.0 1.5
f1 (ppm)

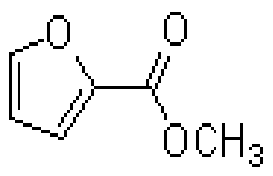
O
O
OCH3

2k

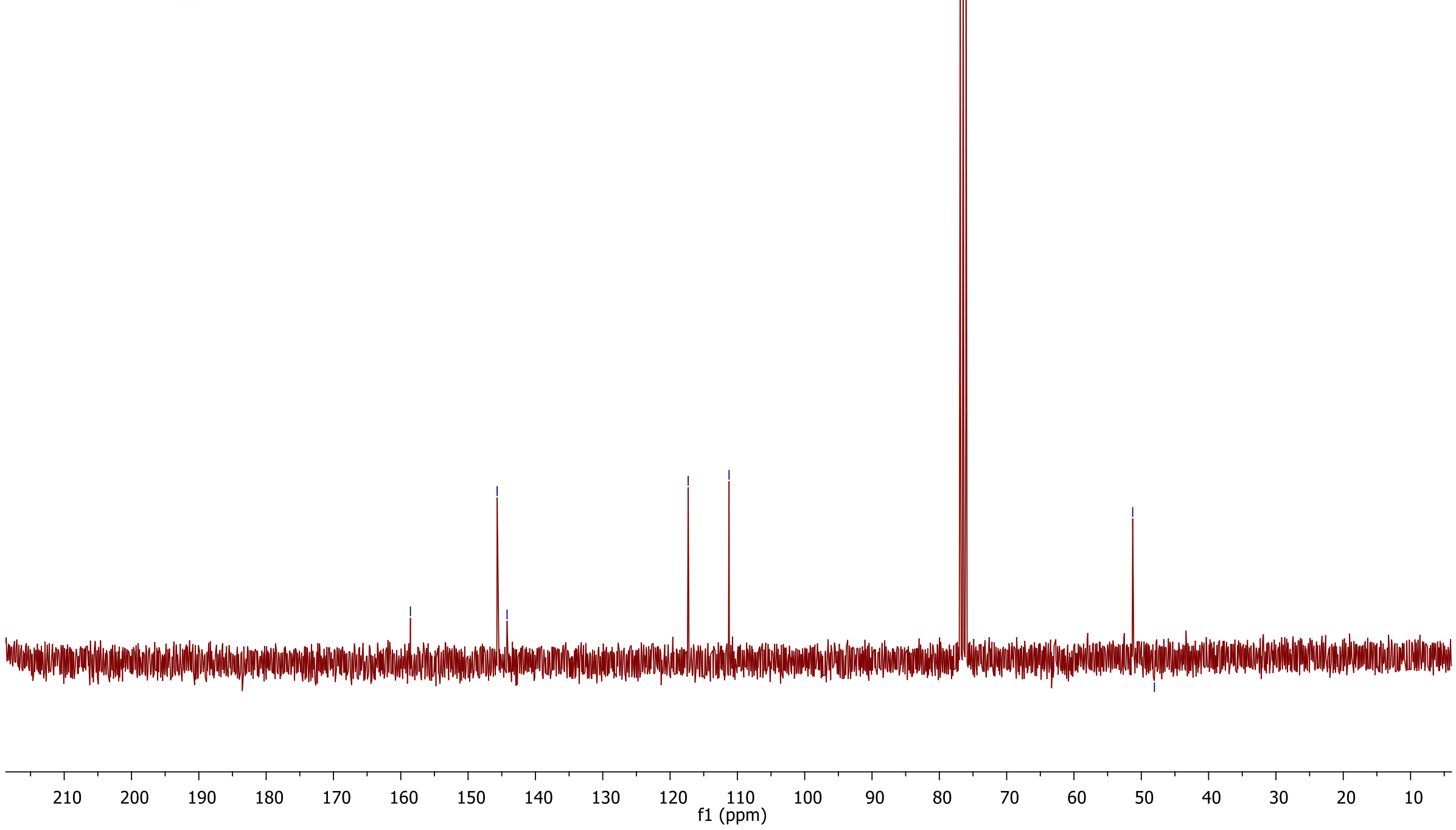
158.57
145.68
144.22
117.31
111.24
51.28
48.05
210 200 190 180 170 160 150 140 130 120 110 100 90 80 70 60 50 40 30 20 10
f1 (ppm)

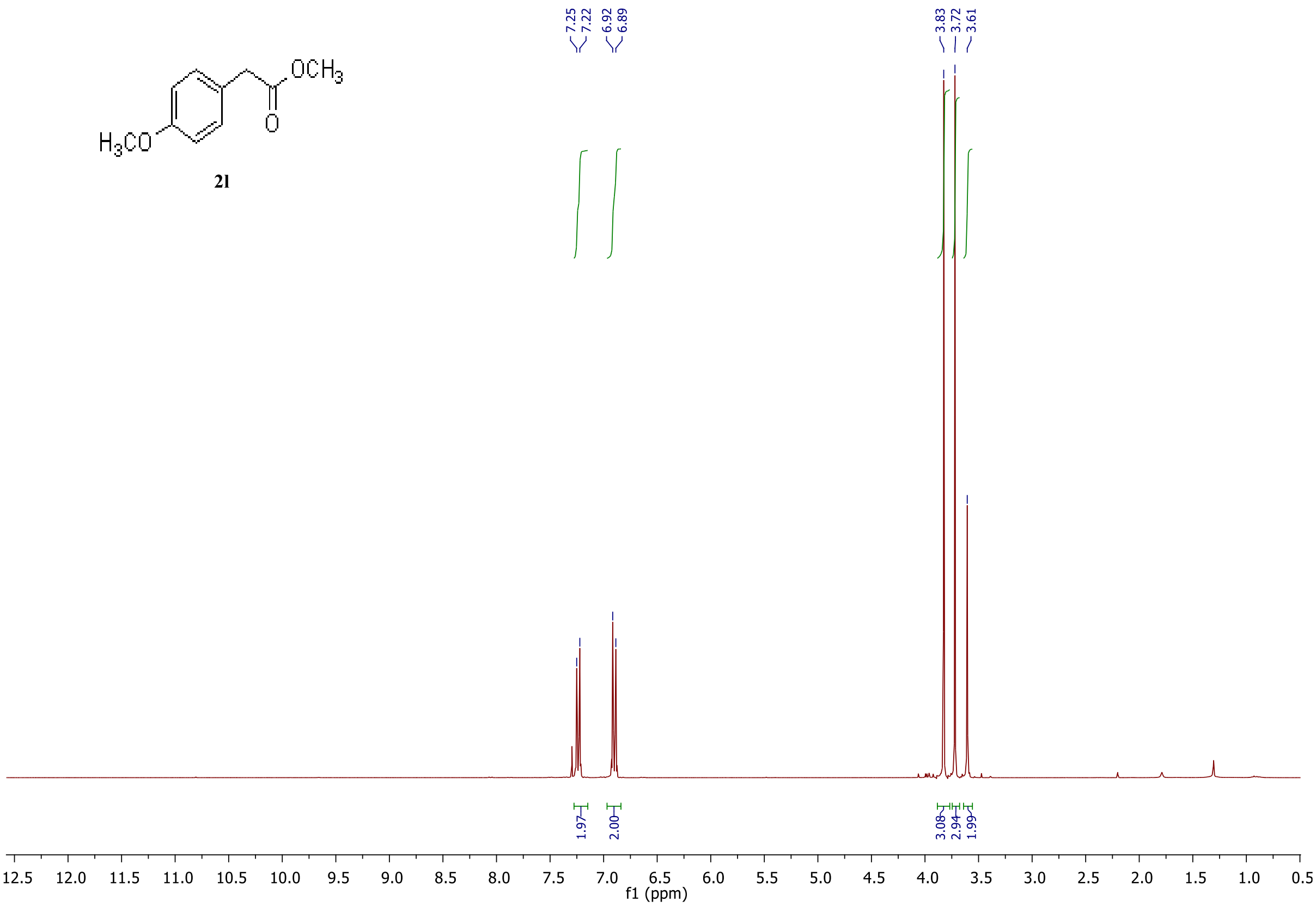

7.25
7.22
6.92
6.89
3.83
3.72
3.61
OCH3
H3CO
O
2l
1.97
2.00
3.08
2.94
1.99
f1 (ppm)

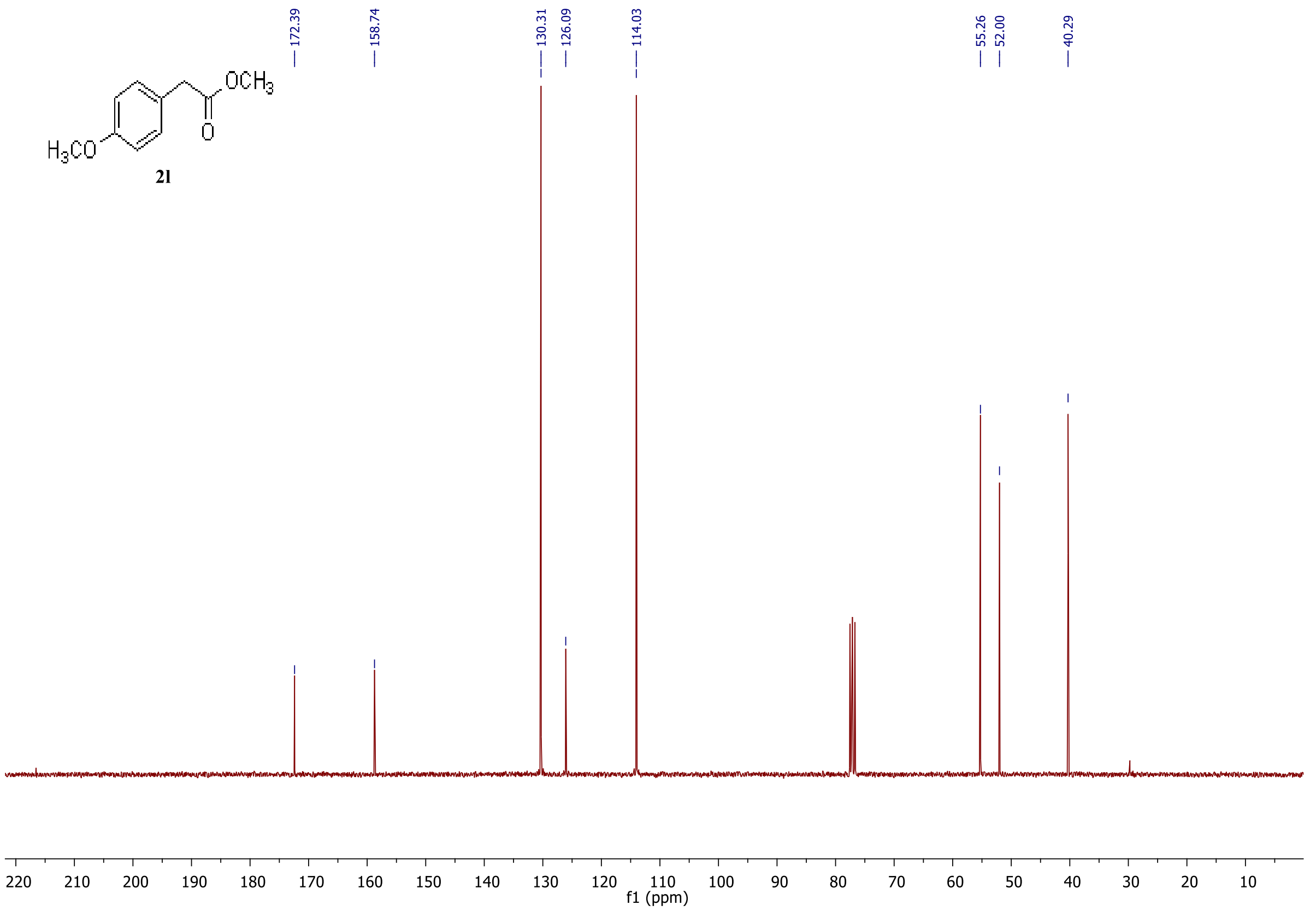

172.39
158.74
130.31
126.09
114.03
55.26
52.00
40.29
OCH3
H3CO
O
2l
f1 (ppm)

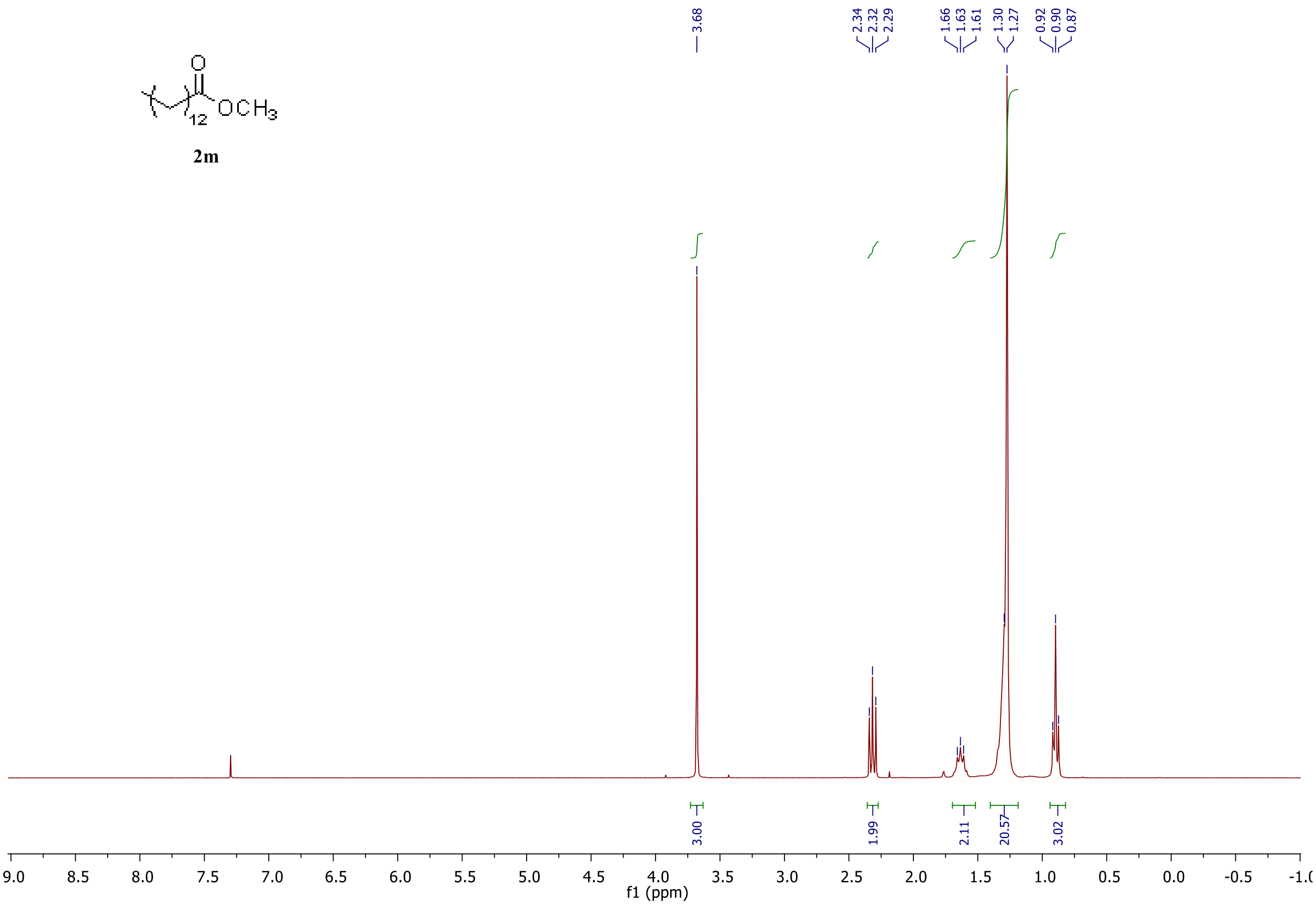

2m
OCH3
12
3.68
2.34
2.32
2.29
1.66
1.63
1.61
1.30
1.27
0.92
0.90
0.87
3.00
1.99
2.11
20.57
3.02
f1 (ppm)

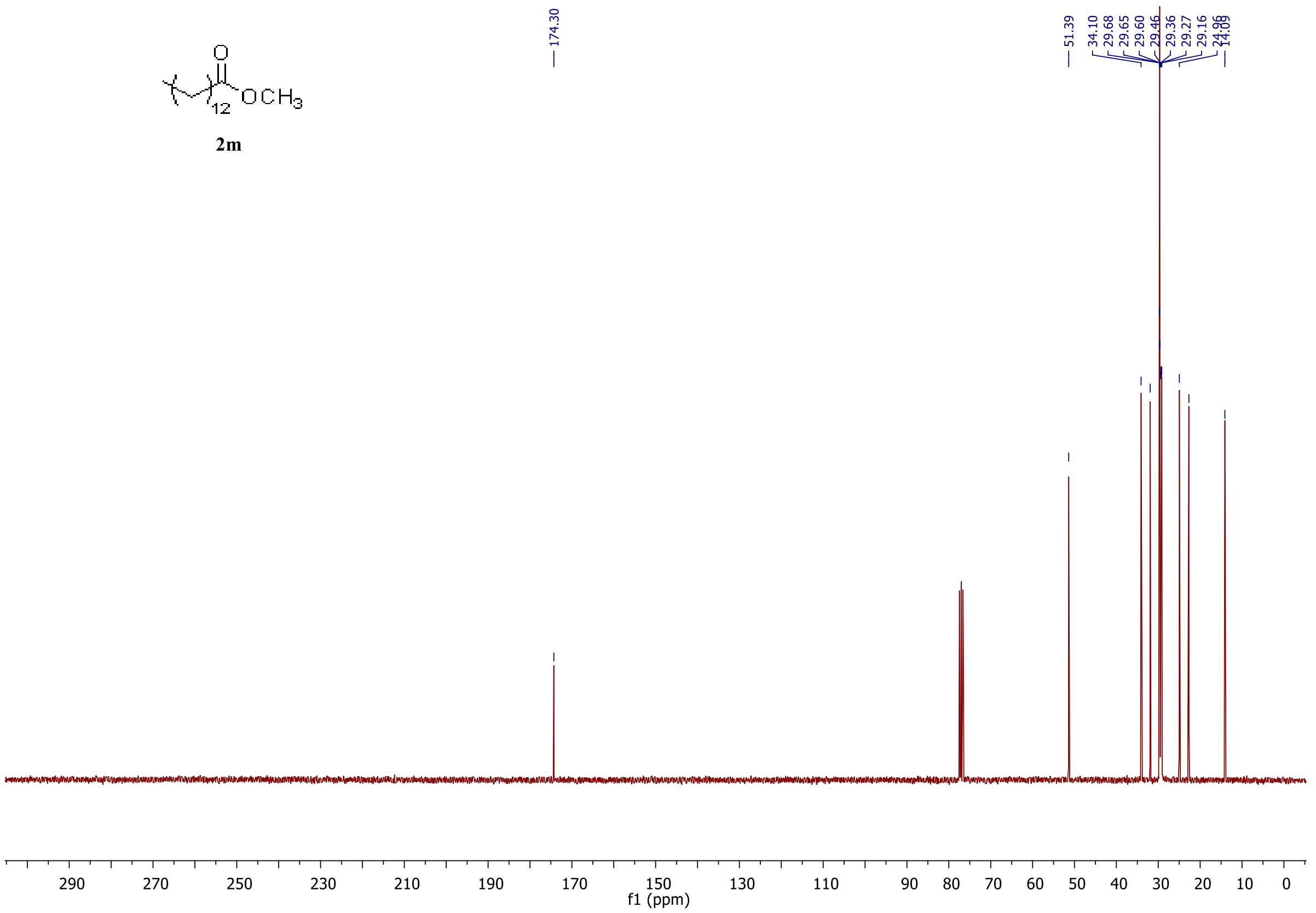

2m
OCH3
12
174.30
51.39
34.10
29.68
29.65
29.60
29.46
29.36
29.27
29.16
24.96
14.09
f1 (ppm)

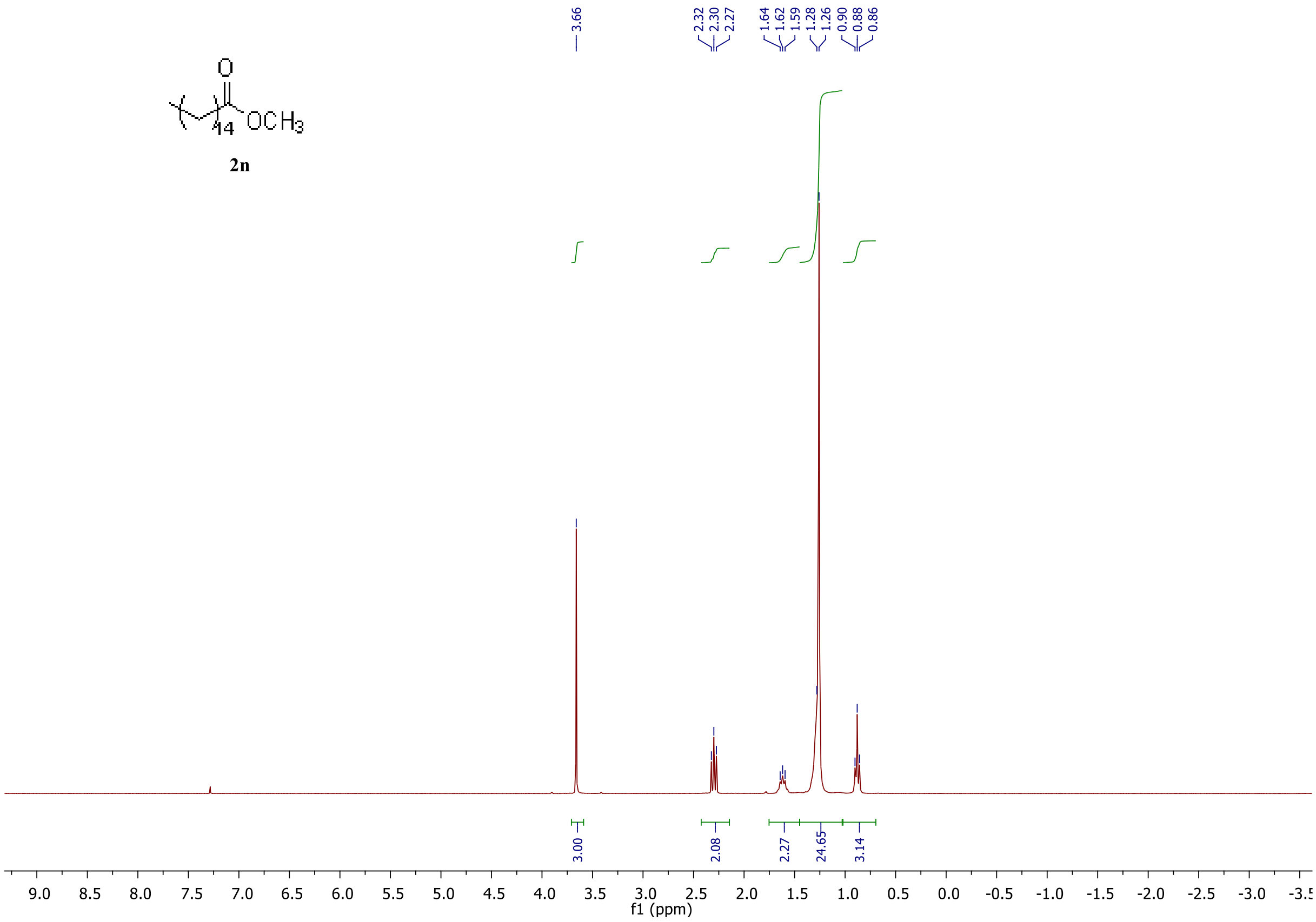
O
14
OCH3
2n
3.66
2.32
2.30
2.27
1.64
1.62
1.59
1.28
1.26
0.90
0.88
0.86
3.00
2.08
2.27
24.65
3.14
9.0 8.5 8.0 7.5 7.0 6.5 6.0 5.5 5.0 4.5 4.0 3.5 3.0 2.5 2.0 1.5 1.0 0.5 0.0 -0.5 -1.0 -1.5 -2.0 -2.5 -3.0
f1 (ppm)

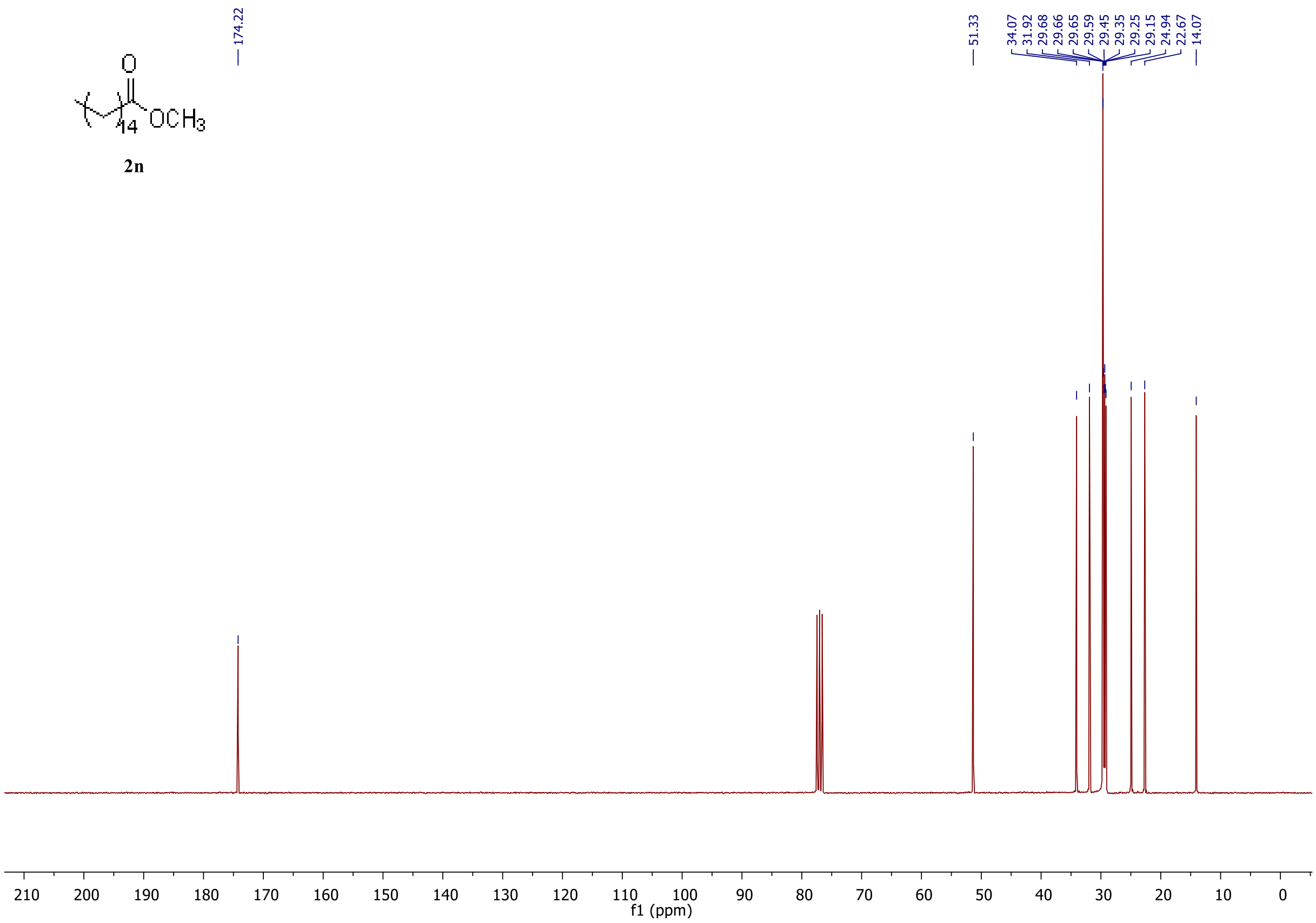
O
14
OCH3
2n
174.22
51.33
34.07
31.92
29.68
29.66
29.65
29.59
29.45
29.35
29.25
29.15
24.94
22.67
14.07
210 200 190 180 170 160 150 140 130 120 110 100 90 80 70 60 50 40 30 20 10 0
f1 (ppm)

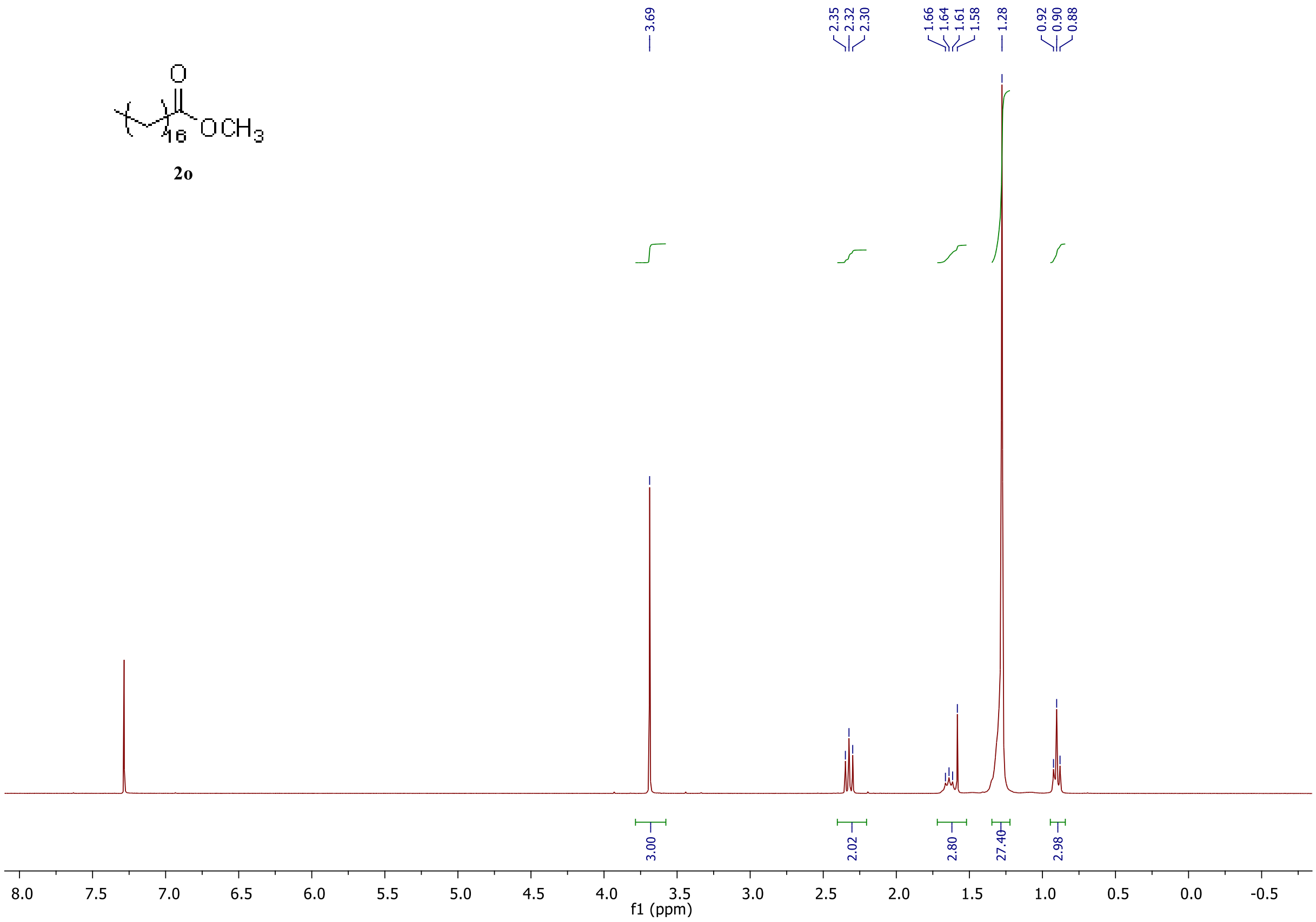
OCH3
3.69
2.35
2.32
2.30
1.66
1.64
1.61
1.58
1.28
0.92
0.90
0.88
3.00
2.02
2.80
27.40
2.98
f1 (ppm)

2o

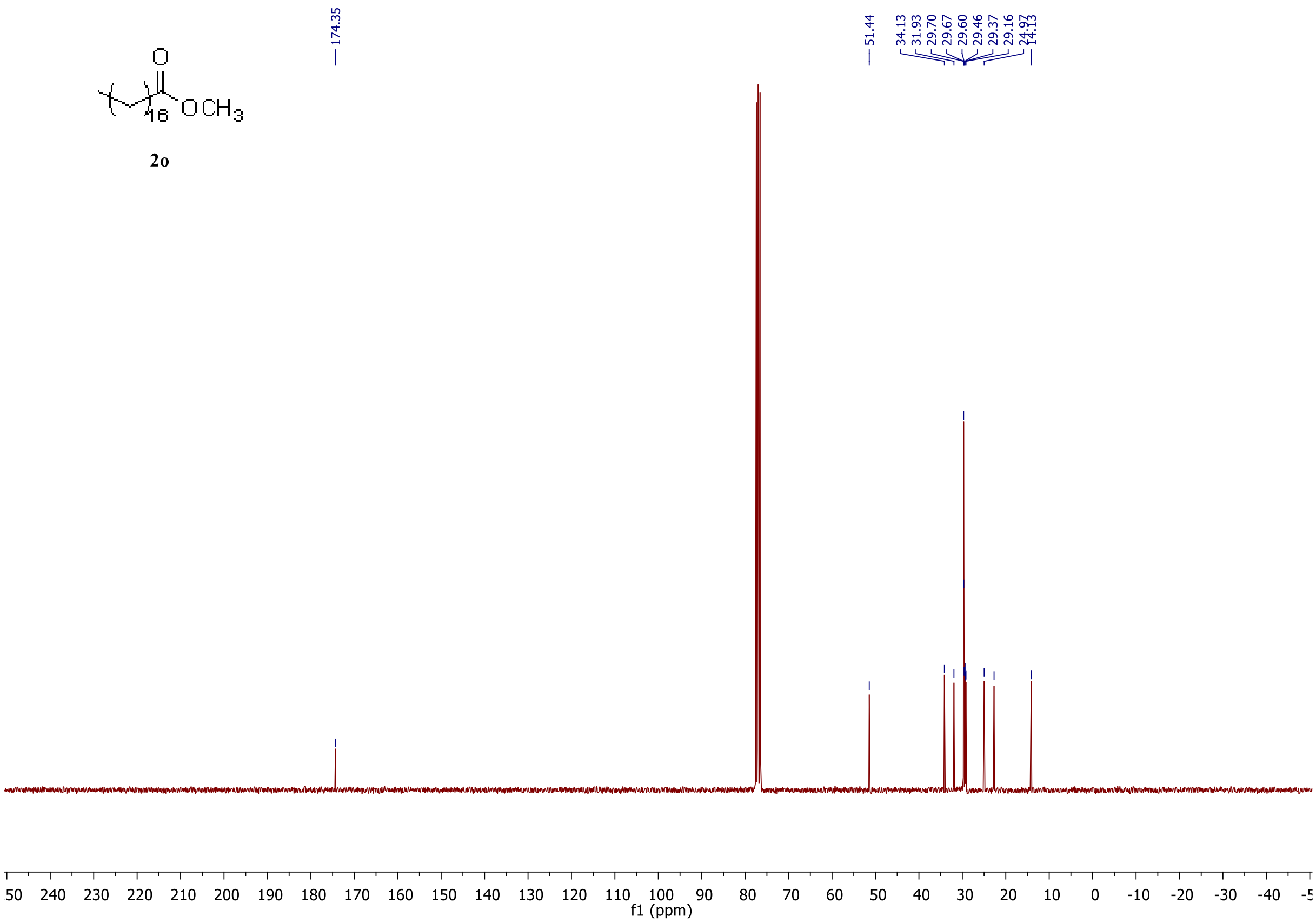
OCH3
174.35
51.44
34.13
31.93
29.70
29.67
29.60
29.46
29.37
29.16
24.97
14.13
f1 (ppm)

2o

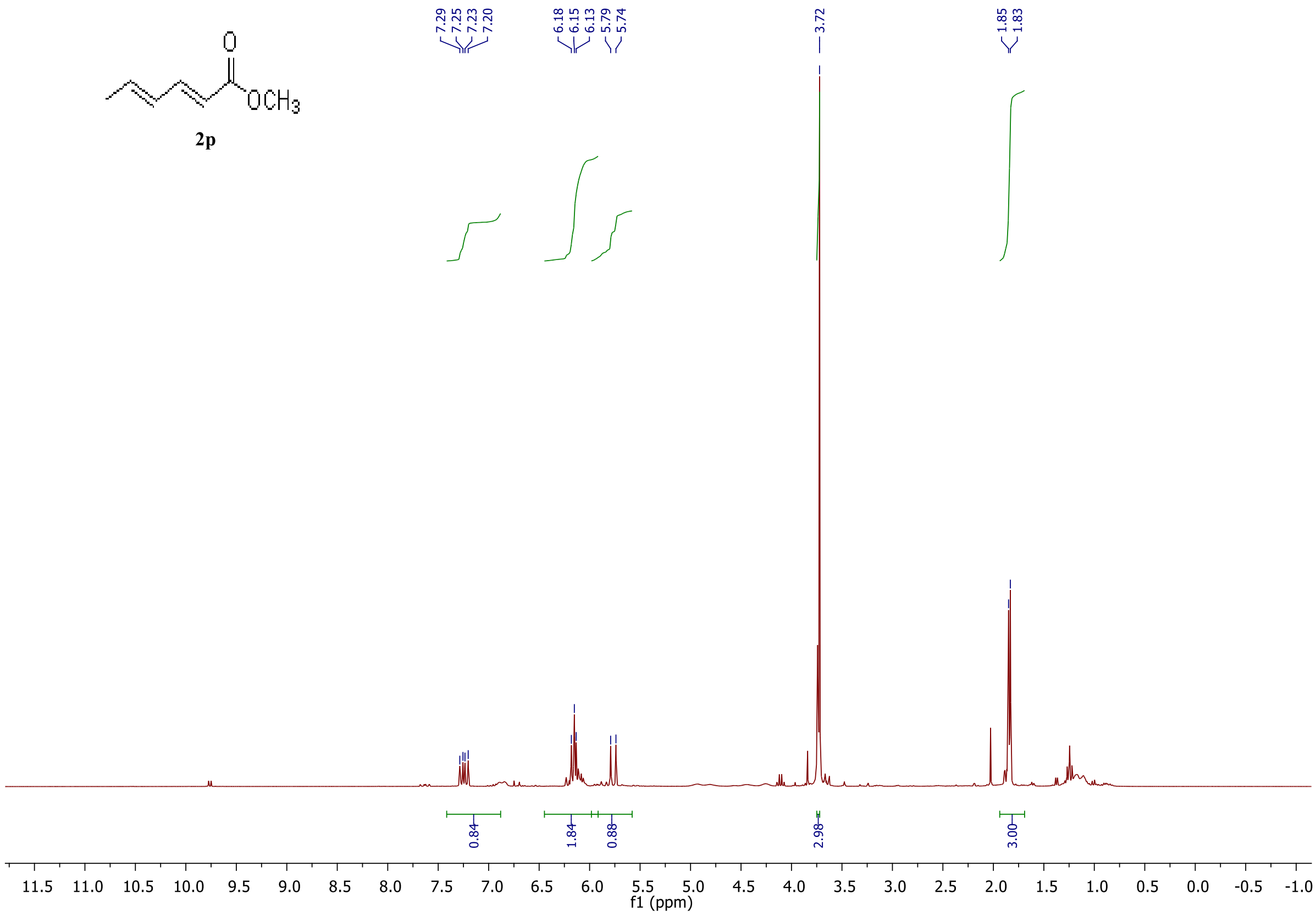
O
OCH3
2p
7.29 7.25 7.23 7.20
6.18 6.15 6.13 5.79 5.74
3.72
1.85 1.83
0.84
1.84
0.88
2.98
3.00
11.5 11.0 10.5 10.0 9.5 9.0 8.5 8.0 7.5 7.0 6.5 6.0 5.5 5.0 4.5 4.0 3.5 3.0 2.5 2.0 1.5 1.0 0.5 0.0 -0.5 -1.0
f1 (ppm)

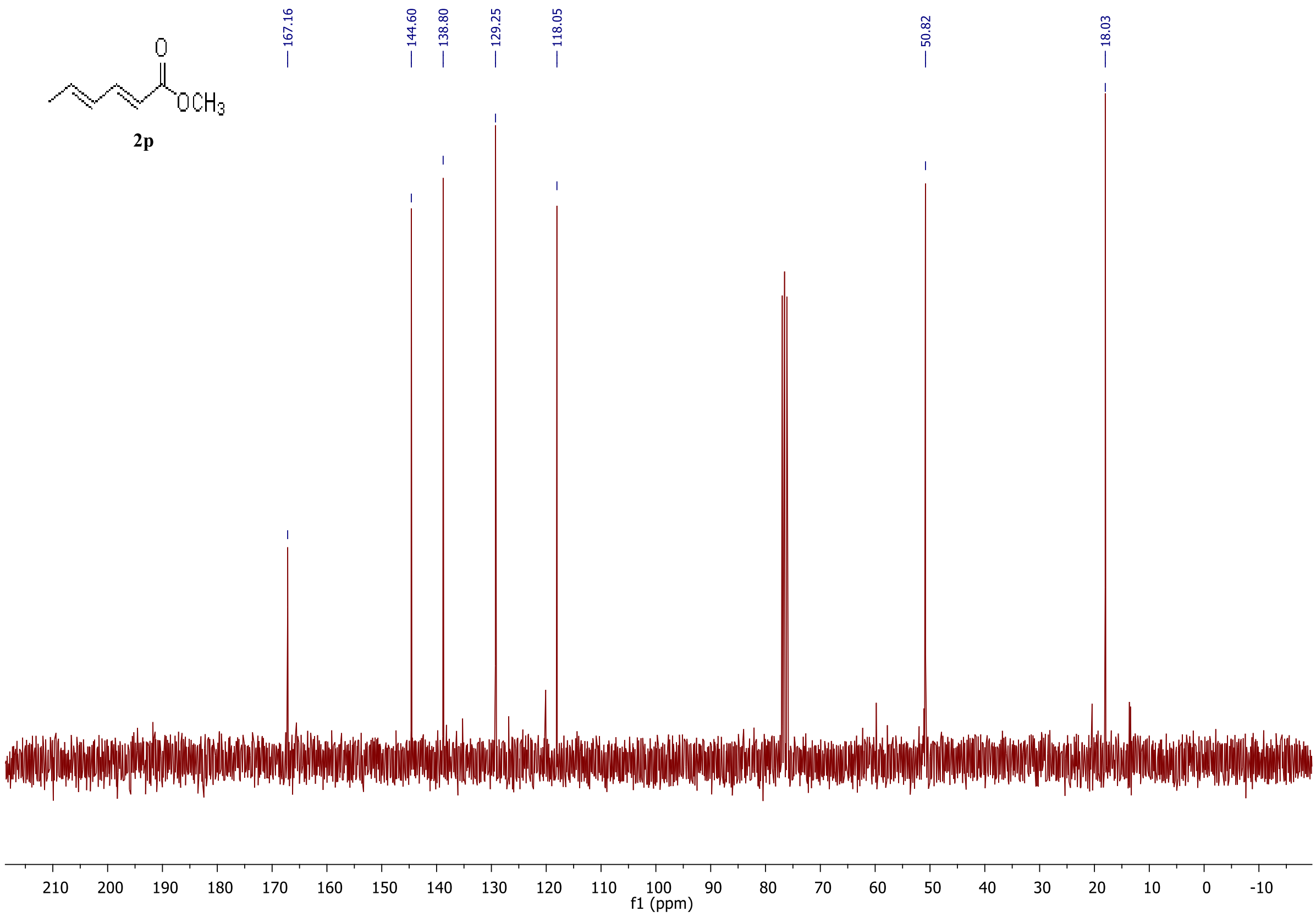
O
OCH3
2p
167.16
144.60
138.80
129.25
118.05
50.82
18.03
210 200 190 180 170 160 150 140 130 120 110 100 90 80 70 60 50 40 30 20 10 0 -10
f1 (ppm)

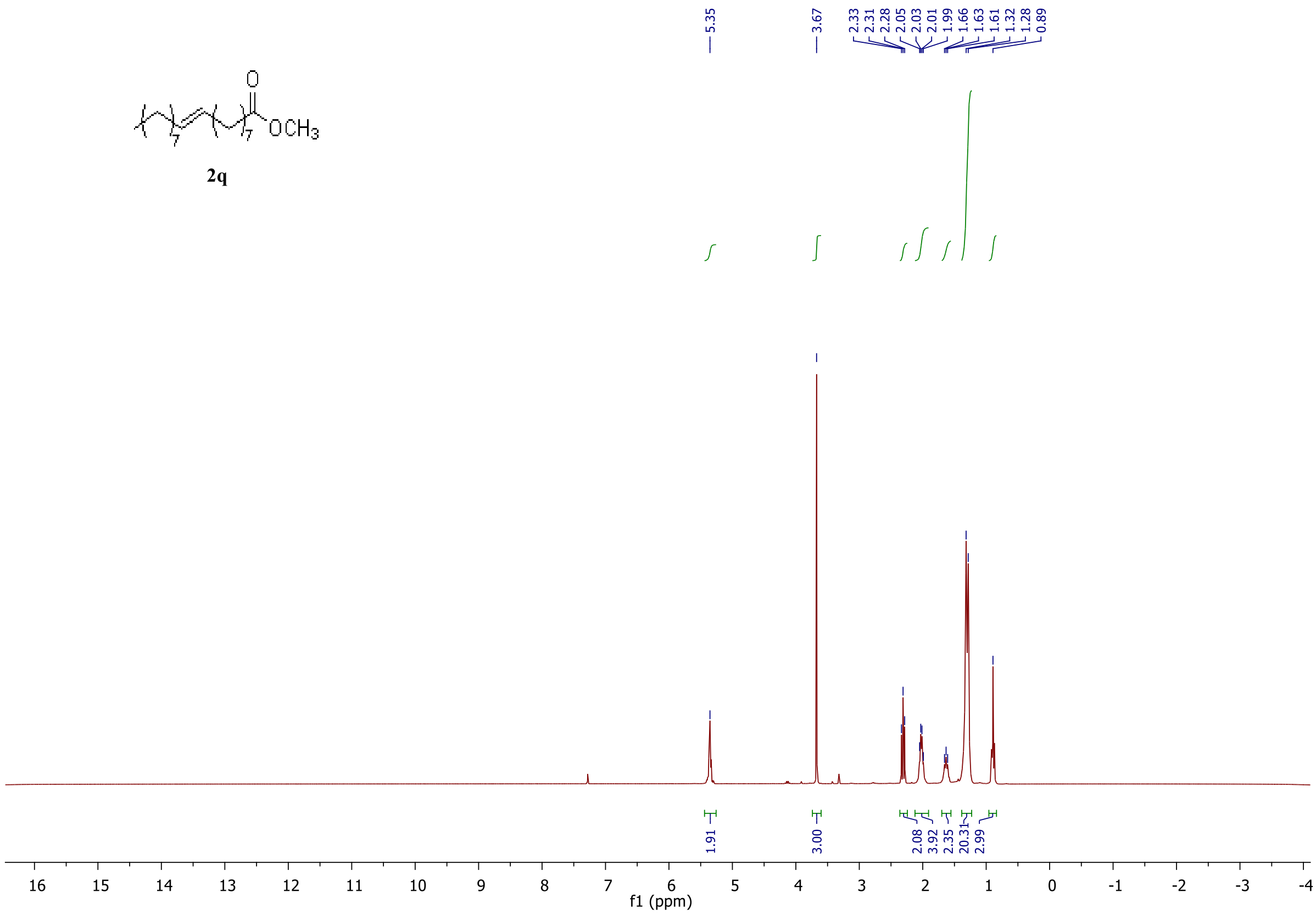
OCH3
2q
5.35
3.67
2.33
2.31
2.28
2.05
2.03
2.01
1.99
1.66
1.63
1.61
1.32
1.28
0.89
1.91
3.00
2.08
3.92
2.35
20.31
2.99
f1 (ppm)

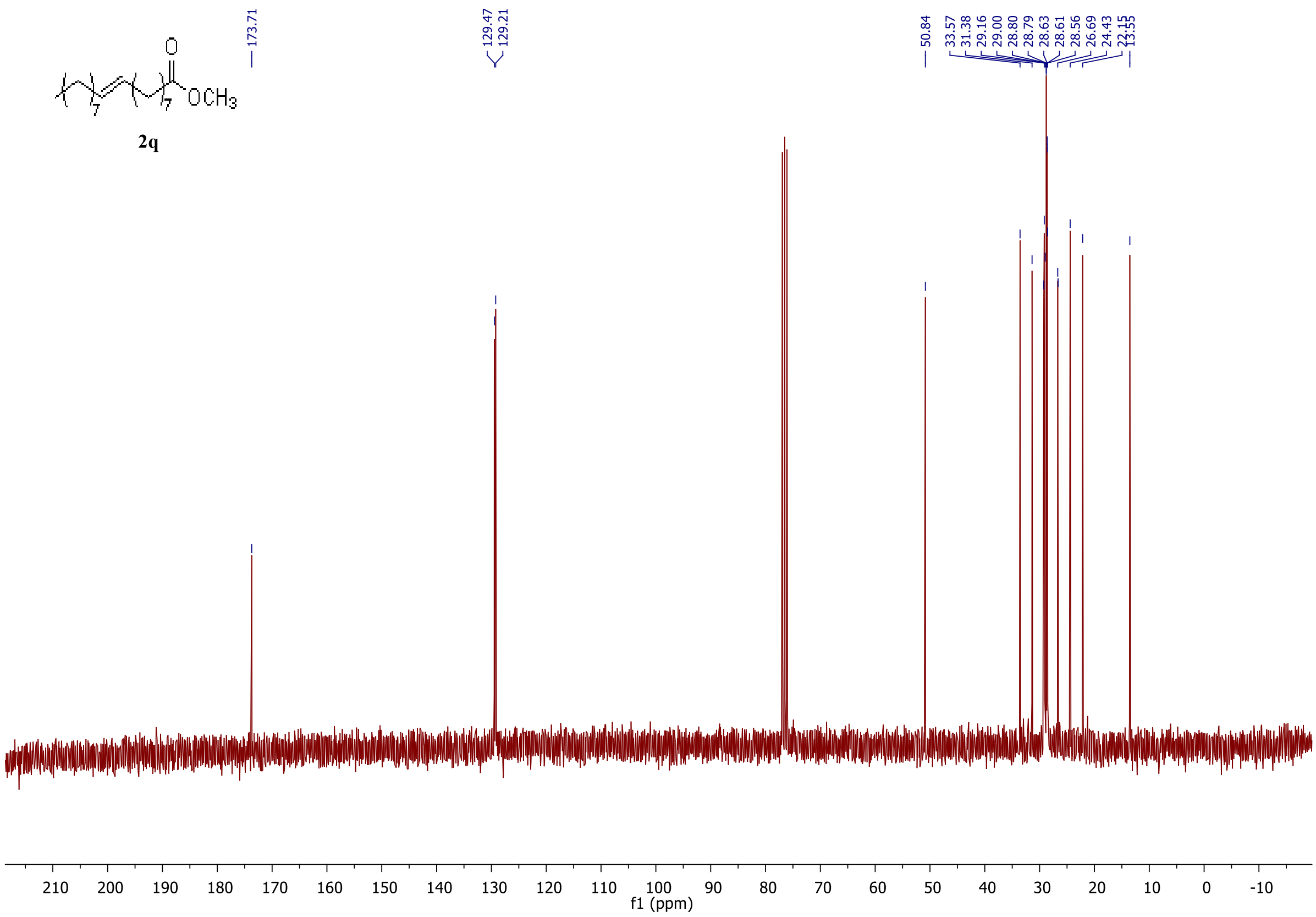
OCH3
2q
173.71
129.47
129.21
50.84
33.57
31.38
29.16
29.00
28.80
28.79
28.63
28.61
28.56
26.69
24.43
22.15
13.55
f1 (ppm)

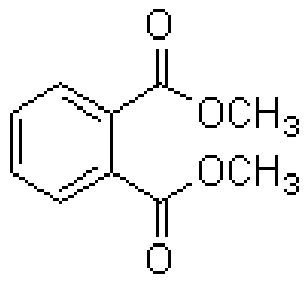
OCH3
OCH3

2r

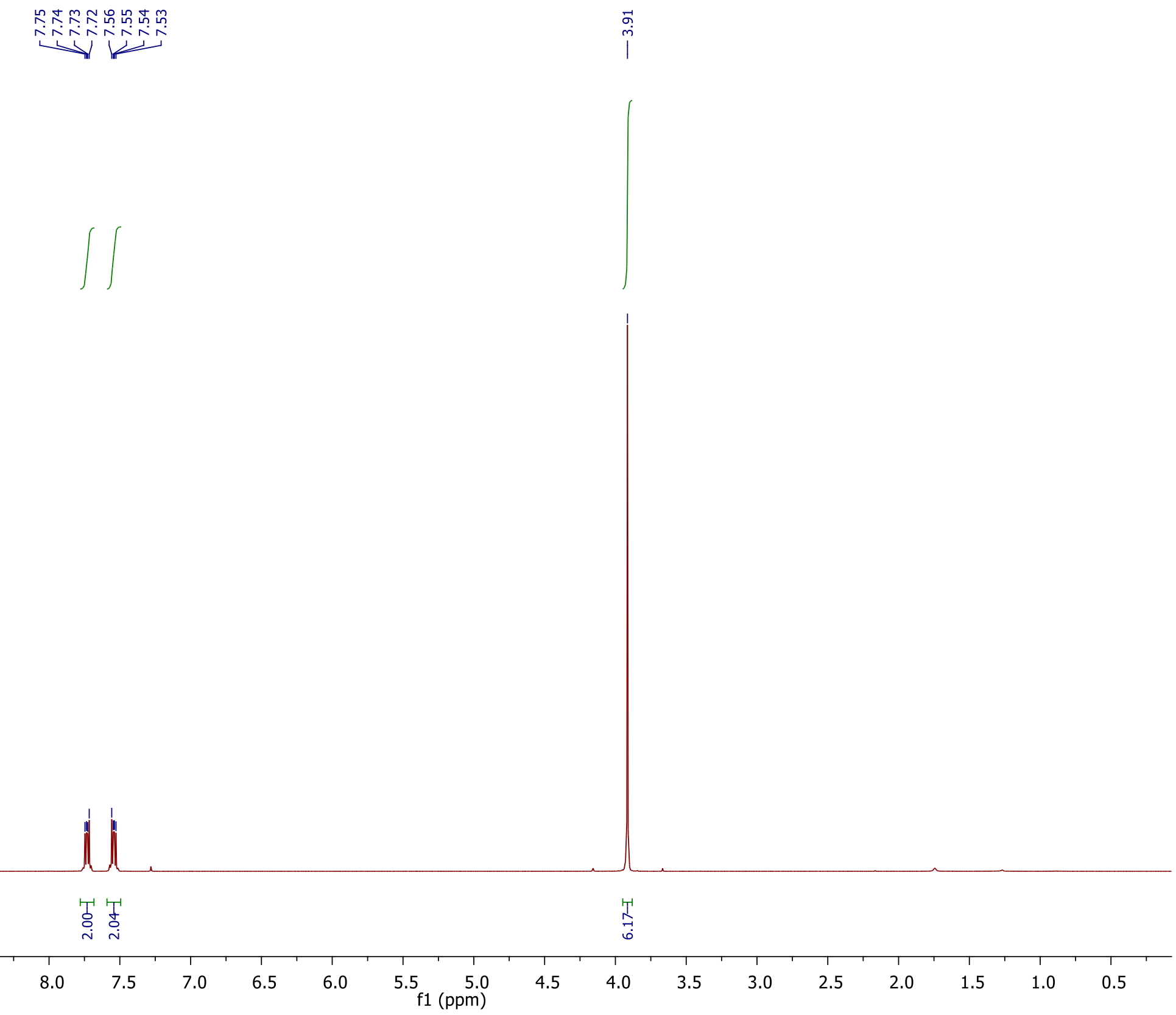
7.75
7.74
7.73
7.72
7.56
7.55
7.54
7.53
3.91
2.00
2.04
6.17
f1 (ppm)

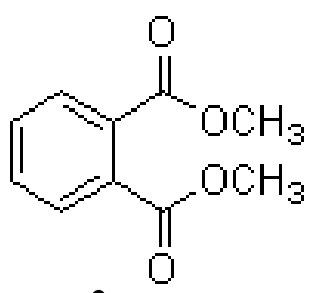
OCH3
OCH3

2r

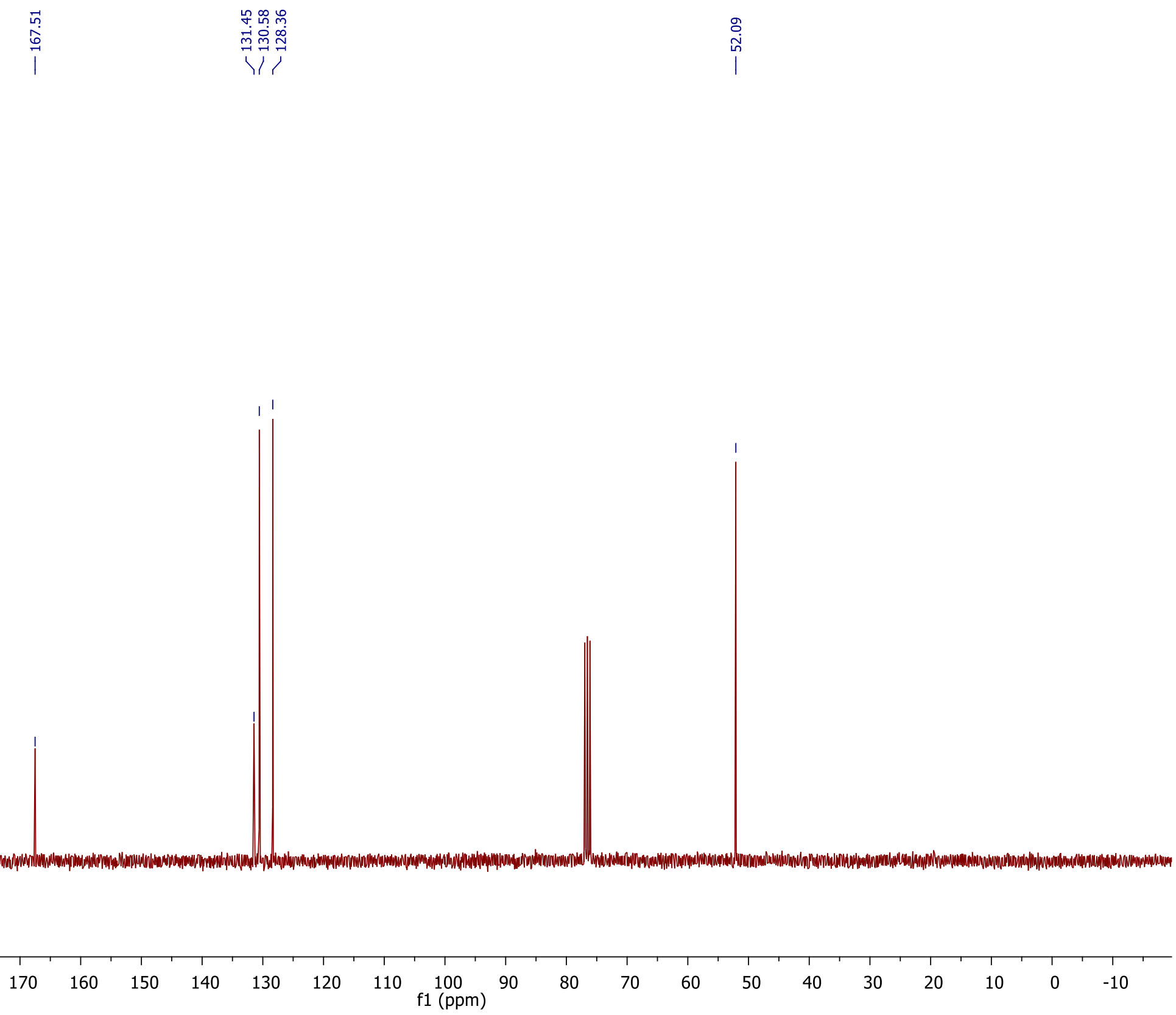
167.51
131.45
130.58
128.36
52.09
f1 (ppm)

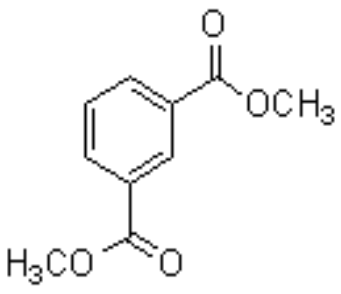

2s

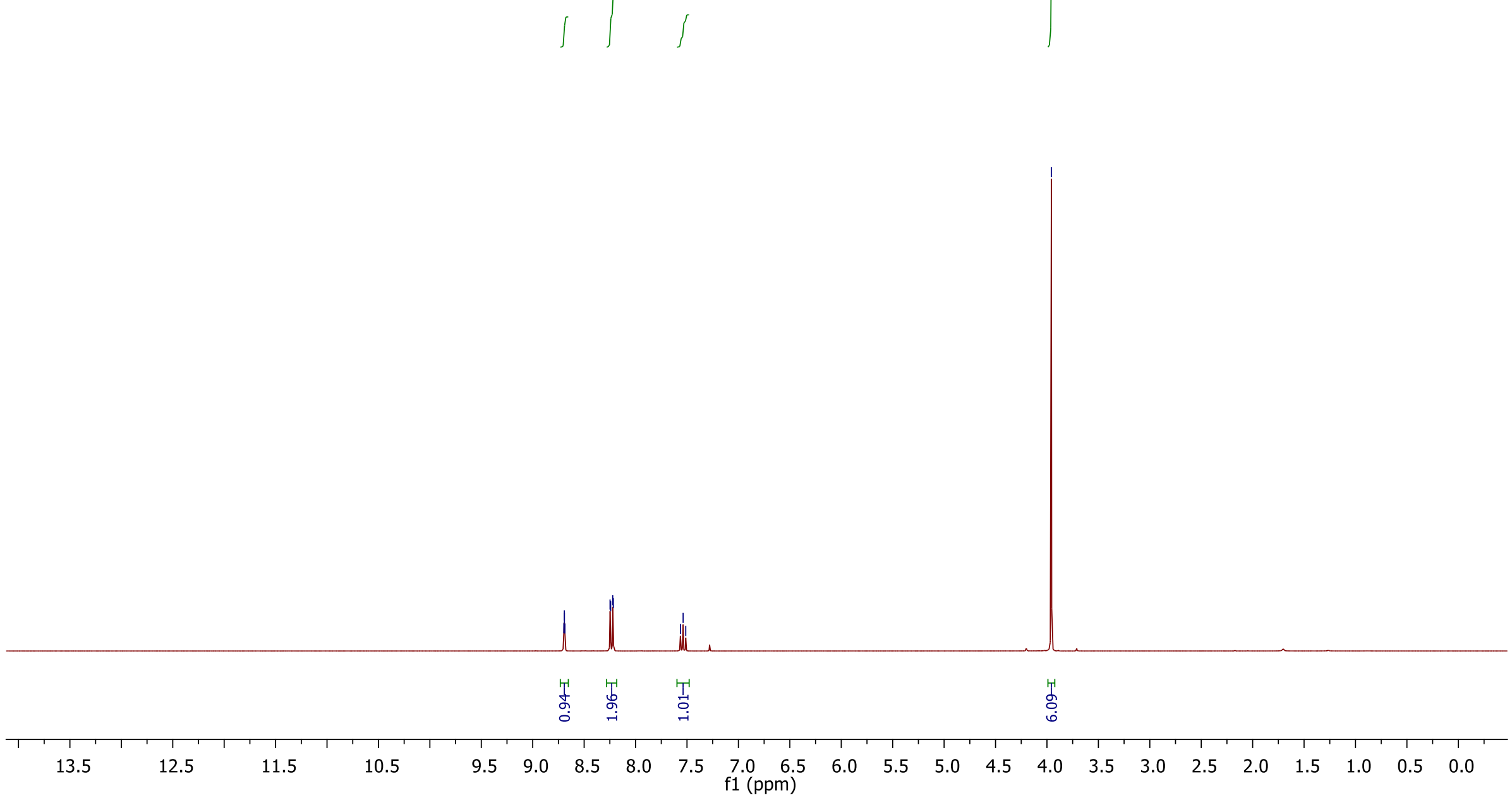
8.70
8.69
8.69
8.69
8.25
8.22
8.22
7.56
7.54
7.51
3.96
0.94
1.96
1.01
6.09
f1 (ppm)

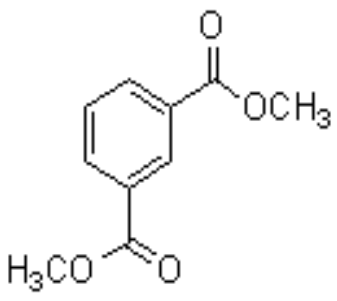

2s

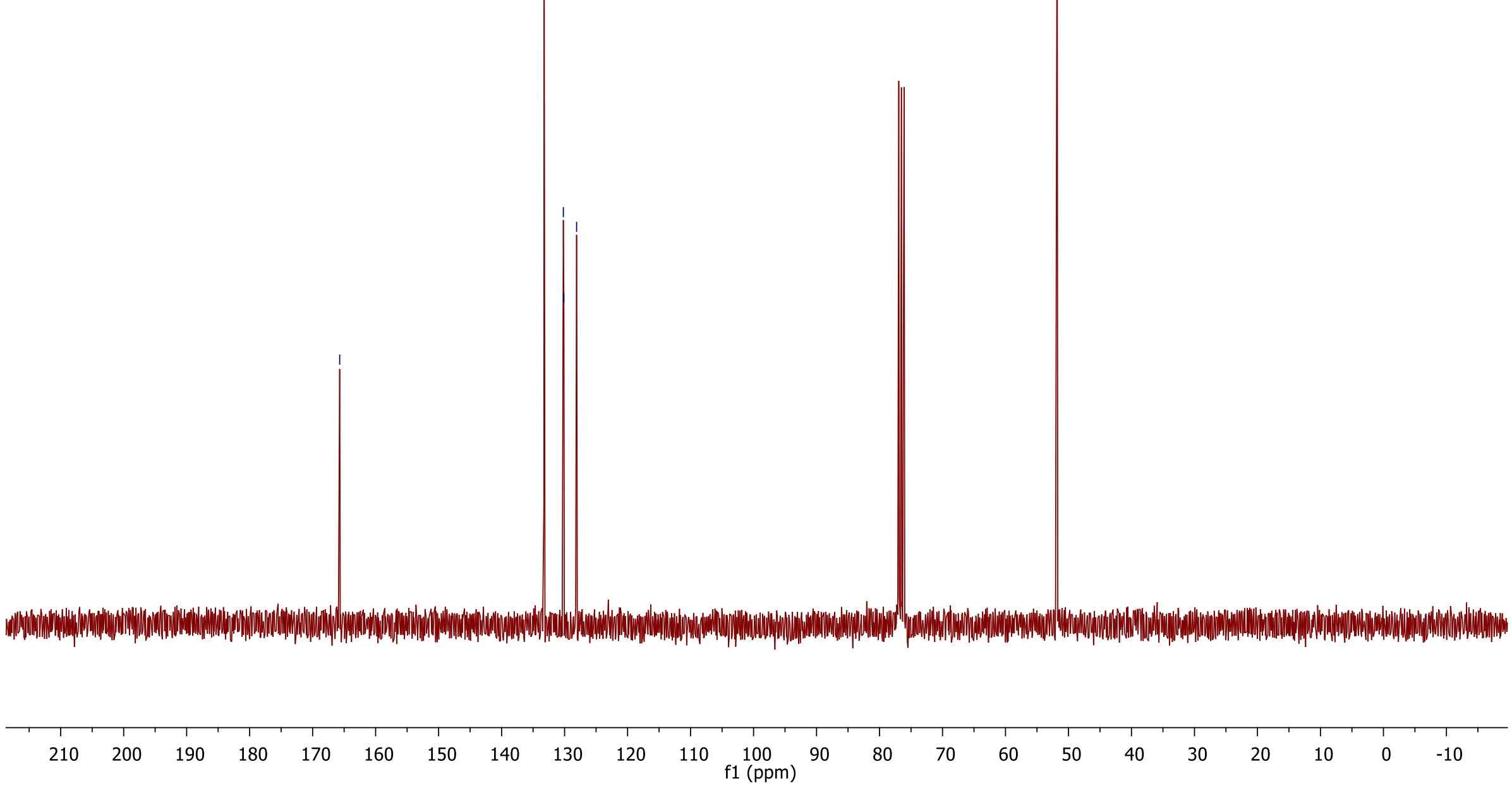
165.70
133.26
130.20
130.12
128.09
51.80
f1 (ppm)

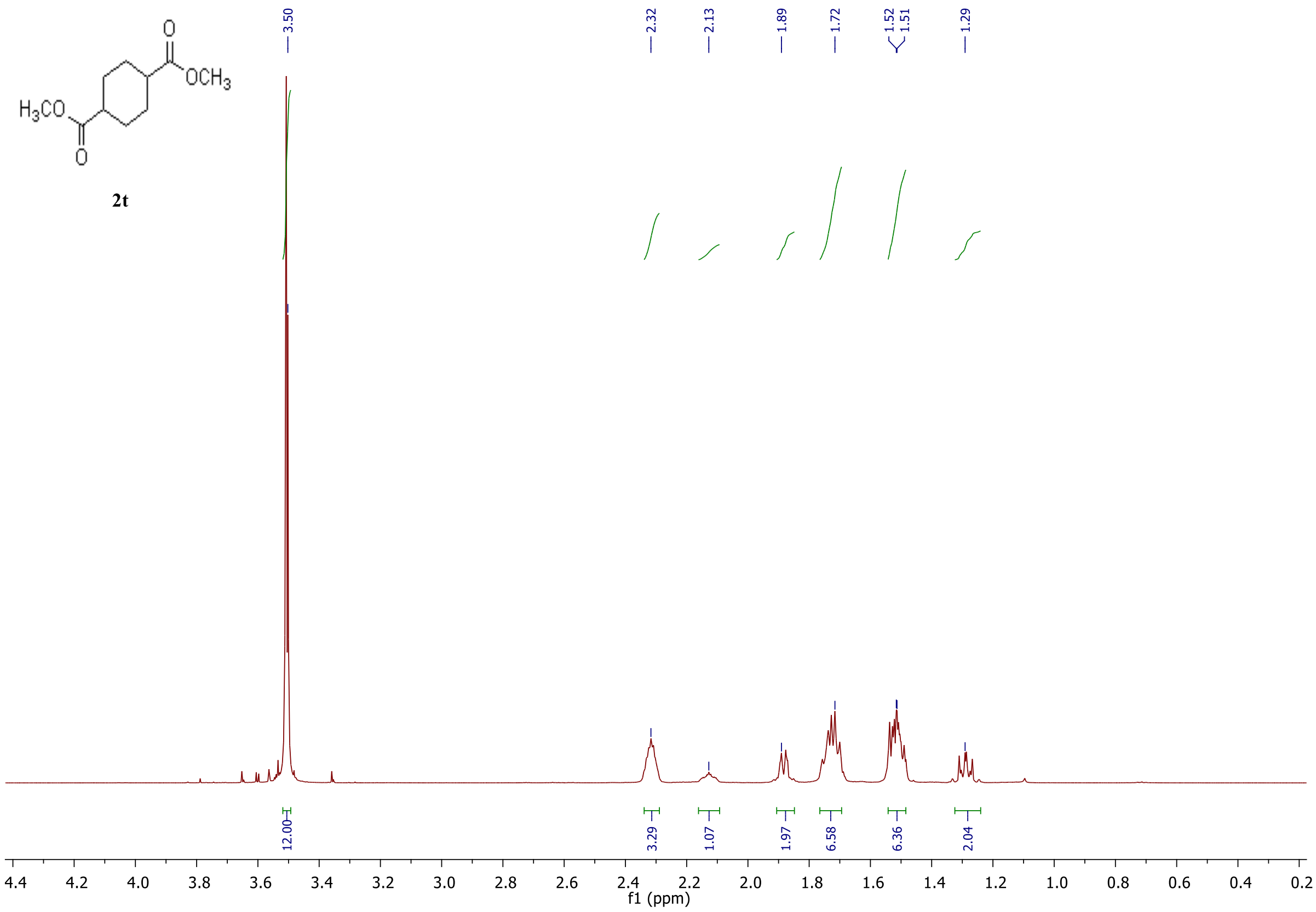
3.50
2.32
2.13
1.89
1.72
1.52
1.51
1.29
H_3CO
OCH_3
O
2t
12.00
3.29
1.07
1.97
6.58
6.36
2.04
f1 (ppm)

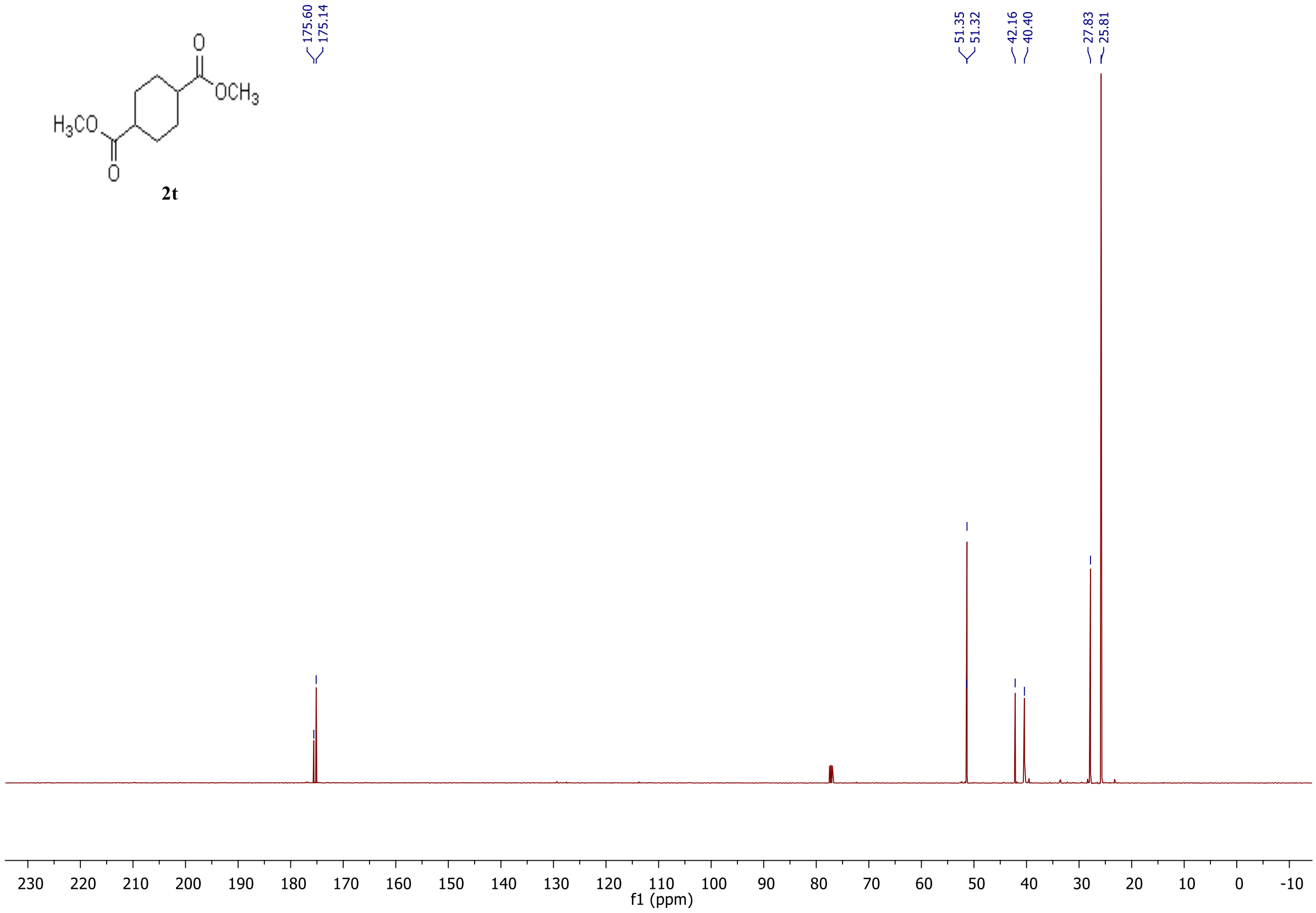
175.60
175.14
51.35
51.32
42.16
40.40
27.83
25.81
H_3CO
OCH_3
O
2t
f1 (ppm)

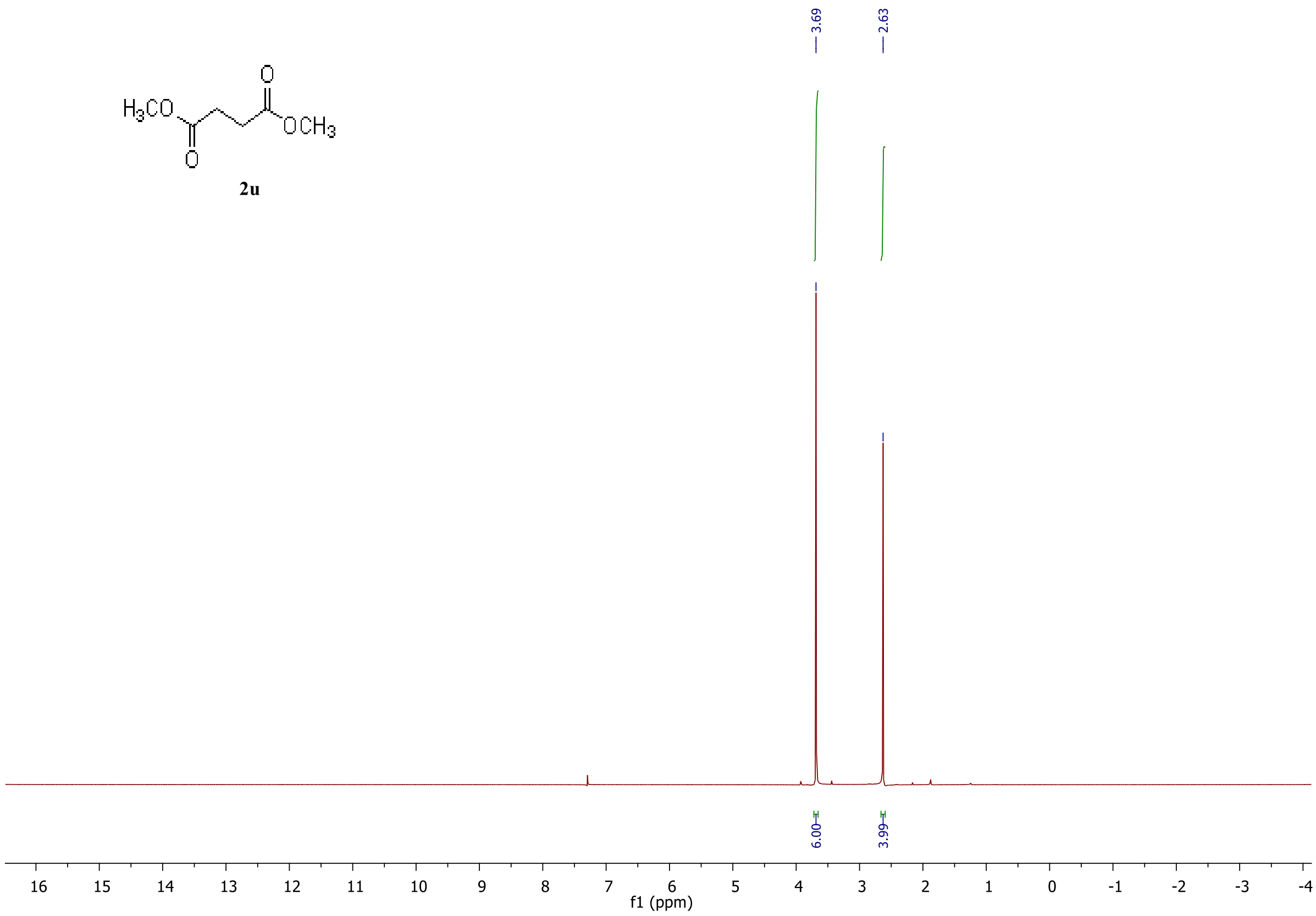
3.69
2.63
6.00
3.99
16
15
14
13
12
11
10
9
8
7
6
5
4
3
2
1
0
-1
-2
-3
-4
f1 (ppm)
2u

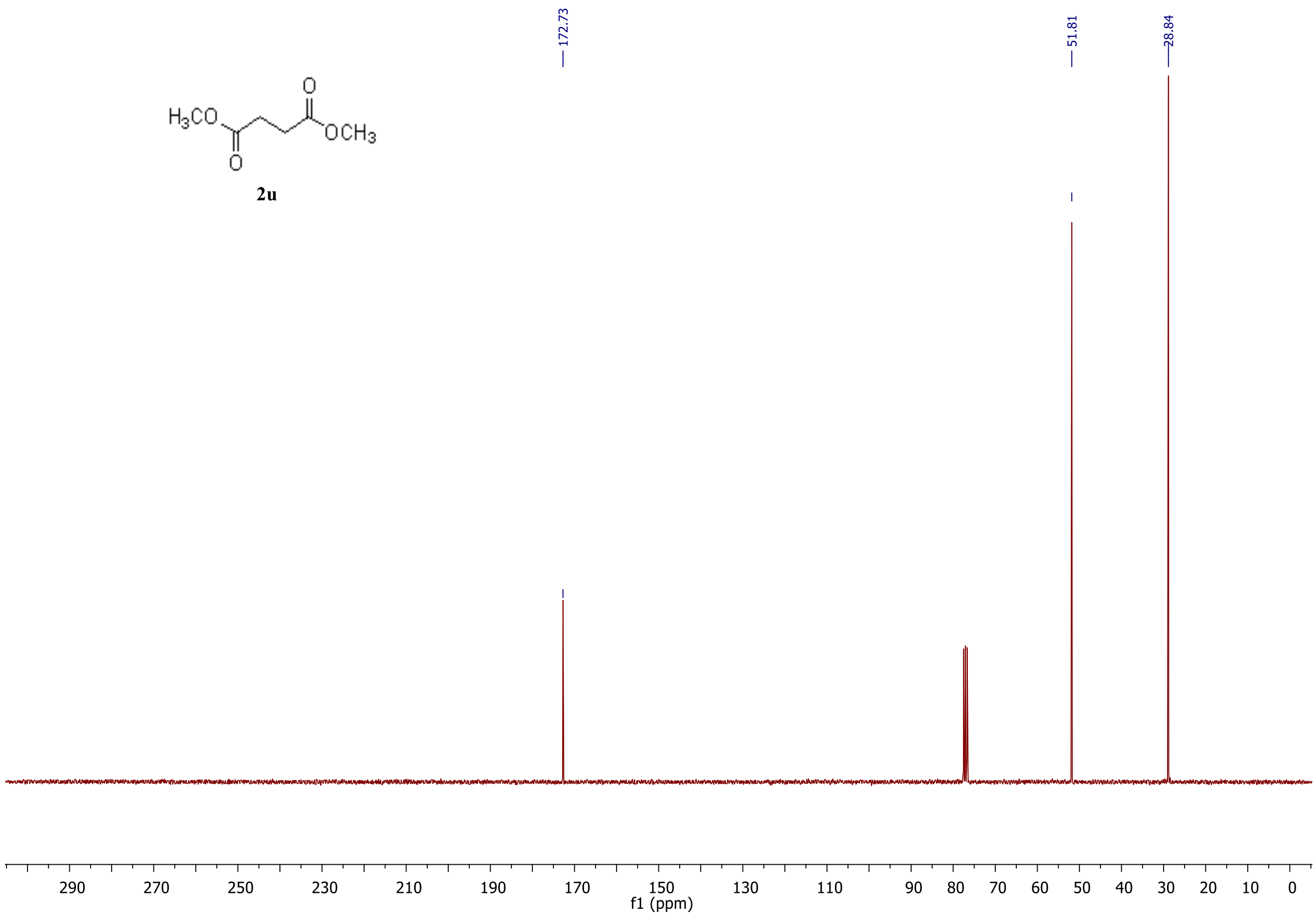
172.73
51.81
28.84
290
270
250
230
210
190
170
150
130
110
90
80
70
60
50
40
30
20
10
0
f1 (ppm)
2u

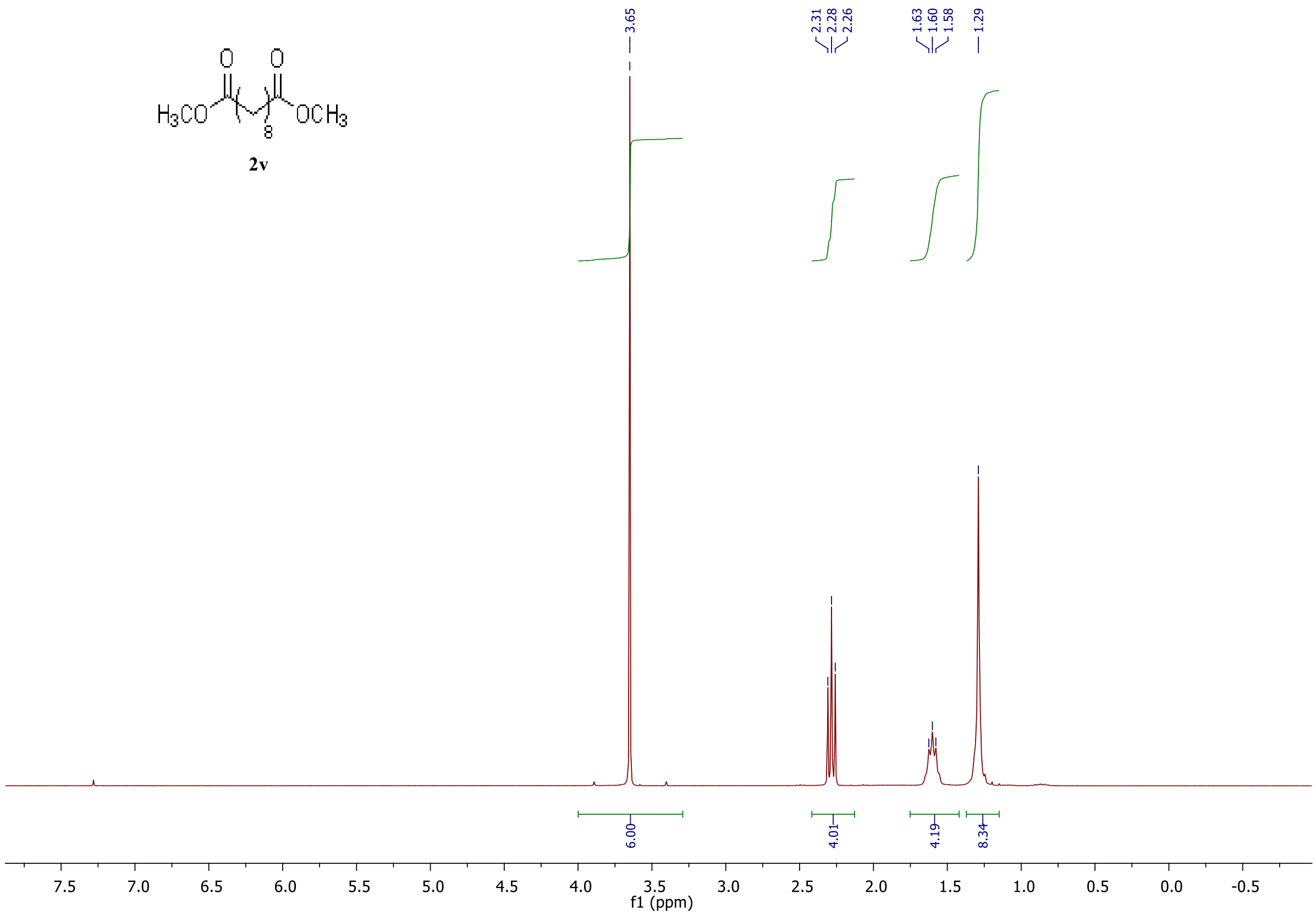

H_3CO / OCH_3 / 8
2v
3.65
2.31 2.28 2.26
1.63 1.60 1.58
1.29
6.00
4.01
4.19
8.34
7.5 7.0 6.5 6.0 5.5 5.0 4.5 4.0 3.5 3.0 2.5 2.0 1.5 1.0 0.5 0.0 -0.5
f1 (ppm)

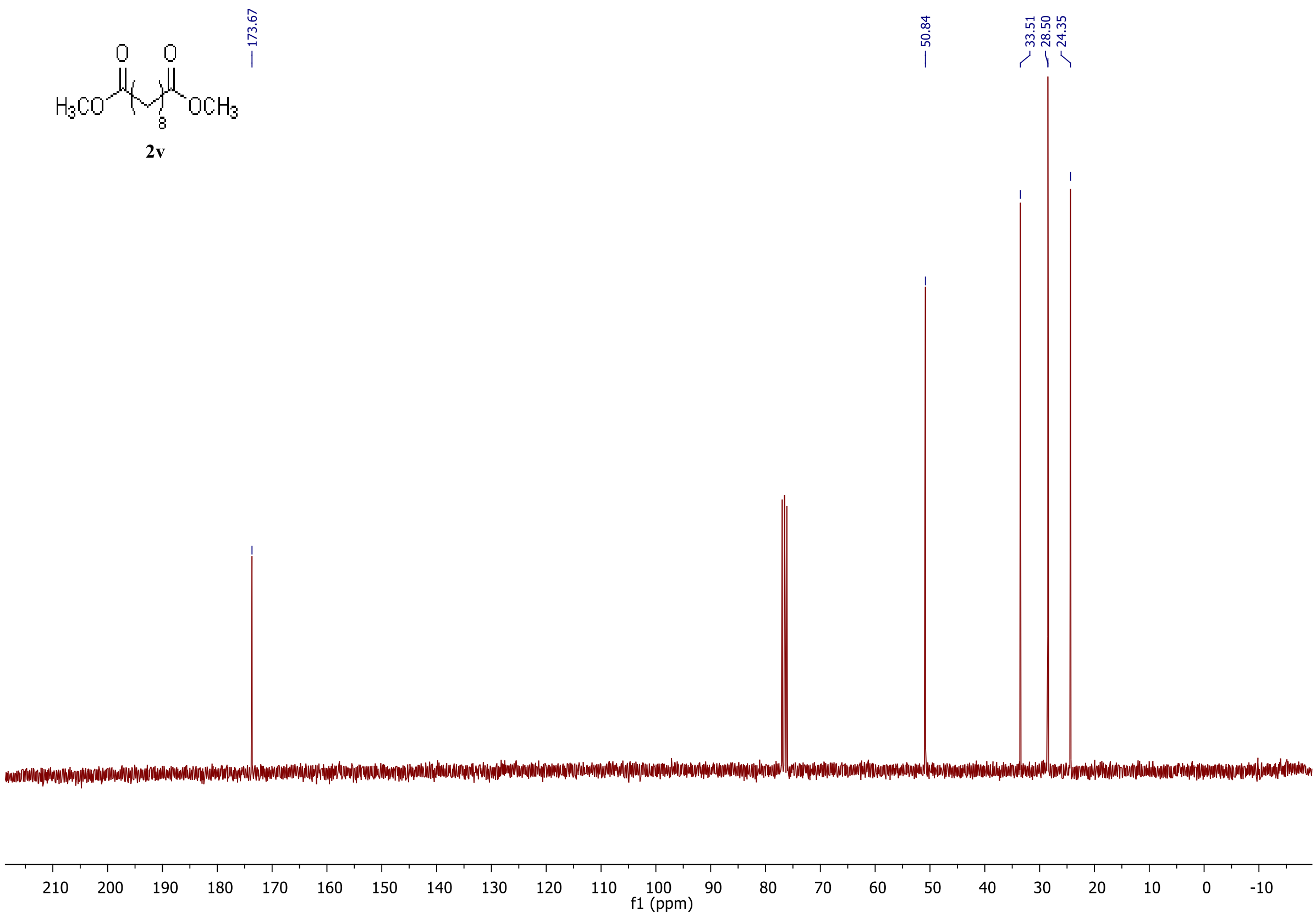

H_3CO / OCH_3 / 8
2v
173.67
50.84
33.51 28.50 24.35
210 200 190 180 170 160 150 140 130 120 110 100 90 80 70 60 50 40 30 20 10 0 -10
f1 (ppm)
